# Structural rearrangements in the nucleus localize latent HIV proviruses to a perinucleolar compartment supportive of reactivation

**DOI:** 10.1073/pnas.2202003121

**Published:** 2024-04-26

**Authors:** Fredrick Kizito, Kien Nguyen, Uri Mbonye, Meenakshi Shukla, Benjamin Luttge, Mary Ann Checkley, Anna Agaponova, Konstantin Leskov, Jonathan Karn

**Affiliations:** ^a^Department of Molecular Biology and Microbiology, School of Medicine, Case Western Reserve University, Cleveland, OH 44106

**Keywords:** HIV latency, epigenetic silencing, proviral reactivation, HIV Tat, P-TEFb

## Abstract

Using an immunofluorescence assay based on CRISPR-dCas9-gRNA complexes that selectively bind to the HIV LTR (HIV Cas-FISH), we traced changes in HIV DNA localization in primary effector T cells from early infection until the cells become quiescent as they transition to memory cells. Unintegrated HIV DNA colocalized with CPSF6 and HIV capsid (CA, p24) was found in the cytoplasm and nuclear periphery at days 1 and 3 post infection. From days 3 to 7, most HIV DNA was distributed primarily in the nuclear intermediate euchromatic compartment and was transcribed. By day 21, the cells had entered quiescence, and HIV DNA accumulated in the perinucleolar compartment (PNC). The localization of proviruses to the PNC was blocked by integrase inhibitor Raltegravir, suggesting it was due to chromosomal rearrangements. During the reactivation of latently infected cells through the T cell receptor (TCR), nascent viral mRNA transcripts associated with HIV DNA in the PNC were detected. The viral trans-activator Tat and its regulatory partners, P-TEFb and 7SK snRNA, assembled in large interchromatin granule clusters near the provirus within 2 h of TCR activation. As T cell activation progressed, the HIV DNA shifted away from the PNC. HIV DNA in latently infected memory T cells from patients also accumulated in the PNC and showed identical patterns of nuclear rearrangements after cellular reactivation. Thus, in contrast to transformed cells where proviruses are found primarily at the nuclear periphery, in primary memory T cells, the nuclear architecture undergoes rearrangements that shape the transcriptional silencing and reactivation of proviral HIV.

## Significance

Persistent viral reservoirs in memory T cells harbor latent replication-competent proviruses that can rebound after interruption of antiretroviral therapy. Although HIV proviruses integrate into transcriptionally active genes distributed throughout the genome, nuclear rearrangements during the entry of T cells into quiescence leads to the accumulation of HIV proviruses in the perinucleolar compartment in both primary cells infected ex vivo and patient-derived cells. After the reactivation of memory cells through the T cell receptor, HIV-specific transcription elongation factors assemble adjacent to the provirus. As transcription proceeds, the nucleus expands, and the proviruses shift away from the nucleolus. This work highlights dynamic changes in global 3D structures in the nucleus mediating HIV silencing and reactivation in primary T cells.

Although antiretroviral therapy (ART) potently suppresses productive HIV replication, it does not target integrated HIV proviruses that enter latency, allowing the persistence of a long-lived viral reservoir. Recent studies of proviral integration sites demonstrate that the HIV reservoir is maintained mainly through clonal expansion of memory T cells carrying silenced proviruses (1, 2). This homeostatic proliferation is counterbalanced by persistent low-level rates of viral reactivation and ensuing cell death (1–4), creating a pseudo-steady state with an apparent half-life of 44 mo (5, 6).

Most integrated proviruses (60 to 99%) in infected individuals are defective and only a limited proportion produce infectious viruses after potent T cell activation (7, 8). Based on viral outgrowth assays (QVOA), approximately one cell per million T cells in the resting memory cells compartment were estimated to be latently infected. However, more recent analyses using the intact proviral DNA (IPDA) (7–9) and the EDITS assay, which measures the production of HIV env mRNA (10–12), have revised these estimates upward to between 50 to 100 latently infected cells per million resting CD4^+^ T cells.

HIV-1 integrates into a broad but nonrandom range of sites in the host cell DNA (13, 14) specified by the viral integrase and cellular cofactors (15, 16). The lens epithelium-derived growth factor (LEDGF)/p75, a protein that binds to the nucleosomes of transcriptionally active genes, directs the HIV-1 integrase to transcriptionally active genes at the time of infection (17, 18). The cleavage and polyadenylation specificity factor 6 (CPSF6), a component of the RNA cleavage and polyadenylation machinery, mediates the nuclear import of intact viral cores (19–21) and the intranuclear trafficking of viral PICs (22, 23). Since CPSF6-capsid interactions allow the virus to bypass peripheral heterochromatin and penetrate euchromatic regions in the nucleus, it can enhance proviral integration into transcriptionally active genes (23, 24).

Because HIV tends to integrate into accessible and actively transcribing genes, latency is established and maintained through multiple cellular and HIV transcriptional control mechanisms (25). The cellular milieu required for HIV latency becomes established when infected effector T cells transition into the quiescent memory state, forcing the transcriptional silencing of the integrated provirus (11, 26). Activation of multiple cellular signaling pathways is required to induce latent proviruses, including reassembly of the positive transcription elongation factor b (P-TEFb)-containing 7SK RNP complex (27), and the nuclear translocation of the initiation factors, nuclear factor kappa B (NF-κB) (28) and the nuclear factor of activated T cells (NFAT) (29). Once transcription ceases, silencing is reinforced by epigenetic blocks of the proviral promoter, especially the histone methylation marks H3K9me3 and H3K27me3 (30). The interactions of the latent provirus and its transcription initiation factors and the Tat/7SK snRNP/P-TEFb machinery required for transcription elongation have generally been assumed to be stoichiometric and stochastic (25).

How these critical transcriptional elongation factors assemble at the latent proviruses after activation of CD4^+^ T cells is undefined. We hypothesized that the drastic cellular changes that occur as CD4^+^ T cells transition between effector and memory phenotypes would affect the intranuclear location of the provirus and its interactions with regulators of transcriptional reactivation. Transcriptionally active wild-type proviruses in transformed cell lines were found to be closer to the NE than expected by random chance (20, 31). In these model systems, the distance from the NE did not correlate with transcriptional activity, and transcriptionally active proviruses were randomly distributed after several cell divisions, suggesting that the intranuclear locations of proviruses are dynamic (20).

To visualize proviral localization in the nucleus in primary cells, we adapted the CRISPR-Cas9-mediated in situ labeling of genomic loci (Cas-FISH) technique (32). We combined this with the fluorescent detection of HIV RNA transcripts and transcription factors. These techniques allowed us to monitor the spatiotemporal dynamics of HIV proviruses from an acute infection to the establishment of latency and the subsequent reactivation of transcription in the QUECEL primary cell model of HIV latency (11, 27). The results show that, in contrast to transformed cell lines (20, 31), in quiescent primary T cells, HIV DNA accumulates in a specific perinucleolar compartment (PNC) where the provirus is transcriptionally silenced but poised for efficient reactivation.

## Results

### Detection of HIV Proviruses by Immuno-Cas-FISH Imaging.

**Fig. 1.**
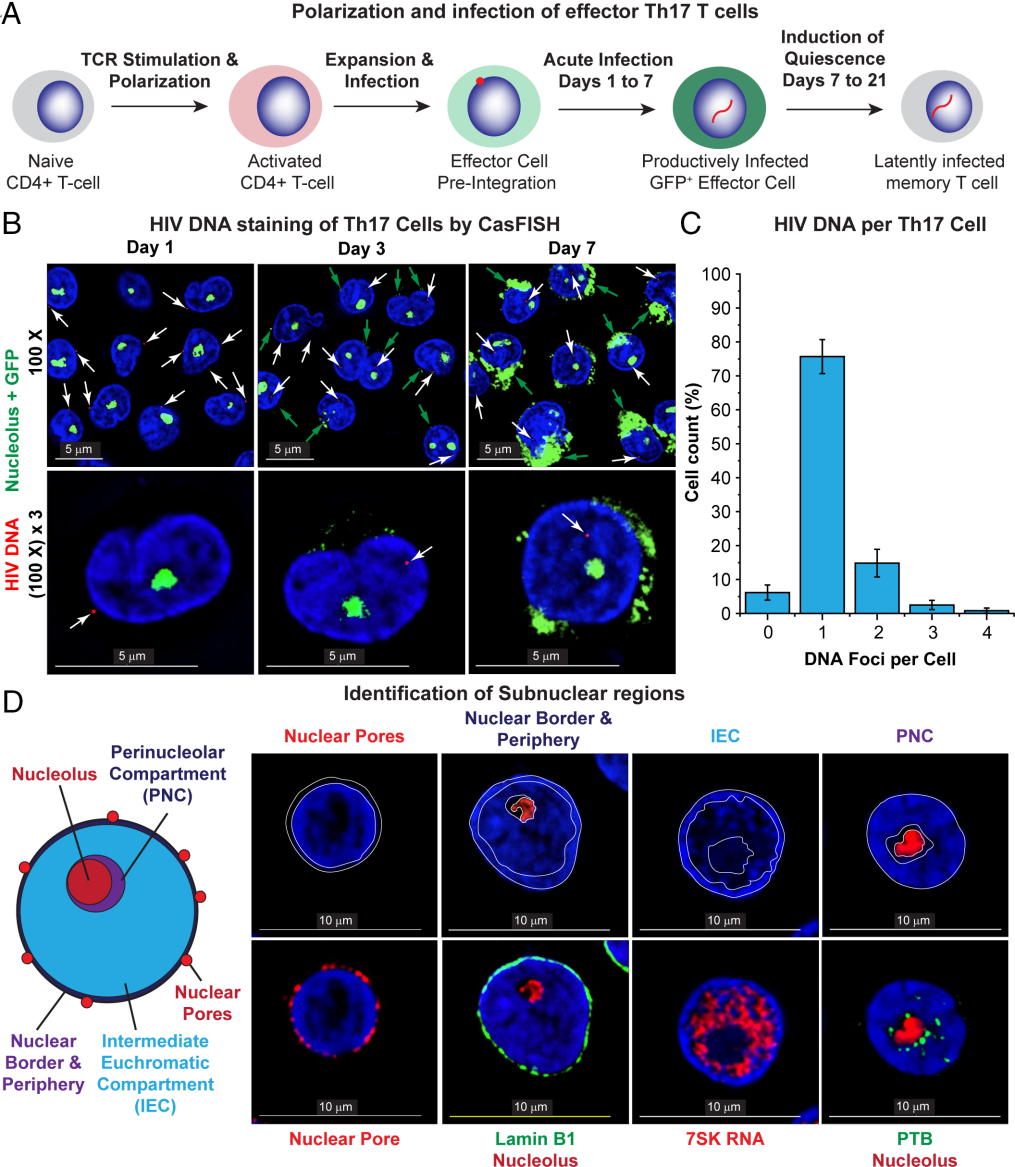
CasFISH staining of HIV-infected primary Th17 cells. (*A*) Experimental design for generating latently infected primary Th17 quiescent effector cells using the QUECEL procedure. (*B*) Detection of HIV DNA in acutely infected primary Th17 cells by CasFISH. White arrows point to the location of HIV DNA (in red) detected by Alexa Fluor 594 immunofluorescence staining of an HIV-LTR gRNA-directed dCas9. Green: Nucleoli detected using a fluorescein-labeled 45S pre-rRNA intron-specific FISH probe set. Blue: DNA stained using DAPI. pi: post infection. (Scale bar, 5 μm.) (*C*) The number of HIV proviruses detected in infected Th17 cells at day 7. Plotted data are from three independently obtained sets of micrographs, with each group containing 100 randomly selected infected cells. Error bars: SD. (*D*) Diagram showing the scheme used to determine the subnuclear location of HIV DNA. *Right*: Micrographs showing the compartmentation of the nucleus based on immunofluorescence staining (nuclear pore complex, Lamin B1 and PTB) and RNA FISH (7SK RNA and nucleolus). (Scale bar, 10 µm.)

To identify HIV DNA by high-resolution fluorescence microscopy, we modified the Cas-FISH imaging technique (32) (Fig. 1 and *SI Appendix*, Fig. S1). Catalytically inactivated Cas9 (dCas9) was directed to the U5 region of the viral long terminal repeat (LTR) by guide RNAs and subsequently detected by a Cas9-specific primary antibody and a fluorescently labeled secondary antibody. To amplify the immunofluorescence signal, six specific LTR guide RNA sequences (20 nt) from the HXB2 reference genome were arrayed in tandem (*SI Appendix*, Fig. S1*A*). The guide sequences were complexed with purified dCas9 protein (*SI Appendix*, Fig. S1*B*) through a tracrRNA sequence (Alt-R® CRISPR-Cas9 tracrRNA) to form a stable complex that could be incubated with fixed and permeabilized cells.

We demonstrated the specificity of the immuno-Cas-FISH system and the lack of off-target binding using uninfected Jurkat T cells (E6 clone) and the HIV-infected clones 2D10 (two proviruses) and 3C9 (one provirus) (28, 33, 34) (*SI Appendix*, Fig. S1*C*). Using the immuno-Cas-FISH system, we consistently detected no integrated proviral HIV DNA puncta in the E6 cells (98.3% negative; *SI Appendix*, Fig. S1 *C* and *D*), whereas, as expected, the majority of the 3C9 (82%) and the 2D10 (76.7%) cells contained 1 or 2 integrated proviral HIV DNA puncta, respectively (*SI Appendix*, Fig. S1 *C* and *D*). Because the Jurkat cells were actively dividing, a small proportion of the 3C9 cells (11%) had two proviruses, and a similar proportion of 2D10 cells (12.5%) had four proviruses, but no cells were detected carrying three proviruses. Only 19% of the 3C9 or 2D10 cells were negative for HIV proviruses. This could either be due to the loss of these loci during replication of the transformed host cell line or to minor detection inefficiencies in the Cas-FISH assay.

Since denaturation of the chromatin is not required, the Cas-FISH imaging method maintains the nuclear architecture and can be readily combined with other fluorescent detection methods to localize proteins and cellular and viral RNA. To confirm that the HIV DNA represented sites of transcriptionally competent integrated proviruses, we combined Cas-FISH detection of the proviruses and in situ hybridization using TAMRA-labeled Stellaris DNA oligonucleotide probes (RNA FISH probes) derived from the LTR to detect total HIV mRNA transcripts. As shown in *SI Appendix*, Fig. S1*C* (*Bottom*), after induction of 3C9 cells for 24 h with 10 ng/mL TNF-α, HIV mRNA transcripts colocalizing with the proviruses were detected in addition to transcripts distributed throughout the nucleus and the cytoplasm.

We next used Cas-FISH to monitor the distribution of unintegrated and integrated HIV DNA after acute infection of Th17 cells and as the cells transitioned from proliferating effector cells to latently infected quiescent memory T cells, using the QUECEL protocol (11, 35) (Fig. 1*A*). The QUECEL experiments were conducted using single round viruses that carried a CD8α-d2EGFP fusion protein to permit purification of infected cells, or in mixed populations of cells using a simple d2EGFP reporter (*SI Appendix*, Fig. S2 *A* and *B*). Consistent with previous reports using transformed cells (20), at day 1 post infection (pi), HIV DNA was primarily detected near the nuclear periphery (Fig. 1*B*). In the enriched population of infected cells following CD8a positive selection at day 7, 77% of the cells had a DNA locus, 14% had two loci and 6% of the cells appeared to be uninfected (Fig. 1*C*).

### Identification of Subnuclear Compartments.

To rigorously define different subnuclear regions, we used a variety of antibodies and RNA markers (Fig. 1*D*). Examples of proviruses located in each region are shown in *SI Appendix*, Fig. S2*C*. i) Nuclear border and periphery: The nuclear border and periphery was identified by immunostaining for the nuclear pore complex (Alexa Fluor® 594 anti-nuclear pore complex proteins antibody) and Lamin B1, which forms an anchoring scaffold of the genome at the nuclear periphery (36). Although actively transcribing viruses have been reported at the nuclear periphery (20), the bulk of the chromatin in contact with the nuclear lamina is composed of high-density DNA and enriched in transcriptionally repressed regions constituting a distinct subnuclear compartment (37). ii) Nucleolus: The nucleolus was visualized by in situ hybridization with Fluorescein-labeled Stellaris oligonucleotide probes to the 45S pre-rRNA introns. The specificity of the RNA FISH probes was confirmed by immunofluorescence costaining with the nucleolus-specific marker fibrillarin (*SI Appendix*, Fig. S3*A*) (38). iii) Intermediate euchromatic compartment (IEC): the IEC of the nucleus in activated T cells is a region of variable but relatively low DNA density (*SI Appendix*, Fig. S4), containing transcriptionally active euchromatin. This region becomes filled with nuclear transcription factors, as exemplified in Fig. 1*D* by 7SK snRNA, the scaffold for the P-TEFb transcription factor. The dramatic contraction of the nucleolus and the more subtle reduction in nuclear size as cells enter quiescence and their re-expansion after quiescent T cells are activated by stimulation of the T cell receptor (TCR) is shown in *SI Appendix*, Fig. S3 *B*–*F*. iv) PNC: the PNC is a dynamic structure and is highly enriched in RNA-binding proteins, such as the polypyrimidine tract-binding protein (PTB) (39) (Fig. 1*D*). The PNC characteristically displays a much lower DNA density than other nuclear subcompartments, suggesting it is highly enriched in euchromatin and supports active transcription and splicing (*SI Appendix*, Fig. S4). Operationally, we identify HIV DNA as localized to the PNC when it is in a region of low DNA density found within 0.35 μm of the nucleolar border.

### Timing of HIV Integration, Transcription, and Quiescence.

The extent of HIV integration and transcription was monitored from day 1 through to the entry of the cells into quiescence from days 7 to 21, a process that drives the integrated proviruses into latency. Flow cytometry and immunofluorescence were used to measure d2EGFP, HIV Tat expression (*SI Appendix*, Fig. S5). Using the CD8a-d2EGFP reporter, HIV expression could be detected as early as day 2 and reached maximal intensity at day 7 (*SI Appendix*, Fig. S5*A*). However, the fraction of cells expressing the d2EGFP reporter reached maximal levels by day 4 (*SI Appendix*, Fig. S5*B*). HIV Tat (*SI Appendix*, Fig. S4*C*) correlated with d2EGFP expression until day 7 suggesting that a fraction of cells had become latently infected even at this early stage.

Direct measurements of HIV integration were obtained using an Alu-ψ PCR assay. There was a good agreement between estimates of the fraction of cells carrying integrated proviruses and the fraction of cells expressing GFP for both the pHR′-CD8a-d2EGFP and pHR′-d2EGFP reporter viruses (*SI Appendix*, Fig. S6 *A* and *C*). However, prior to the peak of GFP expression at day 4, more integrated DNA could be detected, consistent with GFP expression being a lagging marker. HIV RNA expression peaked between days 3 and 4 and declined rapidly as cells entered quiescence (*SI Appendix*, Fig. S6*B*).

The transition of the cells into quiescence by day 21 was also confirmed by flow cytometry using the cell cycle progression markers cyclin D3 and Ki67 (11, 35, 40, 41) (*SI Appendix*, Fig. S7). In this experiment, CD8a-selection was used to enrich for cells expressing CD8a-d2GFP prior to shutdown and reactivation.

### Redistribution of HIV DNA during the Transition of T Cells into Quiescence.

**Fig. 2.**
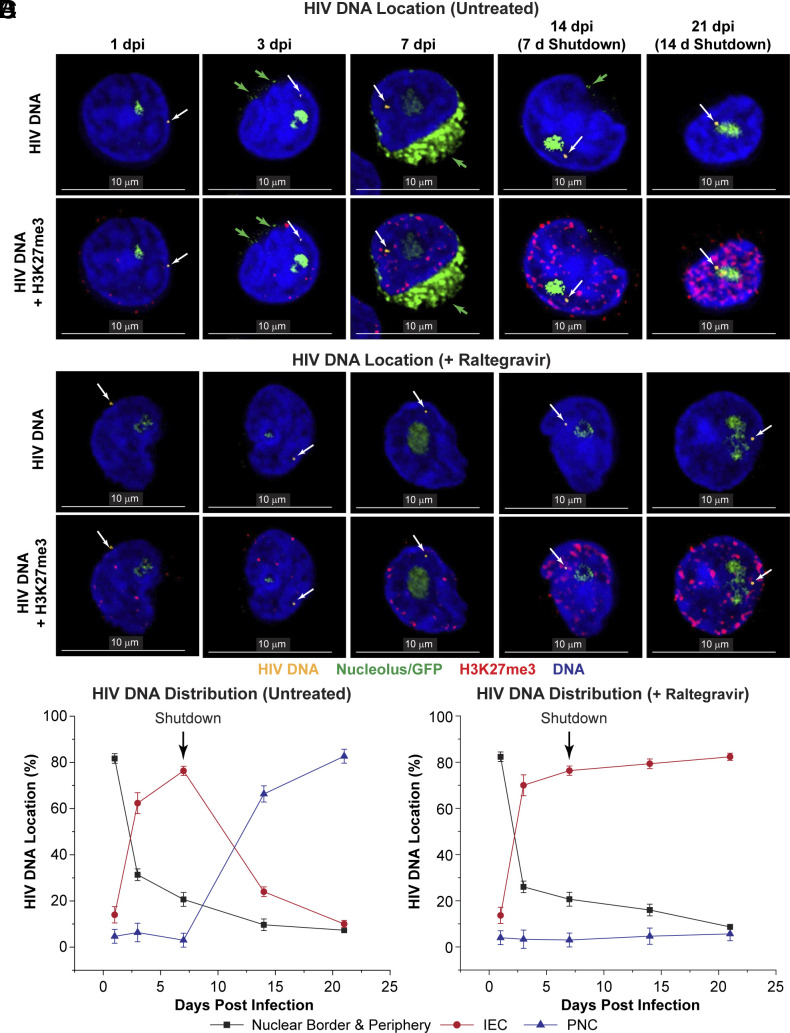
Subnuclear distribution of HIV DNA during infection and entry of T cells into quiescence. (*A*) Polarized Th17 cells were infected with the CD8a-GFP reporter in the absence of Raltegravir (RALT). (*B*) Cells treated with 10 μM RALT. CasFISH was used to detect the location of HIV DNA (stained in yellow and indicated by white arrows). This was combined with immunofluorescence staining of histone H3 Lys27 trimethylation (H3K27me3; red) and nucleolar staining by 45S pre-rRNA intron-specific FISH (green). The green arrows show the CD8a-GFP in the ER, which is present due to active proviral gene expression at 7 d postinfection. (Scale bar, 10 μm.) (*C*) Quantification of the subnuclear distribution of HIV DNA over the period of the QUECEL procedure in untreated cells. (*D*) Subnuclear distribution of HIV DNA in cells treated with 10 μM RALT. Black arrows indicate the initial time point of the quiescence phase. Error bars: SD.

Fig. 2*A* shows the distribution of HIV DNA detected by Cas-FISH in cells infected with the pHR′-CD8a-d2EGFP reporter throughout the time course. HIV DNA was detected primarily at the nuclear periphery at day 1 (Fig. 2*A*). A random sampling of images from three different datasets (each constituting a total of 100 cells containing a single HIV DNA locus) was used for statistical data analysis of the HIV DNA distribution in this, and all subsequent studies (Fig. 2*C*). At day 1, 82% of the HIV DNA was detected at the nuclear border and periphery (Fig. 2 *A* and *C*) (19, 23, 24), while only 19% was within the IEC [i.e., confined within the lower-density DAPI regions away from the periphery (*SI Appendix*, Fig. S4)]. At day 3 pi, 62% of the HIV DNA was distributed throughout the IEC, and 6% was present in the PNC. At day 7 pi, 76% of HIV DNA was detected in the IEC (Fig. 2 *A* and *C*). As the cells entered quiescence at day 14, 66% of HIV DNA was detected in the PNC (Fig. 2 *A* and *C*). This increased to 82% by day 21, when the cells were entirely quiescent.

Visualization of the HIV DNA does not by itself indicate whether individual DNA puncta represent a functional integrated provirus. To estimate the frequency of integration events on a per cell basis, we identified cells carrying HIV DNA that also expressed d2EGFP detected by fluorescence microscopy. In the experiment shown in *SI Appendix*, Fig. S8, which was performed using the pHR’-d2EGFP reporter virus, we included counts of HIV DNA appearing in the cytoplasm. As shown in *SI Appendix*, Fig. S8*B*, none of the cells containing HIV DNA in the cytoplasm expressed d2EGFP. By contrast, over 80% of the cells containing HIV DNA in the IEC and PNC expressed d2GFP at days 3 and 7, which indicated that they were integrated proviruses. As expected, after day 7, the fraction of d2EGFP-expressing cells diminished as the cells entered latency. By contrast, in the nuclear border and periphery 60% of the cells expressed d2EGFP by day 7 implying that the DNA in this region represented a mixture of unintegrated, transcriptionally inactive HIV DNA and integrated transcriptionally active proviruses.

Changes in DNA density and histone methylation also accompany the transition of cells into quiescence. In the activated effector cells, proviruses are found primarily in euchromatic regions characterized by relatively low DAPI density (*SI Appendix*, Figs. S3 and S9). As the cells are forced into quiescence, the DNA and nucleolus become much more compacted, but HIV DNA found in the PNC remain in regions of comparatively low DNA density (*SI Appendix*, Figs. S4 and S9). These changes in DNA density were accompanied by dramatic and progressive increases in H3K27me3 histone methylation at the HIV DNA loci in control cells (Fig. 2*A* and *SI Appendix*, Fig. S9*B*), but H3K27me3 did not increase at HIV DNA loci in RALT-treated cells (*SI Appendix*, Fig. S9*B*).

### HIV DNA Accumulation in the PNC Is Dependent upon Integration.

To determine whether the HIV DNA accumulation in the PNC was due to postintegration chromatin rearrangements during quiescence, parallel experiments were performed in the presence of the integrase inhibitor, Raltegravir (RALT, 10 μM) (Fig. 2*B*). RALT did not affect the HIV DNA distribution at the periphery and in the IEC during the first seven days, but it blocked the accumulation of proviruses in the PNC as the cells become quiescent (Fig. 2 *B* and *D*). For example, at day 21, 82% of the HIV DNA was found in the IEC and only 6% was found in the PNC after RALT treatment, whereas in untreated control cells, 83% of the HIV DNA was found in the PNC and only 10% was found in the IEC (Fig. 2 *C* and *D*).

As controls for RALT activity, the impact of the drug on HIV integration was measured by the Alu-ψ PCR assay (*SI Appendix*, Fig. S6 *A* and *C*), GFP expression measured by flow cytometry (*SI Appendix*, Fig. S6 *A* and *C*), RNA expression measured by PCR (*SI Appendix*, Fig. S6*B*), and the kinetics of 2-LTR circle formation was monitored by qPCR (*SI Appendix*, Fig. S6*D*) (42). As expected, 10 μM RALT efficiently blocked integration, GFP and RNA expression, demonstrating that integration was required for HIV transcription from these reporters, which lack Vpr (43). In untreated cells, 2-LTR circles, which are a product of reverse transcription and cellular nonhomologous DNA end-joining activity (44), were found at high levels at days 1 and 3 (*SI Appendix*, Fig. S6*D*) and then was lost during entry of the cells into quiescence. The 2-LTR circles, which are one species of nonintegrated HIV DNA products, persisted in the RALT-treated cells, consistent with the detection of unintegrated HIV DNA in these cells by Cas-FISH (Fig. 2 *B* and *D*). However, the fraction of cells carrying HIV DNA detected by Cas-FISH after RALT treatment declined to less than 30% as cells became quiescent suggesting that there was a loss of all forms of unintegrated DNA (*SI Appendix*, Fig. S9*C*).

### Colocalization of HIV-1 DNA and CPSF6.

**Fig. 3.**
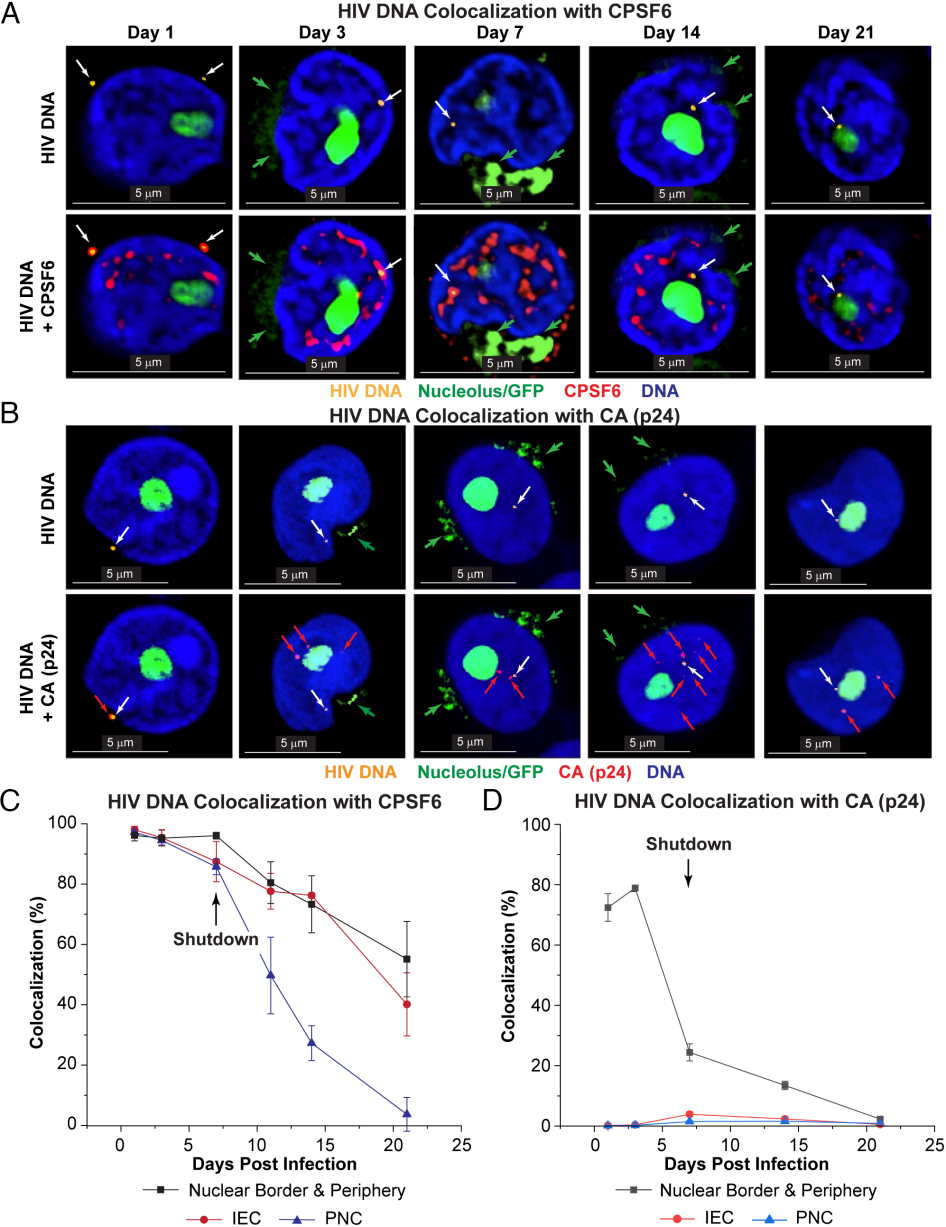
Localization of HIV DNA relative to CPSF6 and p24 after acute infection and during the transition of T cells into quiescence. (*A*) HIV DNA CasFISH combined with immunofluorescence staining of CPSF6 and 45S pre-rRNA FISH. Cells were infected with the CD8a-d2EGFP reporter virus. Detection of HIV DNA (*Top* and *Bottom* panels; yellow) and colocalization with CPSF6 (*Bottom* panels; red). Nucleolar staining with 45S pre-rRNA intron-specific FISH is shown in green. (*B*) HIV DNA CasFISH combined with immunofluorescence staining of HIV CA (p24). Detection of HIV DNA (*Top* panels; red) and colocalization with CA (*Bottom* panels; green). Nucleolar staining with 45S pre-rRNA intron-specific FISH is shown in yellow. White arrows indicate the location of the HIV DNA. Green arrows in *A* indicate GFP produced as a result of active HIV expression at 7 dpi. Light green arrows in *B* point to the detection of p24. Images in both *A* and *B* were taken at 100×. (Scale bar, 5 μm.) (*C*) Colocalization of HIV proviral DNA with CPSF6. (*D*) Colocalization of HIV proviral DNA with CA. Error bars: SD.

We also monitored changes in the colocalization of HIV DNA and CPSF6 (Fig. 3 *A* and *C* and *SI Appendix*, Fig. S10) during infection and entry of cells into quiescence after infection with the pHR′-CD8a-d2EGFP virus. CPSF6 binds directly to the viral cores at specific sites on the capsid (45–47). The interaction between the cellular CPSF6 and CA enhances the transport of HIV DNA from the nuclear periphery into regions where integration can occur efficiently (19, 21, 23, 48).

As shown in the high-resolution images (Fig. 3*A*) and broader field images (*SI Appendix*, Fig. S10*A*), during the first 7 d, more than 95% of the detected HIV DNA colocalized with CPSF6. However, as the cells entered quiescence and viral transcription shutdown, there was a progressive reduction in CPSF6 colocalizing with HIV DNA. The relative abundance of HIV DNA and CPSF6 (Fig. 3*C*) was measured by comparing the fluorescent intensity ratios at the provirus from line scans taken through the individual cellular images (*SI Appendix*, Fig. S11). For example, on day 14, only 27% of HIV DNA in the PNC was colocalized with CPSF6, whereas 76% of the HIV DNA in the IEC was colocalized with CPSF6. By day 21, when the cells were entirely quiescent, only 3.8% of the HIV DNA in the PNC was located near CPSF6 (Fig. 3*C*). It is notable that CPSF6 is distributed in a punctate pattern in the nucleus of cells before HIV infection and increases between day 1 and 3. However, it is substantially upregulated in HIV-infected cells at day 3 (*SI Appendix*, Fig. S12).

A complementary way of expressing the data is to calculate the Pearson correlation coefficients (r) for colocalization of the HIV DNA and CPSF6 (*SI Appendix*, Fig. S10*B*). Colocalization is highly significant at day 1 and day 7 pi (r >0.93). However, as the cells shut down and enter quiescence, colocalization declines and becomes insignificant (Fig. 3*C* and *SI Appendix*, Fig. S10*B*).

### Colocalization of HIV-1 DNA and Capsid (p24).

The pattern of CA (p24) colocalization with HIV DNA diverged from that of CPSF6 (Fig. 3*B*). In the cytoplasm, 93% to 97% of the HIV DNA was found in association with CA (p24) (93% to 97%) (*SI Appendix*, Fig. S13). During the first 3 d, HIV DNA found at the nuclear border and periphery remained highly associated with CA (p24) (72 % at day 1; 79 % at day 3) (Fig. 3*D*). However, by day 7 only 24% of the HIV DNA near the periphery remained associated with CA (p24), consistent with the dissociation of capsids after entry of the PICs into the nucleus and during the integration of the HIV provirus. By day 21, only 2% of the HIV DNA in the nuclear periphery remained associated with CA (p24) (Fig. 3*D*). By contrast, at each time point, there was no significant association of CA (p24) with HIV DNA in the IEC and PNC (Fig. 3*D*) and CA (p24) was detected dispersed throughout the IEC as distinct and separate signals from the HIV DNA (Fig. 3*B*).

Similar results were seen in a second experiment (*SI Appendix*, Fig. S13) performed using the pHR′-d2EGFP virus. As before, HIV DNA associated with CA was seen at the nuclear border and nuclear periphery. We further classified the HIV DNA as being at the DNA-dense region at the nuclear border or in the less dense DNA regions in the nuclear periphery (*SI Appendix*, Fig. S14*A*) and measured d2EGFP expression in these cells. HIV DNA colocalizing with CA was only found at the nuclear border and none of the cells expressed d2EGFP (*SI Appendix*, Fig. S14*B*). By contrast, HIV DNA dissociated from CA was found in the nuclear periphery and each of these cells expressed d2EGFP, suggesting the HIV DNA had integrated (*SI Appendix*, Fig. S14*C*). The CA (p24) which was dissociated from the HIV DNA remained associated with CPSF6, which may contribute to its slow turnover in the nucleus (*SI Appendix*, Fig. S15).

### The PNC Is a Site of Preferential Transcription.

**Fig. 4.**
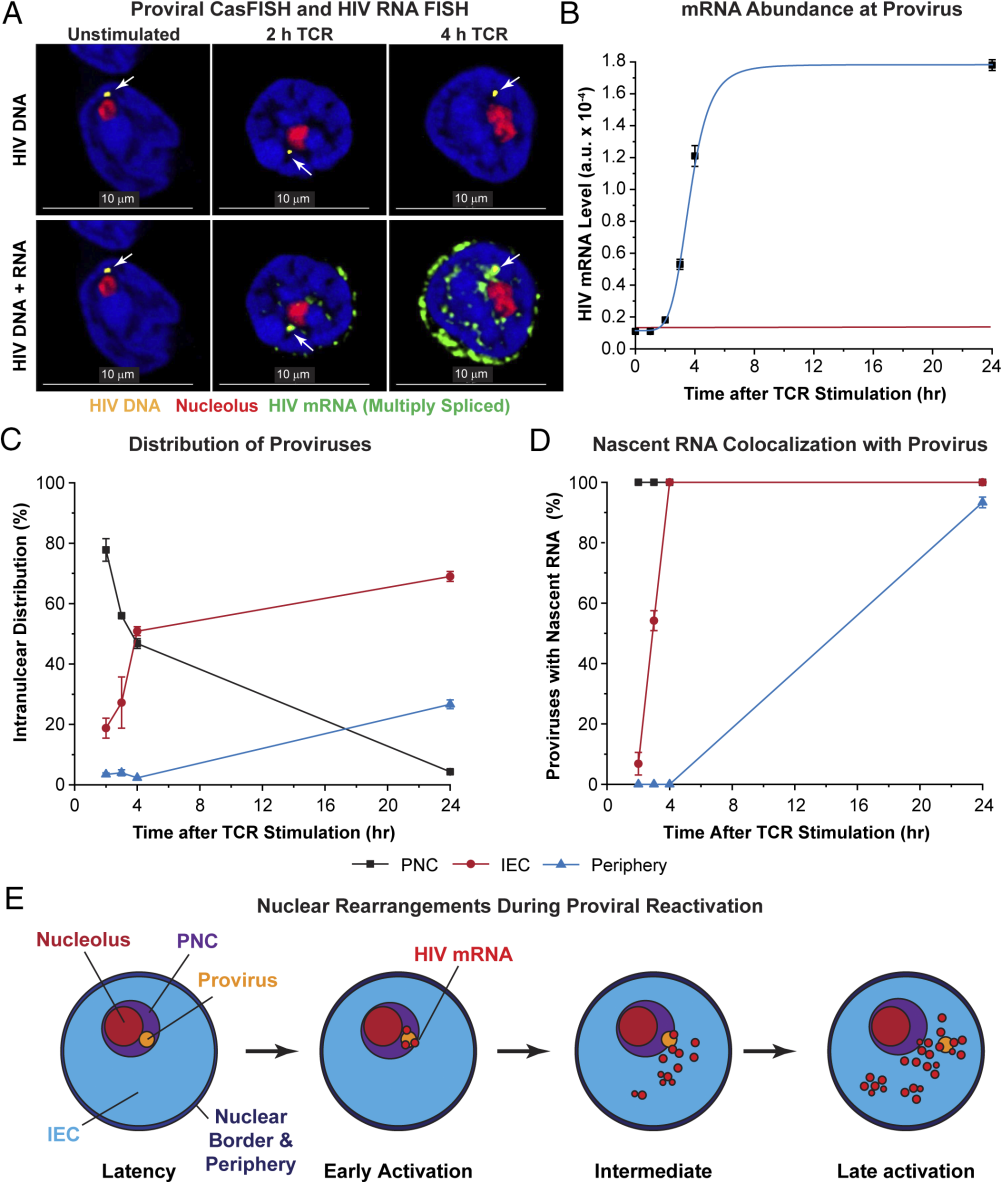
Synthesis of nascent multiply spliced HIV mRNA occurs in the PNC upon reactivation of latently infected cells. (*A*) Quiescent, latently infected cells infected with the CD8a-d2EGFP reporter (and purified at day 7) were reactivated through the TCR and then subjected to both HIV DNA CasFISH and RNA FISH with probe sets specific toward multiply spliced HIV RNA and 45S pre-rRNA. White arrows point to the location of HIV proviruses (yellow). HIV mRNA and 45S pre-rRNA are shown in green and red, respectively. Images were taken at 100× and then enlarged threefold. (Scale bar, 10 μm.) (*B*) Relative fluorescent intensity measurements of HIV mRNA levels at the HIV proviral DNA locus. Error bars: SD. Red line: Threshold of fluorescence detection. (*C*) Intranuclear distribution of proviruses after TCR stimulation. (*D*) Colocalization of nascent RNA with HIV proviruses. (*E*) Proposed scheme for the nuclear rearrangements after T cell activation showing the movement of proviruses away from the nucleolus and the initial detection of nascent HIV RNA in the PNC.

Stimulation of the quiescent T cells by activating the TCR initiates HIV transcription as the cells transition back into activated effector cells. To examine the impact of provirus localization on HIV reactivation, new RNA synthesis was monitored following TCR activation using fluorescently labeled Stellaris RNA FISH probes (Fig. 4). HIV RNA transcripts were initially seen in the PNC before they dispersed throughout the nucleus and entered the cytoplasm (Fig. 4*A* and *SI Appendix*, Fig. S16*A*). HIV RNA that colocalized with the provirus was detected by 2 h after TCR stimulation and reached saturation by 8 h (Fig. 4 *A* and *B* and *SI Appendix*, Fig. S16*B*). HIV RNA remained associated with the provirus for at least 24 h and also accumulated in punctate structures in the nucleus and the cytoplasm.

After T cell reactivation, there was a dramatic expansion of the area of the nucleolus, which progressively increased from 0.54 ± 0.15 µm^2^ in the quiescent T cells to a maximal size of 2.73 ± 0.40 µm^2^ at 24 h (*SI Appendix*, Fig. S4*D*). These rearrangements in the nuclear architecture were associated with a gradual shift of the proviruses away from the nucleolus (Fig. 4*C* and *SI Appendix*, Fig. S17*A*). In some z-sections proviruses seen in the quiescent cells were so close to the nucleolus that it was hard to determine whether they were separated from the nucleolus. However, as shown in *SI Appendix*, Fig. S17*B*, by reconstructing 3D images from z-stacks and observing the nuclei at different angles, we were able to confirm that proviruses that appeared to overlap with the nucleolus in a particular z-stack section were localized to the PNC.

As shown in Fig. 4*C*, 75% of HIV DNA loci were detected in the PNC of quiescent T cells, whereas by 4 h, 51% of the proviruses were found in the IEC, and only 47% remained in the PNC. By 24 h, when the cells are fully reactivated and beginning to reenter the cell cycle, 69% of the proviruses were found in the IEC. All the proviruses found in the PNC produced HIV RNA from 2 h onward (Fig. 4*D*), indicating that the PNC became a specialized subnuclear compartment supportive of high levels of early transcription and RNA processing shortly after activation of the latently infected cells. Consistent with their location in relatively DNA-dense regions, the proviruses initially found in the IEC showed delayed reactivation compared to proviruses found in the PNC and only became fully reactivated after 4 h (Fig. 4*D*). Similarly, the few proviruses initially found in the DNA-dense periphery remained transcriptionally inactive during the first 4 h after TCR stimulation, suggesting that they were in repressed heterochromatic regions.

### 7SK snRNA Colocalizes with the HIV Proviruses.

**Fig. 5.**
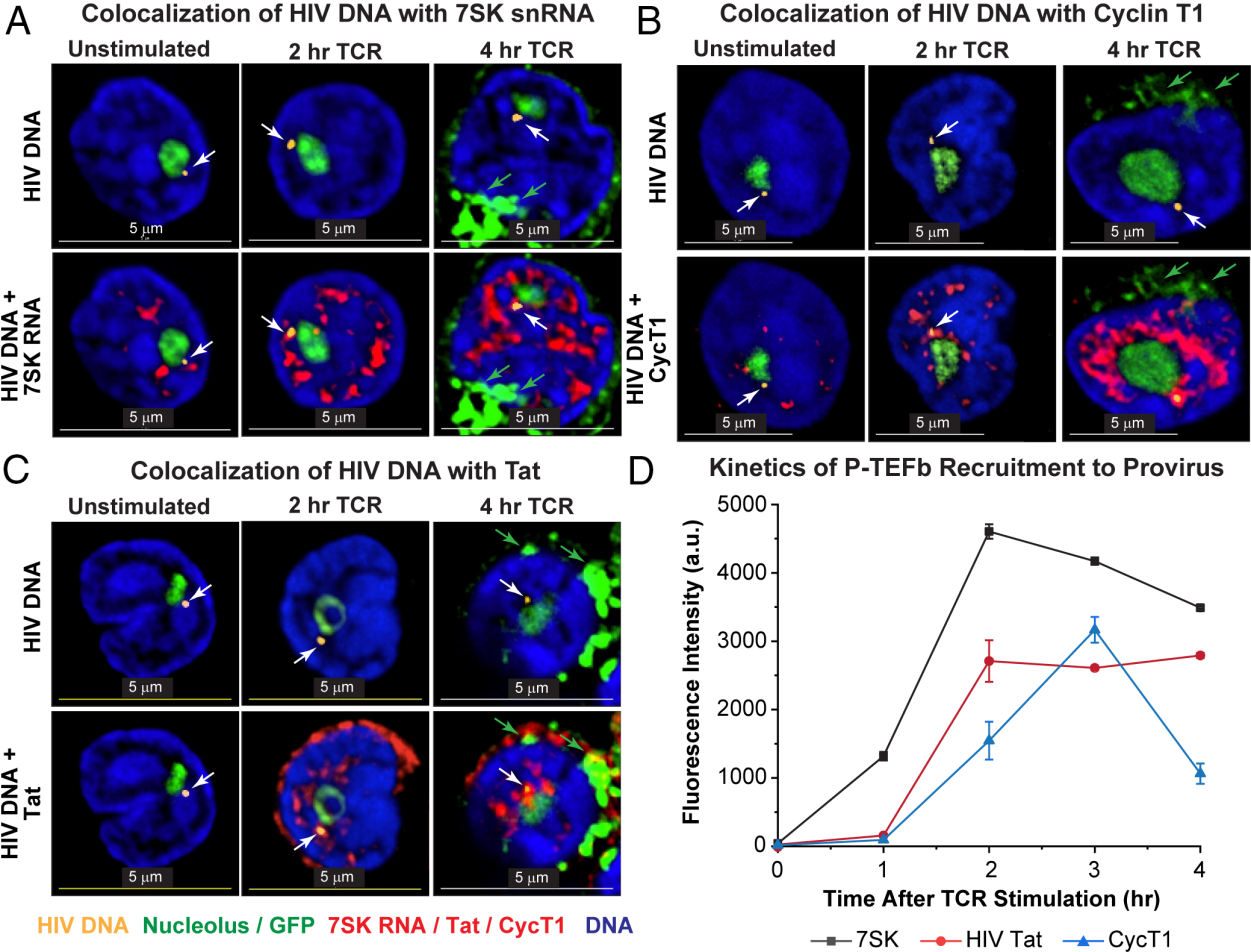
Colocalization of HIV proviruses with the P-TEFb transcription elongation factor machinery. Latently infected primary Th17 cells infected with the CD8a-GFP reporter (and purified at day 7) were reactivated through the TCR for the times shown before performing CasFISH to detect HIV RNA (yellow staining highlighted by white arrows). (*A*) RNA FISH using Quasar 670-labeled Stellaris oligonucleotide probes directed to 7SK snRNA (red). (*B*) Immunofluorescence staining for CycT1 (red). (*C*) Immunofluorescence staining for HIV Tat (red). Nucleoli were stained using a fluorescein-labeled 45S pre-rRNA intron-specific FISH probe set in all experiments. Green arrows indicate GFP produced from HIV transcription in cells activated for 4 h. (Scale bar, 5 μm.) (*D*) Graph showing the colocalization of 7SK snRNA (black symbols), HIV Tat (red symbols), and cyclin T1 (CycT1, blue symbols) at the proviral loci in unstimulated cells and up to 4 h following TCR activation. Error bars: SD.

7SK snRNA, the molecular scaffold for the 7SK snRNP complex, controls the delivery of the cellular transcription elongation factor P-TEFb to the HIV provirus (25). TCR stimulation led to the progressive nuclear induction of 7SK snRNA as detected by in situ hybridization using Quasar 670-labeled Stellaris oligonucleotide probes. In quiescent cells, there were relatively low levels of 7SK snRNA which was dispersed in discrete puncta throughout the nuclei, and there was no association of 7SK snRNA with HIV proviruses (Fig. 5*A* and *SI Appendix*, Fig. S18). After TCR activation, there was a massive and progressive increase in 7SK snRNA. Low levels of 7SK snRNA were initially distributed in puncta located in the PNC and IEC. Colocalization of 7SK snRNA with the proviruses in the PNC was detected 2 h after TCR activation (Fig. 5*A* and *SI Appendix*, Fig. S18). In the fully activated cells, 7SK snRNA formed a characteristic annulus surrounding the nucleolus and accumulated in regions of low DNA density in the IEC (Fig. 5*A* and *SI Appendix*, Fig. S18*A*).

### Recruitment of P-TEFb and HIV Tat to Proviruses After TCR Activation.

Using a combination of Cas-FISH and immunostaining, we monitored the recruitment of HIV CycT1 and Tat to the reactivating proviruses. By 4 h postactivation, there was sufficient transcribed and translated HIV RNA for newly synthesized CD8a-d2EGFP protein to appear in the cytoplasmic membrane compartments (Fig. 5 *A*–*C*).

CycT1 was found at minimal levels in quiescent T cells (Fig. 5*B*) but accumulated dramatically in the nucleus after TCR activation. The recruitment of CycT1, pSer175-CDK9, 7SK RNA, and Tat to the HIV provirus (Fig. 5 and *SI Appendix*, Fig. S19) mirrored the time course of HIV RNA synthesis (Fig. 4*B*), with an initial lag period of 3 h before peaking at 6 h, consistent with the critical role for P-TEFb in supporting HIV transcription. There was a slight decline in CycT1 association with the provirus by 24 h, which may represent a slow-down in transcription at this stage (*SI Appendix*, Fig. S19). As expected, the intranuclear distribution of CycT1 mirrored that of 7SK snRNA. The regions where CycT1 colocalized with 7SK snRNA were also indicative of the 7SK snRNP complexes used to deliver P-TEFb to cellular genes and the HIV provirus (*SI Appendix*, Fig. S20).

Similarly, there was no detectable HIV Tat expressed in the quiescent latently infected cells. Upon TCR activation, there was a rapid synthesis of Tat, which was initially detected at high levels in the cytoplasm and a fraction colocalized with proviruses located within the PNC with similar kinetics to 7SK snRNA and CycT1 (Fig. 5*C* and *SI Appendix*, Fig. S21).

Due to the shift of proviruses out of the PNC during T cell reactivation, the maximal accumulation of the 7SK snRNA, Tat, and CycT1 components was observed at the proviruses located 0.25 to 0.49 µm from nucleolus in the IEC (*SI Appendix*, Fig. S19*C*), which was also the region of lowest DNA density. Proviruses located further away from the nucleolus in the IEC had significantly lower levels of 7SK snRNA, CycT1, and Tat and higher DNA density, consistent with their delayed reactivation kinetics. The few proviruses located near the nuclear periphery had reduced levels of 7SK snRNA, CycT1, and Tat. Since they were in regions of the highest DNA density, it seems likely that these proviruses were associated with heterochromatic structures and more transcriptionally repressed (*SI Appendix*, Fig. S19*C*).

The preferential reactivation of proviruses in the PNC could also be observed in the rare cells (less than 15%) that had more than one HIV locus. For Tat, CycT1, and 7SK snRNA, only the HIV DNA found in the PNC accumulated high levels of the transcription factors after TCR stimulation (*SI Appendix*, Fig. S22).

### Proviral Dynamics After Reactivation in Latently Infected Cells from Well-Suppressed Donors.

**Fig. 6.**
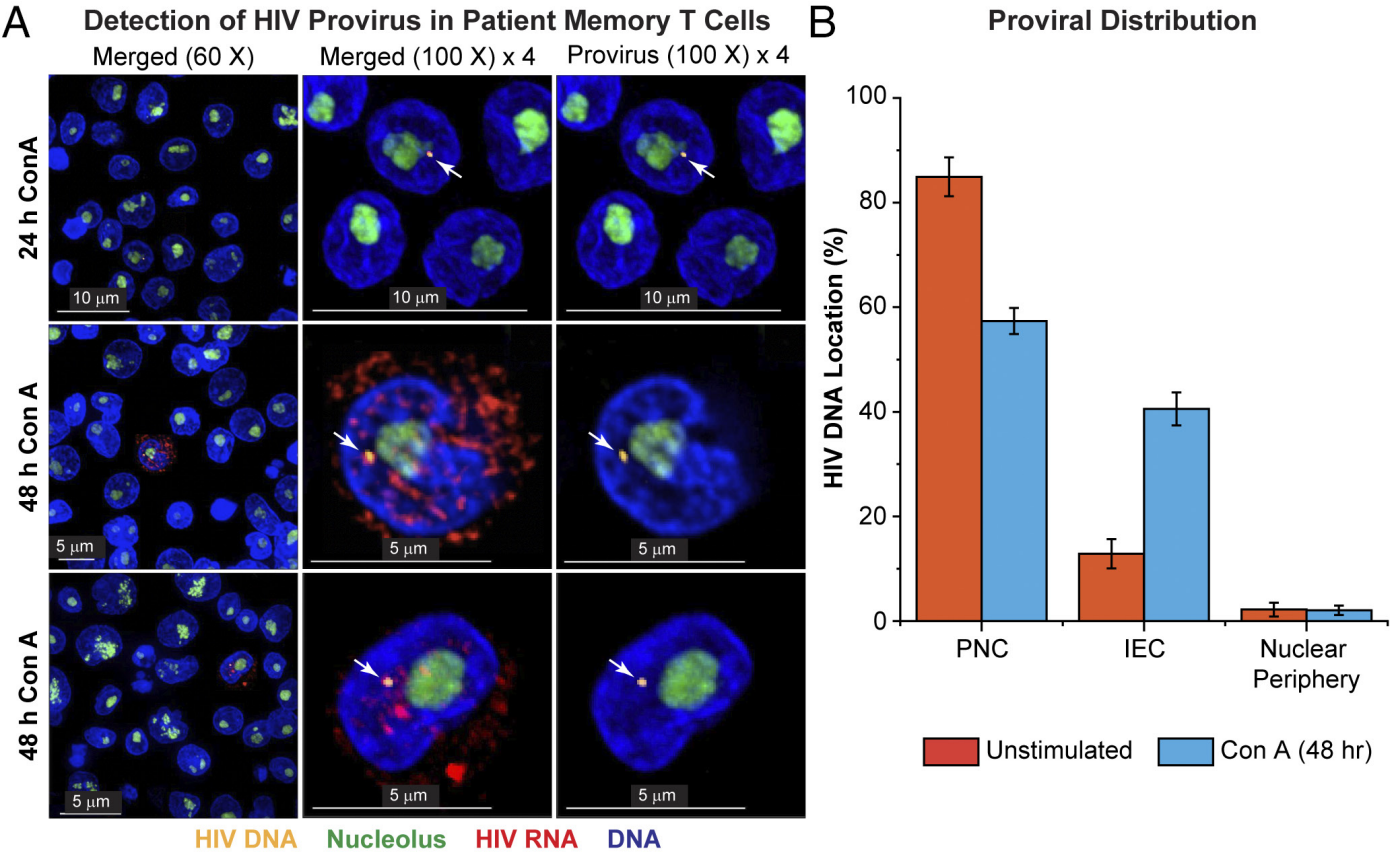
Cas-FISH detection of HIV provirus in reactivated memory CD4**^+^** T cells isolated from an HIV-infected donor. (*A*) Representative micrographs showing HIV-infected cells within a population of uninfected memory CD4+ T cells. Detected proviruses (yellow staining highlighted by white arrows) and HIV RNA transcripts (red) are colocalized in the perinucleolar and IEC. Cells were activated by treatment with 10 ng/mL Con A for 24 or 48 h. Yellow: HIV proviral DNA (Alexa Fluor 555). Red: Quasar 670-labeled HIV LTR-specific Stellaris RNA FISH probes. Green: Nucleoli stained with fluorescein-labeled 45S pre-rRNA intron-specific Stellaris probes. Blue: DNA stained using DAPI. All scale bars represent a length of 5 µm. Images were taken at 60× or 100× and enlarged threefold. (*B*) Bar graph showing the percent proviral DNA distribution within the three subnuclear zones before and after Con A stimulation. Error bars: SD.

To ensure that the results we obtained in the QUECEL Th17 model accurately reflect the behavior of latently infected memory CD4^+^ T cells isolated from well-suppressed HIV-infected donors, we performed imaging experiments on memory T cells isolated from PBMCs that were activated for 24 or 48 h with Concanavalin A (Con A) (Fig. 6). Because of the rarity of latently infected cells in the samples, transcriptionally active cells were enriched by sorting for cells with downregulated CD4 on the surface, due to activation of Nef. In a minority of the ConA-activated T cells where we detected proviruses, we were also able to detect high levels of HIV transcripts in the nucleus and the cytoplasm (Fig. 6*A*) with distributions that closely resembled the transcript distributions seen in the QUECEL model (Fig. 4*A* and *SI Appendix*, Fig. S15). In a few cells, we detected nascent HIV transcripts restricted to the proviral DNA, presumably reflecting limited transcription from a defective provirus (Fig. 6*A*).

Importantly, distance measurements of the provirus from the nucleolus in 100 infected cells obtained from patients that were untreated or stimulated with Con A showed that over 80% of the detected proviral DNA was localized in the PNC (Fig. 6*B* and *SI Appendix*, Fig. S23*A*). In addition, we observed that ~30% of proviruses shifted from the PNC to the IEC and nuclear periphery after 48 h of Con A activation, consistent with the results we obtained using the QUECEL model (*SI Appendix*, Fig. S17*A*). Finally, we were also able to detect HIV RNA transcripts that accumulated in the PNC after only 2 h of TCR stimulation (*SI Appendix*, Fig. S23*C*). This was also consistent with the kinetics of nascent RNA production seen in the QUECEL model (Fig. 4*A*).

## Discussion

### Nuclear Compartmentalization Permits Efficient Transcription.

The nucleus is structurally compartmentalized and contains distinct chromosomal regions and numerous subnuclear domains. In vivo imaging approaches have revealed that transcriptionally active genes can move within the nucleoplasm clusters, allowing them to function independently (49). This creates an orderly 3D organization for the efficient spatiotemporal functioning of genes and processing of RNA transcripts (50). As a result, transcription occurs in “factories” harboring sets of coregulated genes where the RNA polymerase and transcription factor complexes assemble (51). Following the entry into quiescence, such as during the formation of memory T cells, condensed heterochromatin imposes chromatin folding that prevents the accessibility of gene regulators (52, 53). Noncoding RNAs and proteins involved in transcription and RNA processing also form condensates that partition the nucleus and regulate gene expression (51, 54).

### Identification of HIV DNA.

To accurately locate HIV DNA and study the impact of their location within the 3D structure of the nucleus on proviral transcription and latency, we developed a Cas-FISH imaging technique (32) using tandem arrays of gRNAs that bind to the LTR. An advantage of this method is that the nuclear morphology is preserved since the DNA is not denatured. The detection of HIV DNA can be combined with immunofluorescence and in situ RNA hybridization with fluorescent probes to study the association of proviruses with the transcriptional machinery.

Using imaging alone, it is impossible to unambiguously differentiate between HIV DNA in preintegration complexes, abortive unintegrated HIV DNA, and integrated proviruses—a generic problem for the field. However, we relied on measurements of d2EGFP expression from our reporter viruses as a proxy for HIV integration, since the d2EGFP reporter viruses lack Vpr and were unable to support transcription from unintegrated HIV DNA. This was confirmed by the ability of RALT to entirely block GFP expression. Additionally, there is a strong correlation between the fraction of cells carrying integrated HIV using the Alu-ψ assay and cells that express GFP. When working with the pHR′-CD8a-d2EGFP virus, after the purification step on day 7, approximately 90% of the cells are CD8a-d2EGFP-positive and 77% had a single DNA locus showing that these cells carried integrated proviruses.

It is more challenging to distinguish the various DNA forms at earlier time points. We therefore correlated the intranuclear localization of HIV DNA with the direct visualization of d2EGFP expression in individual cells. At days 3 and 7, nearly 100% of the cells carrying DNA in the IEC or PNC express GFP. By contrast, many cells carrying DNA in the nuclear border or nuclear periphery are GFP negative, showing that these cells probably carry unintegrated DNA.

A limitation of the Cas-FISH technology is that since it requires the fixation of cells, Cas-FISH cannot be used in real-time experiments. However, by measuring the distribution of structures in large populations of cells imaged at specific times after infection or reactivation, it is possible to infer sequential and progressive structural changes in the proviral distribution and its association with cellular cofactors mediating integration and transcription. Several other recently developed methods, including the elegant ANCHOR system, permit live cell imaging of viral DNA entry into the nucleus and its dissociation from the viral capsid (55). Unlike Cas-FISH, these methods require the addition of specialized protein binding sites into the HIV genome, which precludes their application to the study of patient samples.

### Accumulation of Latent Proviruses in the PNC as Cells become Quiescent.

As T cells enter quiescence, over 75% of the integrated HIV DNA accumulates in the PNC, where the proviruses become transcriptionally silenced. During this transition, the nucleus becomes more heterochromatic (as measured by a massive increase in the histone methylation mark H3K27me3), and the DNA also becomes more compacted (as measured by imaging and scan analysis of DAPI intensity) but remains lowest in the PNC. Notably, HIV DNA does not accumulate in the PNC when integration is blocked by the integrase inhibitor RALT, suggesting that large-scale chromatin reorganization drives the relocalization of integrated proviruses.

We hypothesize that the accumulation of epigenetic marks on the provirus and the surrounding cellular DNA as the T cells become quiescent provides an essential set of signals directing HIV proviruses to the PNC. It is important to note that the HIV LTR functions as a rare bivalent promoter, and therefore, it acquires both transcriptionally active (H3K4me3) and repressive (H3K27me3) histone methylation marks (30, 56). In human pluripotent stem cells, bivalent promoters colocalize in the nuclear interior (57). Knockdown of components of histone methyltransferases used to establish bivalency (PRC1, PRC2, or TrxG complexes) disrupts bivalent gene colocalization, demonstrating that the histone modification machinery regulates higher-order chromatin organization (57).

We confirmed the importance of the perinucleolar localization of HIV proviruses for HIV latency by showing that most proviruses found in latently infected cells obtained from well-suppressed patients on ART also accumulated in the PNC.

### CPSF6 Associates with Preintegration Complexes and Supports Transcription.

The CPSF6 protein binds to the HIV viral cores (CA, p24) and helps direct HIV-1 integration into actively transcribed regions of the cellular DNA (23, 58). Early after infection, HIV DNA was found in the nuclear periphery. As the infection progressed over the next few days, the majority of the HIV DNA was detected within the nucleus, especially in euchromatic regions, where there is both an extended residence time and where integration is likely to take place (19, 21–23, 59). Previous studies using transformed cells have localized these CPSF6-containing preintegration complexes to nuclear speckles (19, 21, 48, 59), where HIV-1 integration is enhanced in active genes and local transcriptional hotspots, which are spatially clustered (60). Our results with activated primary cells are consistent with this model.

At days 1 and 3, the only nuclear HIV DNA which is associated with both CA (p24) and CPSF6 is located at the nuclear border and these cells do not express d2EGFP, indicating that these are either HIV cores or preintegration complexes. In the following days, an increasing number of HIV DNA foci are found in the nuclear periphery that are dissociated from CA (p24). Since these cells also express d2EGFP we believe these DNA foci represent integrated proviruses. By day 7, and at subsequent time points, we could unambiguously identify all cells where integration occurred since this permitted HIV transcription and the cells were d2EGFP-positive. In each of the cells where CA (p24) had dissociated from the HIV DNA, CPSF6 was still found in proximity to the proviral DNA. We hypothesize that this is not the CPSF6 that was originally bound to CA (p24), but is instead a part of the cellular cleavage factor Im (CFIm), which is essential for the general process of polyadenylation (61).

The actively transcribing proviruses were primarily found in the IEC in the effector cells and did not accumulate at specific locations near the nuclear periphery, as seen in the transformed cell lines (20, 31). The subsequent entry of proviruses into the PNC was accompanied by a progressive reduction in the ratio of HIV DNA to CPSF6. This suggests as transcription shuts down and the cells transition to a quiescent state CPSF6, and the rest of the transcription and RNA processing machinery is lost.

### Proviral Rearrangements After T Cell Activation.

After cellular activation through the TCR, there was an expansion of the nuclear and nucleolar volumes and a reformation of euchromatic regions. During this process, proviruses shifted away from the PNC and relocated back to the IEC or, less frequently, to the nuclear periphery. However, the final distribution of proviruses in the reactivated cells still differed from transformed cell lines, where transcriptionally active proviruses were located closer to the nuclear envelope (NE) than expected by chance (20, 31).

### Recruitment of Transcriptional Elongation Machinery to HIV Proviruses Following T Cell Reactivation.

The switch between HIV latency and productive transcription is tightly regulated by a feedback mechanism fueled by the viral trans-activator protein Tat, which recruits P-TEFb together with the superelongation complex (SEC) to the proviral HIV (25). The overall effect of Tat and P-TEFb is to remove blocks to negative elongation factors which contribute to RNAP II pausing [DRB sensitivity inducing factor (DSIF) and negative elongation factor (NELF)], and allow RNAP II to resume elongation and to stimulate efficient elongation and cotranscriptional processing of proviral transcripts (25).

In contrast to transformed cells, in resting T cells P-TEFb is not yet assembled (62), and CycT1 is absent due to translational blocks imposed by microRNAs (63) and the nuclear sequestration of NF90 (64). Following activation of T cells, P-TEFb is assembled in the cytoplasm and becomes incorporated into the 7SK snRNP in the nucleus, which reversibly regulates P-TEFb by inhibiting its kinase activity (27).

There are several competing hypotheses for how P-TEFb is directed to the HIV provirus, but they generally postulate stochastic activation and unimolecular interactions at the promoter. For example, several recent studies have reported that 7SK snRNP is preassociated with HIV and cellular promoters (65). Our imaging results strongly suggest that 7SK RNP complexes containing P-TEFb and Tat accumulate in phase-separated nuclear substructures that coalesce around the actively transcribing provirus. 7SK snRNA, Tat, and CycT1 that colocalize with HIV proviral DNA could be detected as early as 2 h after T cell activation through the TCR—a time when our imaging also showed the emergence of HIV mRNA transcripts emanating from the proviruses in the PNC. Thus, the PNC appears to be a “hotspot” for assembling the proviral transcription machinery during the emergence of HIV from latency. Since these nuclear condensates contain very high concentrations of P-TEFb and Tat, they provide a robust kinetic advantage for P-TEFb interactions with the transcriptional machinery and offer a remarkable example of how nuclear compartmentalization is used to regulate HIV transcription.

## Materials and Methods

### Antibodies.

Antibody sources are given in *SI Appendix*, Table S1.

### QUECEL Model of HIV Latency.

The QUECEL method used to generate polarized infected Th17 cells has been previously described (11, 35).

### Enrichment for HIV-Infected Resting Memory CD4 T Cells from Well-Suppressed Donors.

Memory CD4 T cells from well-suppressed deidentified participants were isolated by negative selection from PBMCs (Miltenyi), incubated overnight in growth media (IMDM + 10% FBS) and stimulated overnight with 10 μg/mL Con A. The remaining CD4high T cells were then depleted using a CD4 T cell positive selection kit (StemCell). The unbound cells (enriched CD4^−^, HIV^+^ cells) were cultured overnight in growth media (IMDM + 10% FBS), concentrated by centrifugation, and seeded on poly-L-lysine coated coverslips and fixed using 4% formaldehyde for imaging.

### Endonuclease-Deficient Cas9 (dCas9) Construct and Purification.

The endonuclease deficient Cas9 (dCas9) expressing plasmid NM-01 (gift of Gerald Cagney; Addgene plasmid #69799) was transformed into *Escherichia coli* BL21 (DE3) pLysS cells and induced with 1 mM IPTG at 25 °C overnight. Cas9 was purified by immobilized metal affinity chromatography (IMAC) with Ni-NTA agarose (Qiagen, 30210) and by affinity chromatography using amylose resin (NEB, E8021S).

#### HIV DNA Cas-FISH.

Six (6) optimized DNA-tagging sequences were designed to target the U5 region of the HXB2 reference sequence LTR (*SI Appendix*, Table S1). Complex formation and hybridization methods are given in the legends of *SI Appendix*, Tables S1 and S2.

### RNA FISH and Immunofluorescence (IF).

Stellaris® RNA FISH probes that targeted the 5′ LTR of the HXB2 reference genome (66) were designed using Biosearch Technologies online software (Biosearch Technologies, Novato, CA) (*SI Appendix*, Table S3). 7SK snRNA and nucleolar probes (18 to 22 nucleotides) were custom designed from the proposed secondary structure (67) and the intronic regions of the 45S pre-rRNA (45S) transcript, respectively (*SI Appendix*, Table S3). RNA FISH methods are given in the legend to *SI Appendix*, Table S3.

### Microscopy and Image Processing.

High-resolution multiple section 2D pixel images were captured with a DeltaVision epifluorescence microscope in *z*-series (Applied Precision Imaging, GE Healthcare) using 100× or 60× magnification objectives (Olympus). Where necessary, auxiliary magnification (extra 1×, 1.6×, and using Kohler and critical illumination) and DeltaVision immersion oil with a refractive index of 1.518 Nε were used. Images were rendered to 3D projections using the SoftWoRx 7.0.0 software package (Applied Precision, Inc., Issaquah, WA).

### Quantitative Image Analysis.

Scatter fluorescence was digitally eliminated using the DeltaVision SoftWoRx 7.0.0 deconvolution program (Applied precision), and image surfaces were rendered using Imaris software (Bitplane, South Windsor, CT). Fluorescence signal intensities were quantified using line scanning, with the lower threshold set by unstimulated CD4+ T cells. SoftWoRx 7.0.0 and Imaris software were used to classify compartments as HIV DNA containments in the 3D reconstructed images.

## Supplementary Material

Appendix 01 (PDF)

## Data Availability

All study data are included in the article and/or *SI Appendix*.
