# Supplementary material for: Structural rearrangements in the nucleus localize latent HIV proviruses to a perinucleolar compartment supportive of reactivation
Source: Proc Natl Acad Sci U S A. 2024 Apr 26;121(18):e2202003121. doi: 10.1073/pnas.2202003121 (PMC11067448; doi:10.1073/pnas.2202003121)
Supplement: Supplementary file 1 — Appendix 01 (PDF) [file pnas.2202003121.sapp.pdf]

PNAS
www.pnas.org

Supplementary Information for:

Structural rearrangements in the nucleus localize latent HIV proviruses to a perinucleolar compartment supportive of reactivation

Fredrick Kizito, Kien Nguyen, Uri Mbonye, Meenakshi Shukla, Benjamin Luttge, Mary Ann Checkley, Anna Agaponova, Konstantin Leskov and Jonathan Karn*

Department of Molecular Biology and Microbiology, School of Medicine, Case Western Reserve University, 10900 Euclid Ave, Cleveland, Ohio 44106, USA.

* Corresponding author Jonathan Karn
Email: jonathan.karn@case.edu (JK)
Jonathan Karn ORCID identifier: 0000-0002-2900-094X

This PDF file includes:
Figures S1 to S23
Tables S1 to S4

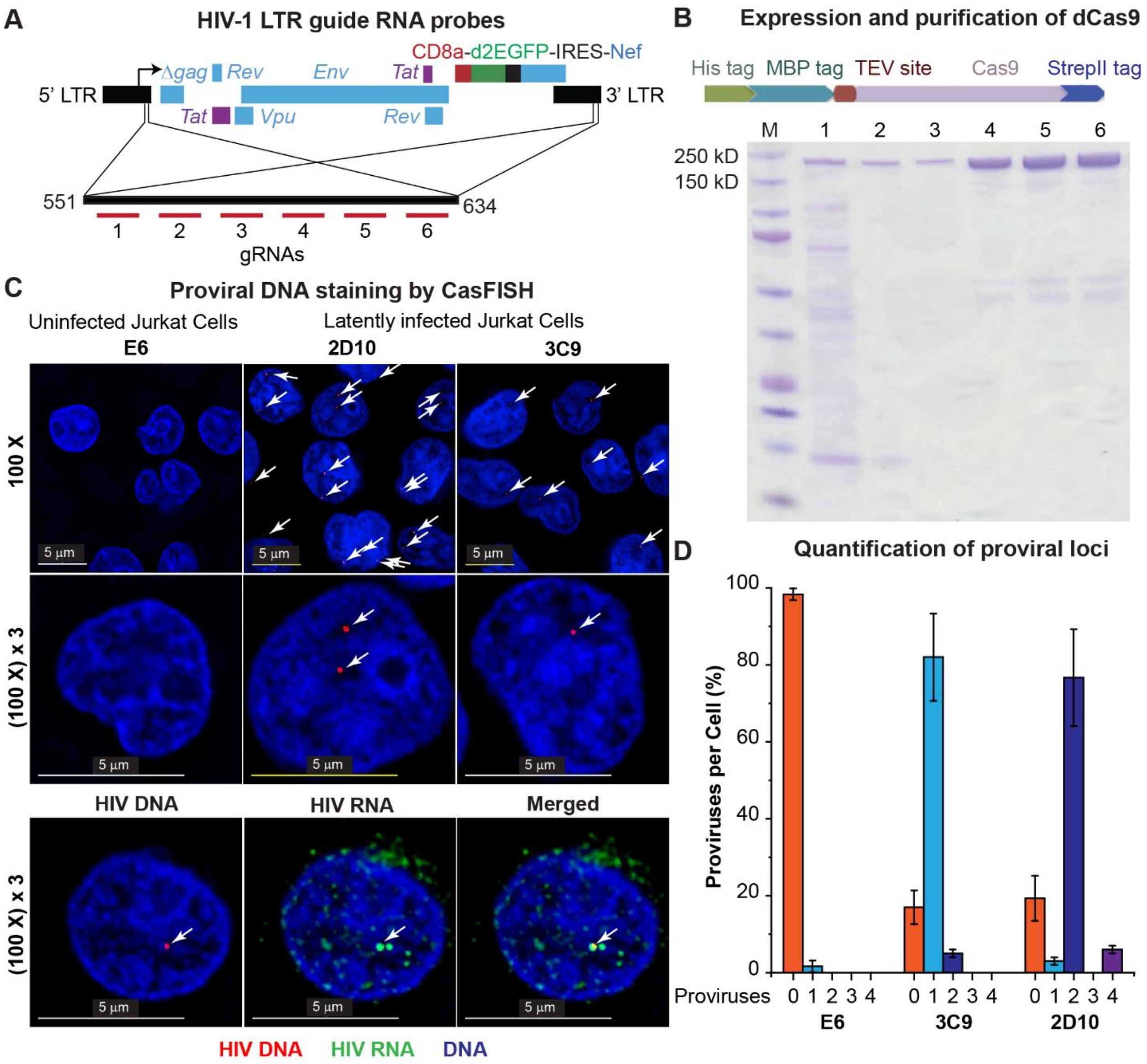

A
HIV-1 LTR guide RNA probes
CD8a-d2EGFP-IRES-Nef
Δgag Rev Env Tat
5' LTR
3' LTR
Tat Vpu Rev
551
634
1 2 3 4 5 6
gRNAs
B
Expression and purification of dCas9
His tag MBP tag TEV site Cas9 StrepII tag
M 1 2 3 4 5 6
250 kD
150 kD
C
Proviral DNA staining by CasFISH
Uninfected Jurkat Cells
Latently infected Jurkat Cells
E6
2D10
3C9
100 X
(100 X) x 3
5 μm
HIV DNA
HIV RNA
Merged
HIV DNA HIV RNA DNA
D
Quantification of proviral loci
Proviruses per Cell (%)
100
80
60
40
20
0
Proviruses 0 1 2 3 4 0 1 2 3 4 0 1 2 3 4
E6
3C9
2D10

Fig. S1. CasFISH design and detection of HIV proviruses. (A) Diagram showing the position of the non-overlapping HIV-LTR gRNA sequences. Six tandem HIV-gRNA sequences targeting the U5 region between nucleotides 551 and 634 and the corresponding U3 region of the viral LTR were designed to allow specific binding interactions at the proviral locus. (B) Purification of recombinant inactive Cas9 (dCas9) from *E. Coli* by amylose affinity chromatography and analysis by SDS-PAGE. M: Molecular weight standards, Lanes 2-4: Wash fractions, Lanes 5-7: Eluted fractions containing purified dCas9. (C) *Top and middle panels*, Detection of the proviral DNA in control Jurkat E6 cells, 3C9 and 2D10 cells stably harboring latent HIV-1 proviruses by CasFISH (Red: Alexa Fluor 647-labeled secondary antibody to dCas9). *Bottom panels*, HIV DNA CasFISH performed with RNA FISH using Fluorescein-labeled Stellaris probes to the HIV U5 RNA following TNF-α activation using 3C9 cells. All images were taken using a high-resolution DeltaVision deconvolution microscope at 100X magnification. Images in the middle and bottom panels have been enlarged 3-fold to show single representative cells. Scale bars represent a length of 5 µm. White arrows indicate the position of the proviruses. (D) A graph showing the average counts of quantified HIV DNA puncta obtained from three different image data sets containing 100 cells randomly selected from E6, 3C9 and 2D10 Jurkat clonal populations. Error bars denote standard deviation.

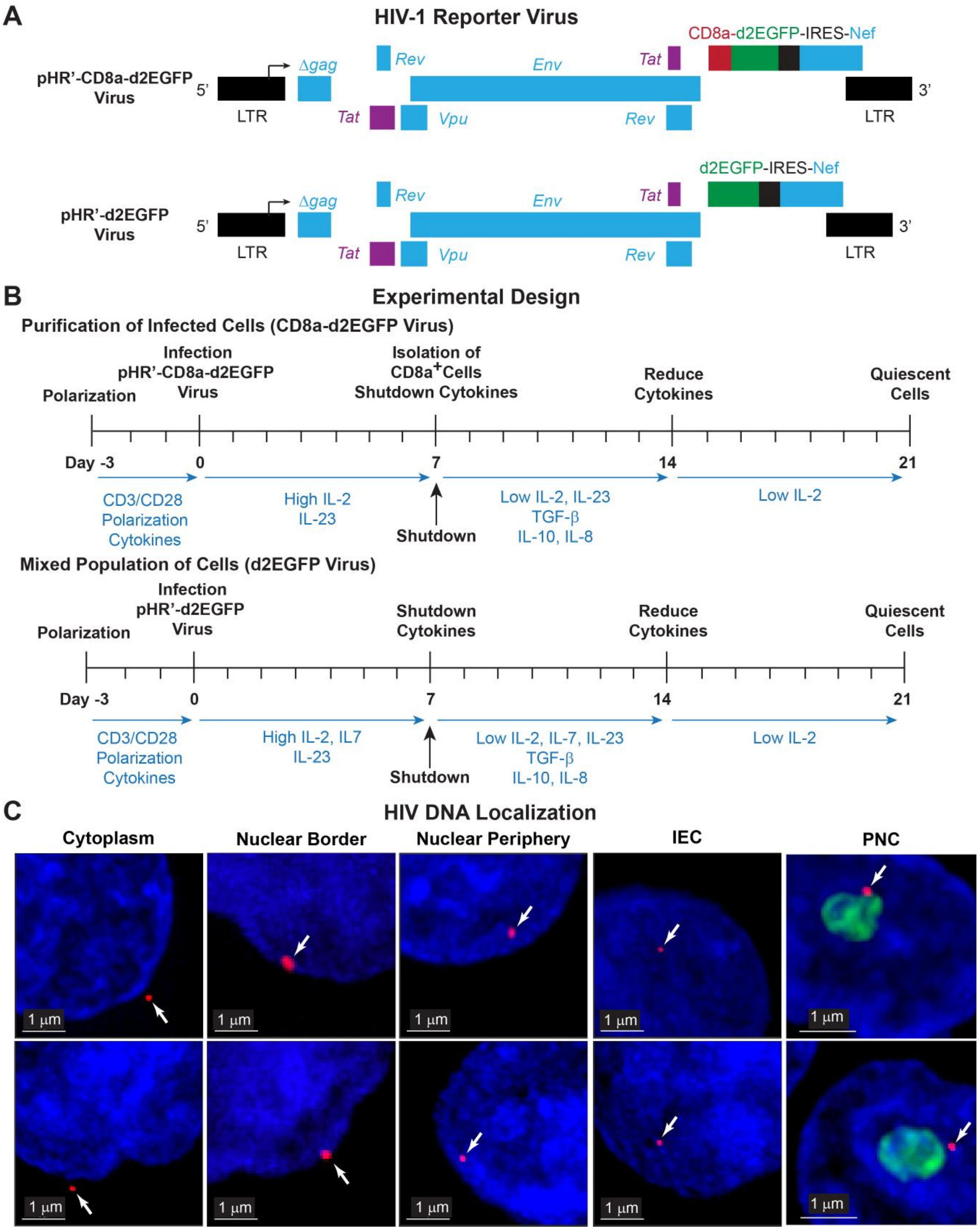
A
HIV-1 Reporter Virus
pHR'-CD8a-d2EGFP Virus
5'
LTR
Δgag
Rev
Env
Tat
CD8a-d2EGFP-IRES-Nef
3'
Tat
Vpu
Rev
pHR'-d2EGFP Virus
d2EGFP-IRES-Nef
B
Experimental Design
Purification of Infected Cells (CD8a-d2EGFP Virus)
Polarization
Infection pHR'-CD8a-d2EGFP Virus
Isolation of CD8a+Cells Shutdown Cytokines
Reduce Cytokines
Quiescent Cells
Day -3
0
7
14
21
CD3/CD28 Polarization Cytokines
High IL-2 IL-23
Shutdown
Low IL-2, IL-23 TGF-β IL-10, IL-8
Low IL-2
Mixed Population of Cells (d2EGFP Virus)
Polarization
Infection pHR'-d2EGFP Virus
Shutdown Cytokines
Reduce Cytokines
Quiescent Cells
Day -3
0
7
14
21
CD3/CD28 Polarization Cytokines
High IL-2, IL7 IL-23
Shutdown
Low IL-2, IL-7, IL-23 TGF-β IL-10, IL-8
Low IL-2
C
HIV DNA Localization
Cytoplasm
Nuclear Border
Nuclear Periphery
IEC
PNC
1 μm

Fig. S2. Experimental design and examples of HIV DNA localization. (A) Gene maps of the pHR'-CD8a-d2EGFP reporter virus (1) and the pHR'-d2EGFP reporter virus (2, 3). Both vectors are based on HIV-1 NL4-3 and are isogenic apart from the reporter. (B) Experimental design for QUECEL experiments. The top diagram depicts the protocol for working with the pHR'-CD8a-GFP virus. Typically, the CD8a-d2GFP-expressing cells are purified at Day 7 by magnetic bead separation and placed in a cocktail of cytokines containing TGF-β that promotes entry into quiescence (1). The bottom diagram depicts the protocol for working with the pHR'-d2EGFP reporter virus, which involves working with mixed populations of infected and uninfected cells. This modified protocol includes IL-7, which enhances cell viability. IL-7 is removed during the last week of the cellular shutdown. (C) Representative examples of HIV DNA localized to different intranuclear regions. Detection of the proviral DNA HIV-1 proviruses by CasFISH (Red: Alexa Fluor 647-labeled secondary antibody to dCas9). From left to right: Cytoplasm (Day 1), Nuclear Border (Day 3), Nuclear Periphery (Day 3), IEC (Day 7), and PNC (Day 21). Nucleoli stained with Fluorescein-labeled 45S pre-rRNA intron-specific Stellaris probes are shown in green. Blue: DNA stained using DAPI.

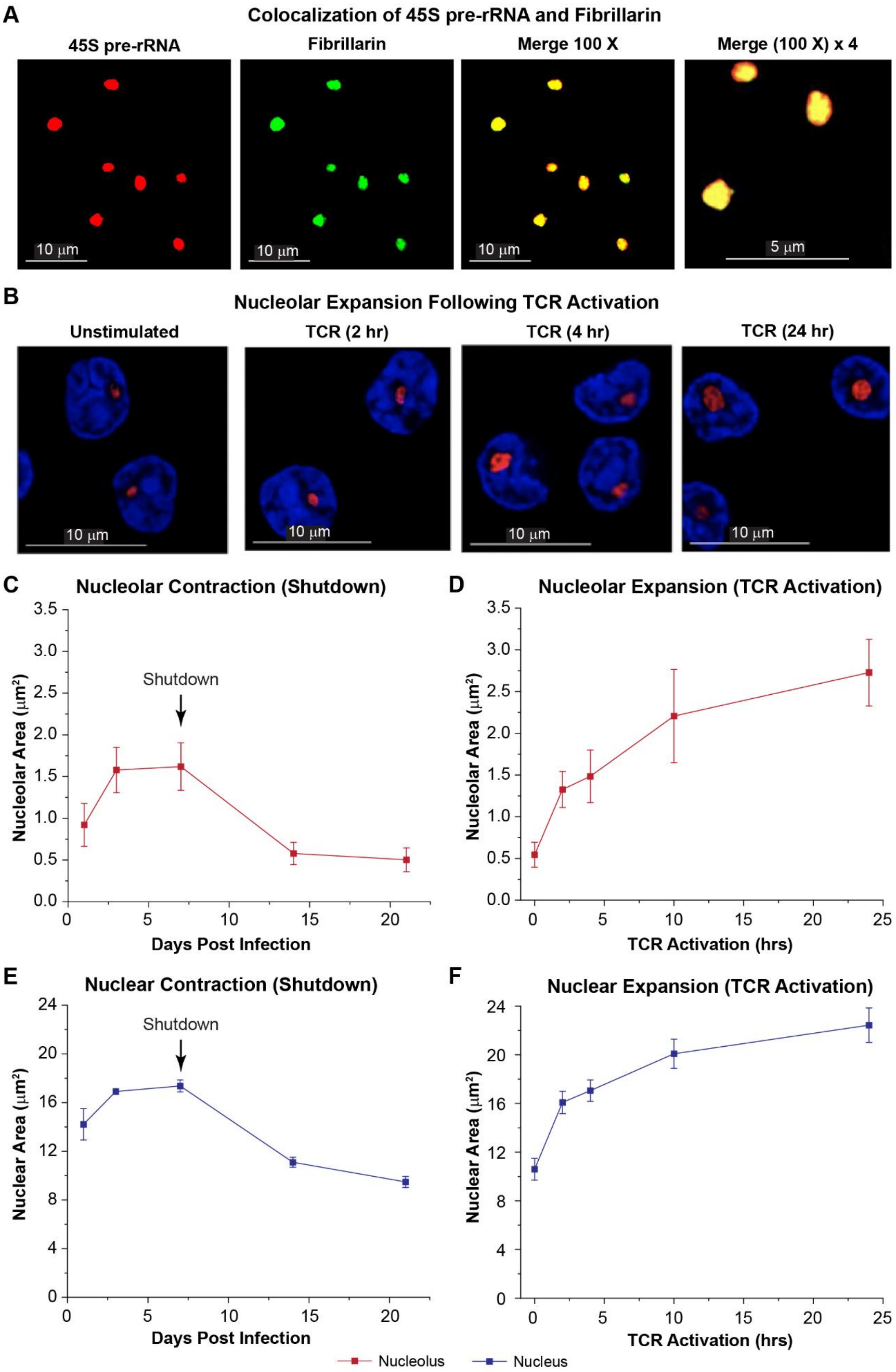
A
Colocalization of 45S pre-rRNA and Fibrillarin
45S pre-rRNA
Fibrillarin
Merge 100 X
Merge (100 X) x 4
10 μm
5 μm
B
Nucleolar Expansion Following TCR Activation
Unstimulated
TCR (2 hr)
TCR (4 hr)
TCR (24 hr)
C
Nucleolar Contraction (Shutdown)
Shutdown
Nucleolar Area (μm²)
Days Post Infection
D
Nucleolar Expansion (TCR Activation)
TCR Activation (hrs)
E
Nuclear Contraction (Shutdown)
Nuclear Area (μm²)
F
Nuclear Expansion (TCR Activation)
Nucleolus
Nucleus

Fig. S3. Expansion and contraction of the nucleus and nucleoli in quiescent and reactivated primary T cells. (A) Colocalization of Quasar670-labeled 45S pre-rRNA intron-specific Stellaris probes (red) with immunofluorescence detection of Alexa Fluor 488-labeled secondary antibody to the nucleolar structural protein fibrillarin (green). (B) Representative images depicting the change in nuclear area (blue) and nucleolar area (red) after memory T-cell activation by TCR stimulation. Also notable are the reductions in the intranuclear DNA density, as measured by DAPI (blue) staining after reactivation. (C) Time course showing contraction of the nucleolus as cells enter quiescence during the QUECEL protocol. (D) Time course showing nucleolar expansion during 24 hours after T-cell activation by CD3-CD28 Dynabeads. (E) Changes in nuclear area for the same cells shown in panel C. (F) Changes in nuclear area for the same cells shown in panel D. Nuclear and nucleolar areas were calculated from the measured length and widths of 300 cells. Error bars: Standard Deviation.

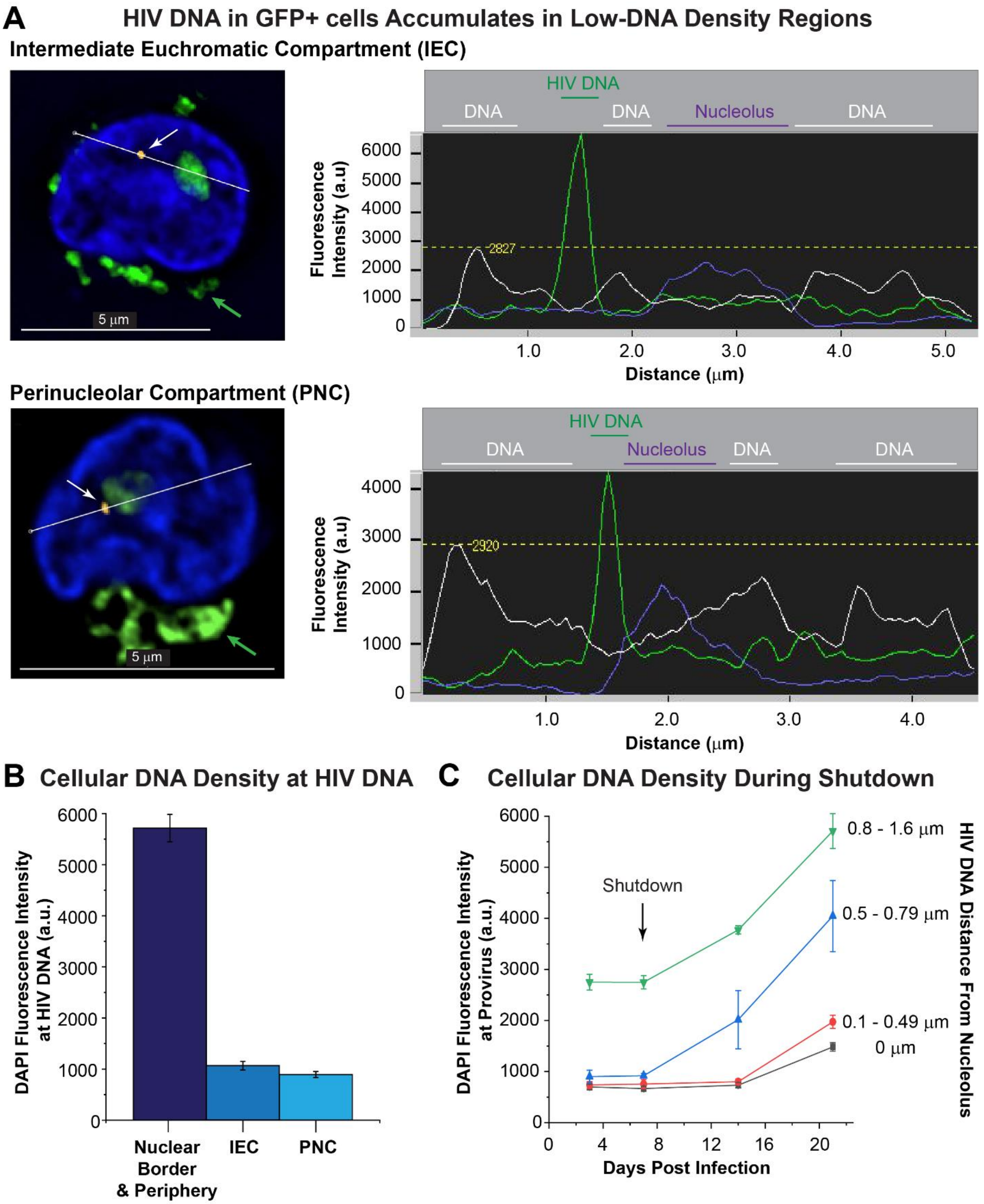
A
HIV DNA in GFP+ cells Accumulates in Low-DNA Density Regions
Intermediate Euchromatic Compartment (IEC)
5 μm
HIV DNA
DNA
DNA
Nucleolus
DNA
Fluorescence Intensity (a.u)
0
1000
2000
3000
4000
5000
6000
2827
1.0
2.0
3.0
4.0
5.0
Distance (μm)
Perinucleolar Compartment (PNC)
5 μm
HIV DNA
DNA
Nucleolus
DNA
DNA
Fluorescence Intensity (a.u)
0
1000
2000
3000
4000
2920
1.0
2.0
3.0
4.0
Distance (μm)
B
Cellular DNA Density at HIV DNA
DAPI Fluorescence Intensity at HIV DNA (a.u.)
0
1000
2000
3000
4000
5000
6000
Nuclear Border & Periphery
IEC
PNC
C
Cellular DNA Density During Shutdown
DAPI Fluorescence Intensity at Provirus (a.u.)
0
1000
2000
3000
4000
5000
6000
Shutdown
0
4
8
12
16
20
Days Post Infection
0.8 - 1.6 μm
0.5 - 0.79 μm
0.1 - 0.49 μm
0 μm
HIV DNA Distance From Nucleolus

Fig S4. Accumulation of transcribed HIV DNA in regions of low DNA density. (A) Line scans of representative HIV DNA found in the Intermediate Euchromatic Compartment (IEC) (at Day 7) or Perinucleolar Compartment (PNC) (at Day 21). The dotted line on the scans is the maximal detected DNA fluorescent intensity as measured by DAPI staining. White arrows show the location of the HIV DNA (Yellow: Alexa Fluor 555-labeled secondary antibody to dCas9). Nucleoli stained with Fluorescein-labeled 45S pre-rRNA intron-specific Stellaris probes are shown in green inside the DNA. Green arrows outside the DNA show GFP in the cytoplasm of the cells. (B) Average DAPI fluorescent intensity at HIV DNA located in various nuclear sub-compartments. (C) Average DAPI fluorescent intensity at HIV DNA located at various distances from the nucleolus.

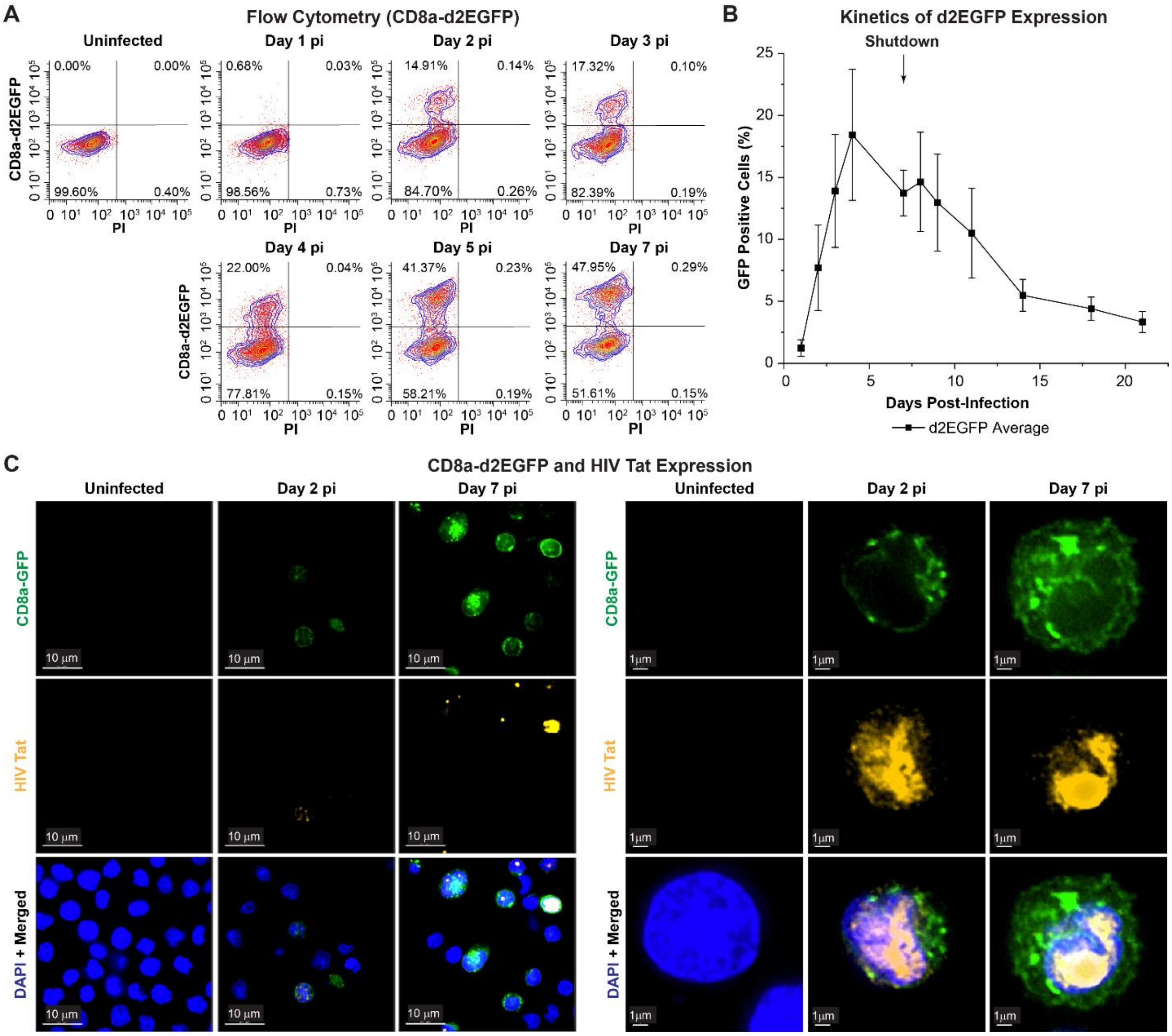
A
Flow Cytometry (CD8a-d2EGFP)
Uninfected
Day 1 pi
Day 2 pi
Day 3 pi
Day 4 pi
Day 5 pi
Day 7 pi
CD8a-d2EGFP
PI
0.00%
0.00%
99.60%
0.40%
0.68%
0.03%
98.56%
0.73%
14.91%
0.14%
84.70%
0.26%
17.32%
0.10%
82.39%
0.19%
22.00%
0.04%
77.81%
0.15%
41.37%
0.23%
58.21%
0.19%
47.95%
0.29%
51.61%
0.15%
B
Kinetics of d2EGFP Expression
Shutdown
GFP Positive Cells (%)
Days Post-Infection
d2EGFP Average
C
CD8a-d2EGFP and HIV Tat Expression
Uninfected
Day 2 pi
Day 7 pi
CD8a-GFP
HIV Tat
DAPI + Merged
10 µm
1µm

Fig. S5. GFP and Tat Expression before entry of cells into quiescence. (A) Flow cytometry detection of CD8a-d2GFP expression in cells infected by the pHR'-CD8a-d2EGFP virus at Days 1 to 7. (B) Kinetics of d2EGFP expression in cells infected by pHR'-d2EGFP virus between Days 1 to 21 in the QUECEL protocol. d2EGFP has a shorter half-life than CD8a-d2EGFP and therefore does not persist in the latently infected cells. Data is the average of 3 independent experiments. Error bars: Standard Deviation. (C) CD8a-d2EGFP and HIV Tat expression in cells after acute infection by the pHR'-CD8a-d2EGFP virus on Days 2 and 7. Left panels: Broad-field images were captured with a 100X objective, and scale bars represent a length of 10 μm. Right panels: Single-cell images with scale bars representing a length of 1 μm.

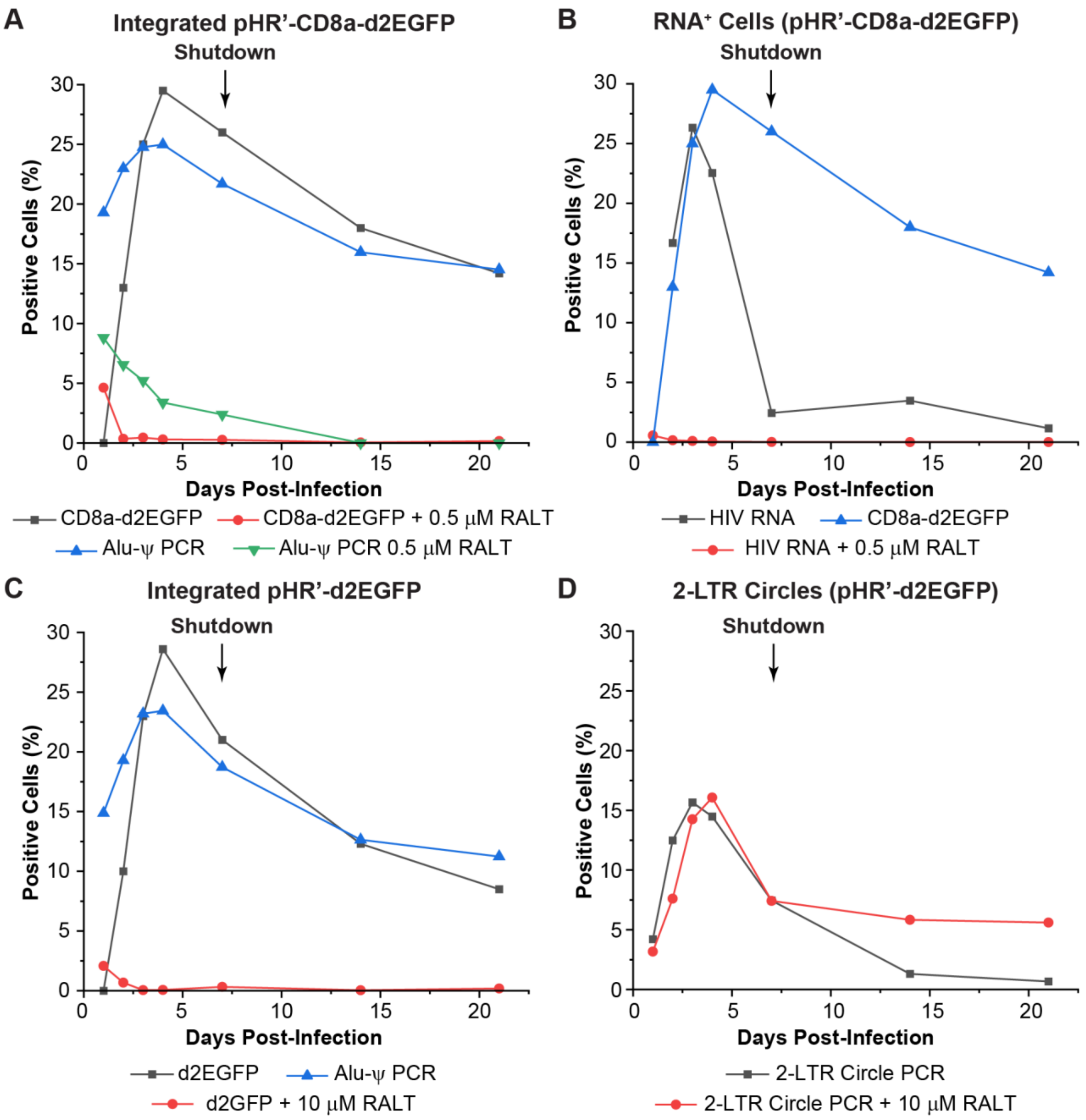
A
Integrated pHR'-CD8a-d2EGFP
Shutdown
Positive Cells (%)
Days Post-Infection
CD8a-d2EGFP
CD8a-d2EGFP + 0.5 μM RALT
Alu-ψ PCR
Alu-ψ PCR 0.5 μM RALT
B
RNA+ Cells (pHR'-CD8a-d2EGFP)
Shutdown
Positive Cells (%)
Days Post-Infection
HIV RNA
CD8a-d2EGFP
HIV RNA + 0.5 μM RALT
C
Integrated pHR'-d2EGFP
Shutdown
Positive Cells (%)
Days Post-Infection
d2EGFP
Alu-ψ PCR
d2GFP + 10 μM RALT
D
2-LTR Circles (pHR'-d2EGFP)
Shutdown
Positive Cells (%)
Days Post-Infection
2-LTR Circle PCR
2-LTR Circle PCR + 10 μM RALT

Fig. S6. Time course showing the detection of HIV integration and 2-LTR circles by PCR. (A) Integration of the pHR'-CD8a-d2EGFP virus was measured by an Alu-ψ PCR assay. The fraction of cells carrying integrated proviruses at each time point was estimated by comparison to PCR standards (assuming one integrant per cell). These values were compared to the fraction of cells expressing CD8a-d2EGFP as measured by flow cytometry. Note that integrated proviruses were detected before the onset of CD8a-dGFP-expression, presumably due to delays in the accumulation of the protein. The estimates obtained by both methods converge on Day 3. The blocking of integration by 0.5 μM RALT was measured by PCR and CD8a-d2EGFP expression. (B) RNA expression in pHR'-CD8-d2EGFP infected cells. RNA was detected by a nested PCR assay measuring the total spliced mRNAs for the same samples as in Panel A, and the GFP profile is repeated as a reference. RNA expression fell rapidly after Day 4, as the cells spontaneously entered quiescence. RNA expression was also effectively blocked by 0.5 μM RALT. (C) Integration of the pHR'-d2EGFP virus was measured by an Alu-ψ PCR assay. Note that the data for this biological replicate closely matches the data shown in Panel A. (D) Fraction of cells carrying 2-LTR circles as determined by PCR amplification of the 2-LTR circle junction sequence. Only 0.67% of cells retained 2-LTR circles on Day 21. By contrast, after treatment with 10 μM RALT, 5.6% of the cells retained 2-LTR circles on Day 21.

HIV-1 Integration Assay. Integration was quantified based on the Alu-Gag PCR assay (4) with modifications to accommodate the lack of HIV gag in the vectors used in this study. Cellular DNA was isolated from HIV-transduced primary Th17 cells using AllPrep DNA/RNA Mini Kit (Qiagen) and quantified with a Qubit ds DNA HS Assay Kit and a Qubit 3.0 Fluorometer. The first round of PCR included 1 µM Alu Fwd primer BL434F: 5' GCCTCCCAAAGTGCTGGGATTACAG, 1 µM HIV-1 psi Rvs primer BL383R: 5' GCACCCATCTCTCTCCTTCTAGC, 30 ng of cellular DNA, and BlastTaq qPCR Master Mix (Applied Biological Materials Inc.) in 20 µl reactions loaded into a 384-well PCR plate. Samples

were then amplified in a Techne thermal cycler with the following conditions: 95° C for 3 min, followed by 20 cycles of 95° C for 15 sec, 50° C for 15 sec, and 72° C for 2.5 min, before finishing with a final extension at 72° C for 10 min and holding at 4° C. Integrated HIV DNA was then quantified by nested qPCR in HardShell® 384-well clear shell/white well PCR plates (Bio-Rad) using a Bio-Rad CFX Opus Real-Time PCR system. Each well contained 0.5 microliter of first round PCR product diluted in 15 µl reactions containing 900 nM of HIV LTR Fwd primer BL418F: 5' AAAATCTCTAGCAGTGGCGCC, 900 nM of HIV psi Rvs primer BL421R: 5' CTCGCCTCTTGCCGTGYGC, 250 nM of TaqMan MGB HIV psi probe BL273-TM: 5'-FAM-CAGCAAGCCGAGTCCTGCGTCGAG, and BlasTaq™ Probe qPCR Master Mix (Applied Biological Materials Inc.). Plates were incubated at 95° C for 30 sec, then 40 cycles of 95° C for 5 sec and 60° C for 15 sec followed by a plate read and Cq values were analyzed with CFX Maestro 2.3 software (BioRad). To estimate % HIV-integrated cells, samples were compared with randomly integrated cellular DNA standards created by a limiting serial dilution of acutely infected $CD4^+$ T cells, which were enriched for GFP^+ cells by fluorescent cell sorting (Wolf Cell Sorter) and diluted in a 2-fold series with HIV-negative PBMCs to a range of 512 to less than one HIV+ cell per million. Cellular DNA was then isolated using an AllPrep Mini Kit (Qiagen). Since samples in the QUECEL model contained a much higher percentage of HIV+ cells than these limiting dilution standards, an additional reference of randomly integrated DNA from cells acutely infected with HIV-GFP that was quantified as approximately 20% and 5% GFP+ by flow cytometry were also used to improve quantitation of integration in this range.

2-LTR Circle Assay. Unintegrated 2-LTR circles of HIV vectors in transduced primary cells were quantified by digital PCR, based on methods previously described using Real-Time PCR (5), with some modifications to accommodate mismatches in NL4-3-based vectors compared to the primer sequences cited and potential mismatches in subtype B primary isolates. Digital PCR samples included 900 nM Forward primer BL388F: 5'

ARCTRGGGAACCCACTGCTTAAG, 900 nM Reverse primer BL389R: 5' TCCACAGATCAAGGATATCTTGTC, 250 nM VIC-labeled TaqMan MGB HIV 3'LTR Probe BL390-TM: 5' CACACTACTTRAAGCACTCAAGGCAAGC, and Absolute Q™ DNA Digital PCR Master Mix. 9 µl of each sample was loaded into MAP16 plates, overlain with 15 µl immersion oil, and run in a QuantStudio Absolute Q Digital PCR System (Applied Biosystems) with the following conditions: 96 °C for 10 min, followed by 40 cycles of 96 °C for 5 sec and 60 °C for 15 sec. Samples were analyzed with QuantStudio Absolute Q Digital PCR System Software and compared with uninfected cell DNA controls to establish baseline fluorescence. Specificity for 2-LTR circles was confirmed by testing a positive control plasmid containing a synthetic 2-LTR circle junction (kindly provided by Jacek Skowronski, CWRU).

Spliced HIV mRNA Assay. Total cellular RNA was isolated (AllPrep Mini DNA/RNA Kit) and quantified by fluorometry (Qubit HS RNA Assay Kit). 20 ng total RNA was diluted to 1 ng RNA/µl in 20 µl per well of HardShell® 384-well clear shell/white well PCR plates (Bio-Rad) containing HIV-1 5' LTR Fwd primer (BL271F), HIV-1 tat/rev Rvs primer (BL305R), TaqMan MGB HIV psi probe (BL273-TM), and TaqMan™ Fast Virus 1-Step Master Mix (AppliedBiosystems). Samples were run in parallel with RNA from limiting dilutions of HIV-infected cells that were also used for the HIV-1 integration assay. Reactions were performed in a CFX Opus 384 Real-Time PCR System (Bio-Rad) at 50 °C for 15 minutes, 95 °C for 20 seconds, and 40 cycles of 95 °C for 3 sec and 55 °C for 30 seconds followed by a plate read. Cq values were analyzed with CFX Maestro 2.3 software (BioRad).

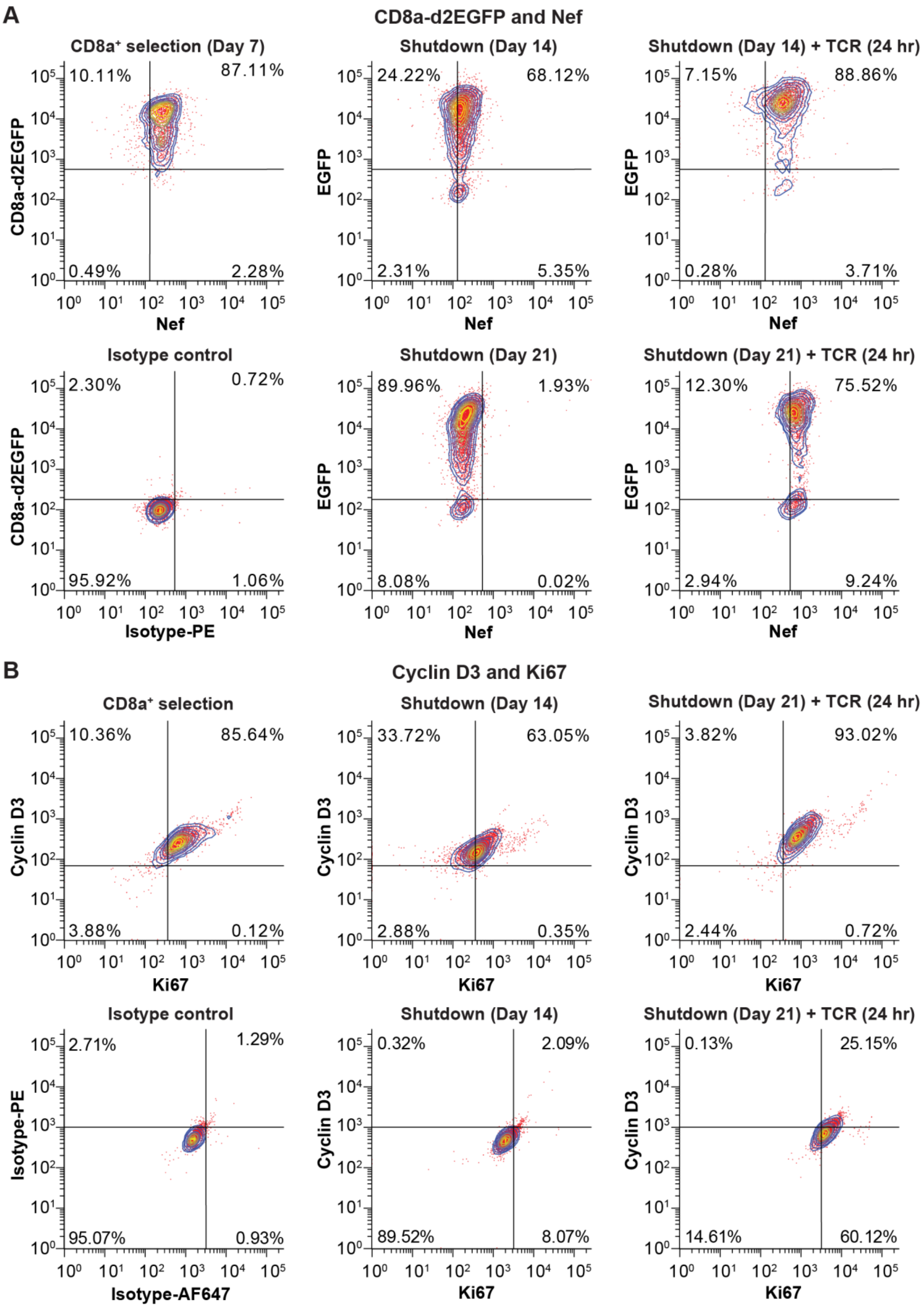
A
CD8a-d2EGFP and Nef
CD8a⁺ selection (Day 7)
10.11%
87.11%
0.49%
2.28%
CD8a-d2EGFP
Nef
Shutdown (Day 14)
24.22%
68.12%
2.31%
5.35%
EGFP
Nef
Shutdown (Day 14) + TCR (24 hr)
7.15%
88.86%
0.28%
3.71%
EGFP
Nef
Isotype control
2.30%
0.72%
95.92%
1.06%
CD8a-d2EGFP
Isotype-PE
Shutdown (Day 21)
89.96%
1.93%
8.08%
0.02%
EGFP
Nef
Shutdown (Day 21) + TCR (24 hr)
12.30%
75.52%
2.94%
9.24%
EGFP
Nef
B
Cyclin D3 and Ki67
CD8a⁺ selection
10.36%
85.64%
3.88%
0.12%
Cyclin D3
Ki67
Shutdown (Day 14)
33.72%
63.05%
2.88%
0.35%
Cyclin D3
Ki67
Shutdown (Day 21) + TCR (24 hr)
3.82%
93.02%
2.44%
0.72%
Cyclin D3
Ki67
Isotype control
2.71%
1.29%
95.07%
0.93%
Isotype-PE
Isotype-AF647
Shutdown (Day 14)
0.32%
2.09%
89.52%
8.07%
Cyclin D3
Ki67
Shutdown (Day 21) + TCR (24 hr)
0.13%
25.15%
14.61%
60.12%
Cyclin D3
Ki67

Fig. S7. Analysis of the cell proliferation and HIV expression markers by flow cytometry. (A) Cells were infected with the pHR'-CD8a-GFP reporter virus and the expression of CD8a-d2EGFP, and HIV Nef was measured by flow cytometry. Both markers were high on Day 7, after CD8 selection, and on Day 14 (1 week after the induction of quiescence), demonstrating active proviral expression. By Day 21, as the cells progressed into the second week post-shutdown, there was a significant reduction in HIV-Nef expression, demonstrating proviral silencing. By contrast, the membrane-bound CD8a-GFP persisted at a low level in cells after proviral shutdown and provided a marker for infected cells. (B) Cyclin D3 and Ki67 expression in cells transitioning from the effector to a quiescence phenotype using the QUECEL protocol. The reduction in Cyclin D3 and Ki67 levels during shutdown confirmed the cells' entry into quiescence. T-cell reactivation through the TCR induced the expression of Cyclin D3 and Ki67.

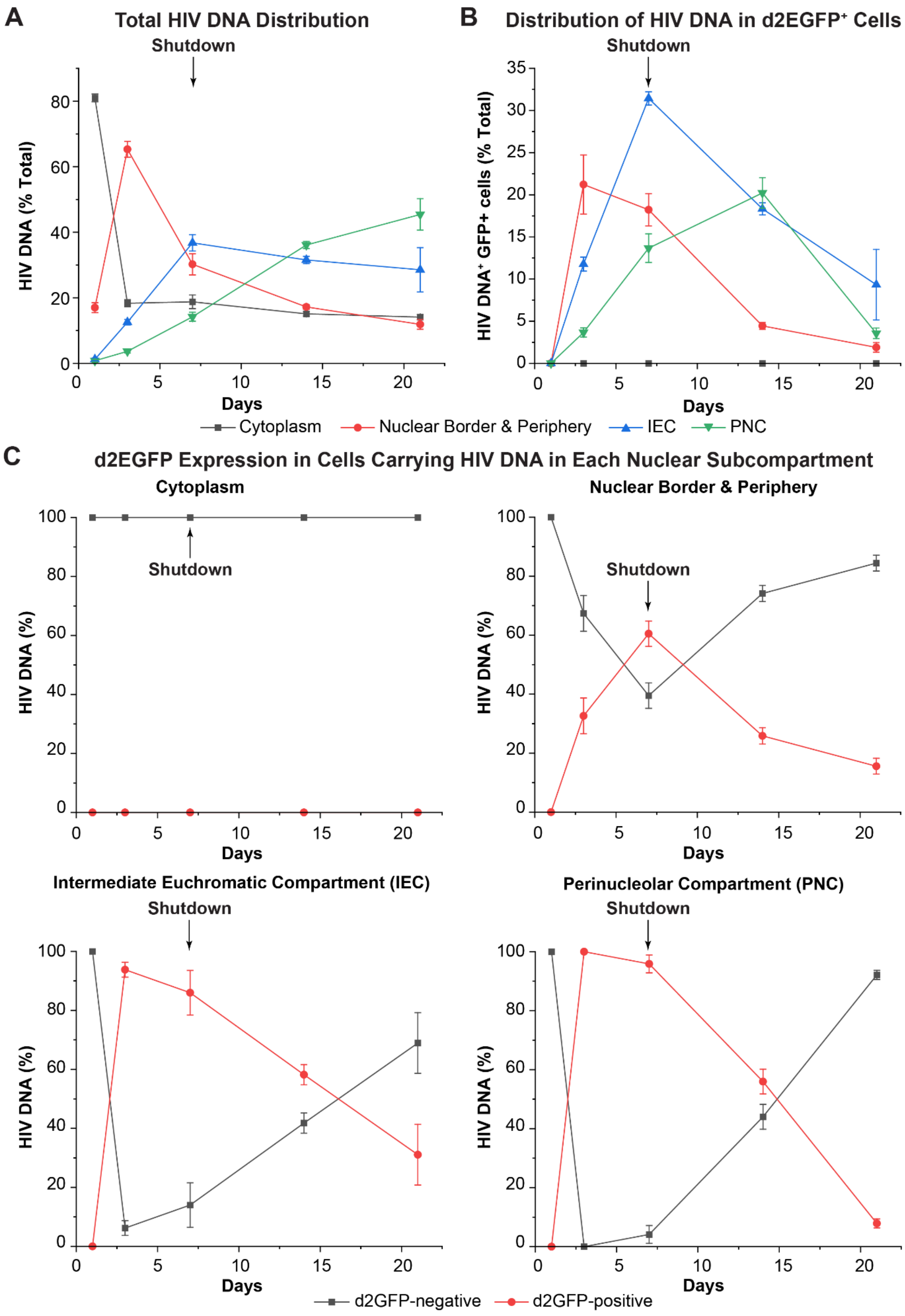
A
Total HIV DNA Distribution
Shutdown
HIV DNA (% Total)
Days
B
Distribution of HIV DNA in d2EGFP+ Cells
Shutdown
HIV DNA+ GFP+ cells (% Total)
Days
Cytoplasm
Nuclear Border & Periphery
IEC
PNC
C
d2EGFP Expression in Cells Carrying HIV DNA in Each Nuclear Subcompartment
Cytoplasm
Shutdown
HIV DNA (%)
Days
Nuclear Border & Periphery
Shutdown
HIV DNA (%)
Days
Intermediate Euchromatic Compartment (IEC)
Shutdown
HIV DNA (%)
Days
Perinucleolar Compartment (PNC)
Shutdown
HIV DNA (%)
Days
d2GFP-negative
d2GFP-positive

Fig. S8. Distribution of HIV DNA in cells expressing d2EGFP. (A) Subcellular distribution of HIV DNA in cells infected with the pHR'-d2EGFP reporter virus (% Total HIV DNA^{+} cells in each region per time point). Note that this data set includes HIV DNA found in the cytoplasm. (B) Subnuclear distribution HIV DNA found in GFP^{+} cells from the same experiment (% Total HIV DNA^{+} in GFP+ cells per time point). (C) Fraction of d2EGFP-expressing cells carrying HIV DNA in each sub-nuclear compartment. Nearly 100% of cells carrying HIV DNA in the IEC and PNC at Days 4 and 7 also express d2EGFP, indicating that these cells had transcriptionally active integrated proviruses. As cells become quiescent, the fraction of d2EGFP+ cells declined, consistent with the flow cytometry data shown in **Fig. S5B**.

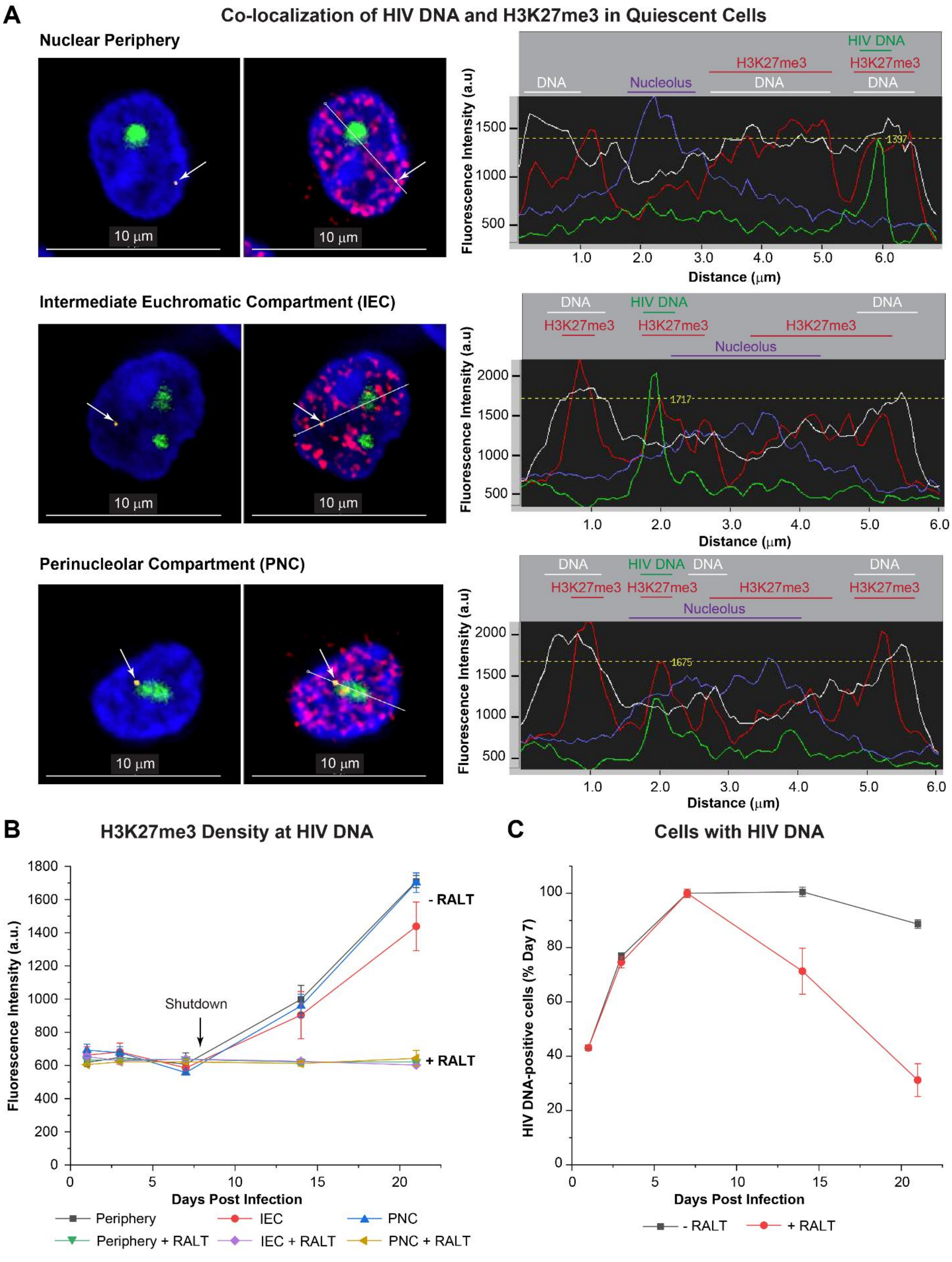

A
Co-localization of HIV DNA and H3K27me3 in Quiescent Cells
Nuclear Periphery
10 μm
HIV DNA
H3K27me3
DNA
Nucleolus
Fluorescence Intensity (a.u)
Distance (μm)
Intermediate Euchromatic Compartment (IEC)
Perinucleolar Compartment (PNC)
B
H3K27me3 Density at HIV DNA
Fluorescence Intensity (a.u.)
Shutdown
- RALT
+ RALT
Days Post Infection
Periphery
IEC
PNC
Periphery + RALT
IEC + RALT
PNC + RALT
C
Cells with HIV DNA
HIV DNA-positive cells (% Day 7)
Days Post Infection
- RALT
+ RALT

Fig. S9. Accumulation of H3K27me3 associated with HIV DNA during the transition of T-cells into quiescence. (A) Cells were infected with the pHR'-CD8a-d2EGFP reporter, and representative images of HIV DNA (stained using Alexa Fluor 555-labeled secondary antibody to dCas9, shown in yellow). were found in either the nuclear periphery (Day 3), the IEC (Day 7, or the PNC in quiescent cells (Day 21). The white line in the micrograph corresponds to the cross-section scanned on the right. Note the colocalization of HIV DNA with the H3K27me3 which was stained using Alexa Fluor 647-labeled antibody. The dotted lines on the scans show the maximum fluorescent intensity for H3K27me3 that colocalized with the HIV DNA. (B) The accumulation of H3K27me3 colocalizing with HIV DNA as the cells become quiescent was measured by line scans for 3 sets of 100 individual DNA puncta. Note that the extent of H3K27me3 accumulation in association with HIV DNA is equivalent in each of the subnuclear compartments. The accumulation of H3K27me3 during the shutdown was blocked by the addition of 10 μM RALT, consistent with the HIV DNA persisting in a non-integrated form. In this experiment, were evaluated in a mixed population (without the CD8a selection on Day 7) since purifying the RALT-treated samples which do not express CD8a-GFP was impossible. (C) Decline in non-integrated DNA as cells enter quiescence. The fraction of nuclei carrying HIV DNA puncta was measured in 3 sets of 100 individual DNA puncta. The data was normalized to the peak of infected cells at Day 7. Note the HIV DNA persists in the untreated cells, but 69% of the unintegrated HIV DNA puncta were lost from the cell populations as the cells became quiescent.

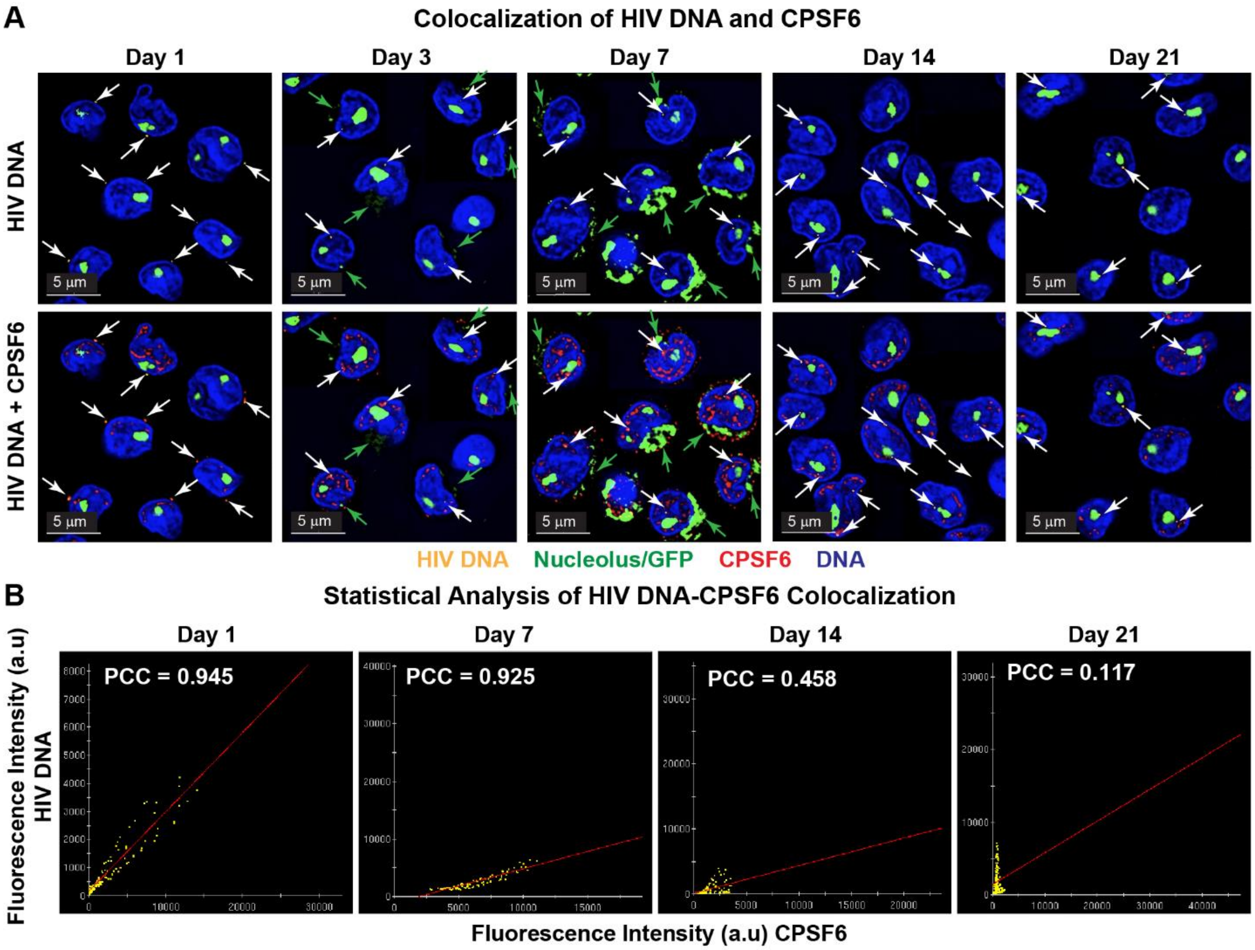
A
Colocalization of HIV DNA and CPSF6
Day 1
Day 3
Day 7
Day 14
Day 21
HIV DNA
HIV DNA + CPSF6
5 µm
HIV DNA
Nucleolus/GFP
CPSF6
DNA
B
Statistical Analysis of HIV DNA-CPSF6 Colocalization
Day 1
Day 7
Day 14
Day 21
PCC = 0.945
PCC = 0.925
PCC = 0.458
PCC = 0.117
Fluorescence Intensity (a.u) HIV DNA
Fluorescence Intensity (a.u) CPSF6

Fig. S10. Colocalization of HIV DNA and CPSF6 in primary T cells as they progress from acute infection to latency. (A) Representative micrographs of cells from the same experiment, as shown in **Fig. 3.** Cells were infected with the pHR'-CD8a-GFP virus. Yellow: CasFISH detection of HIV DNA stained using Alexa Fluor 555-labeled secondary antibody to dCas9. Red: immunofluorescence staining of CPSF6 using Alexa Fluor 647-labeled antibody. Green: Nucleoli stained with Fluorescein-labeled 45S pre-rRNA intron-specific Stellaris probes. Blue: DNA stained using DAPI. Scale bars represent a length of 5 μm, and the images were taken at 100X. White arrows indicate the location of the HIV DNA. Green arrows indicate GFP produced due to HIV transcription in the cells at Days 3 and 7. (B) Statistical analysis of the HIV DNA-CPSF6 colocalization. The values shown on the graphs represent the Pearson's correlation coefficient (PCC)/ r, which reflects the degree of colocalization between HIV DNA and CPSF6.

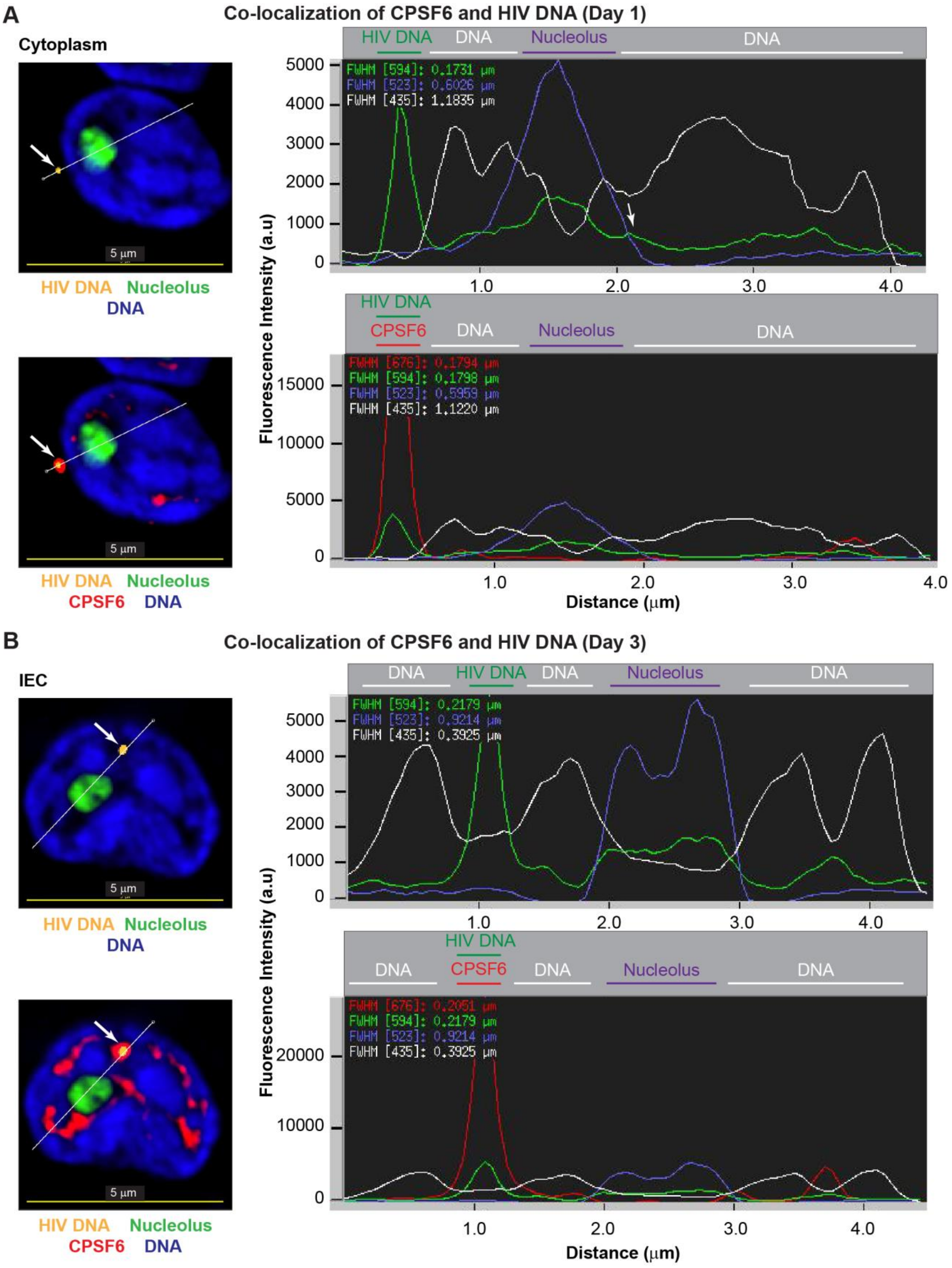
A
Co-localization of CPSF6 and HIV DNA (Day 1)
Cytoplasm
5 µm
HIV DNA Nucleolus
DNA
HIV DNA DNA Nucleolus DNA
FWHM [594]: 0.1731 µm
FWHM [523]: 0.6026 µm
FWHM [435]: 1.1835 µm
HIV DNA Nucleolus
CPSF6 DNA
HIV DNA
CPSF6 DNA Nucleolus DNA
FWHM [676]: 0.1794 µm
FWHM [594]: 0.1798 µm
FWHM [523]: 0.5959 µm
FWHM [435]: 1.1220 µm
Fluorescence Intensity (a.u)
Distance (µm)
B
Co-localization of CPSF6 and HIV DNA (Day 3)
IEC
DNA HIV DNA DNA Nucleolus DNA
FWHM [594]: 0.2179 µm
FWHM [523]: 0.9214 µm
FWHM [435]: 0.3925 µm
HIV DNA
DNA CPSF6 DNA Nucleolus DNA
FWHM [676]: 0.2051 µm
FWHM [594]: 0.2179 µm
FWHM [523]: 0.9214 µm
FWHM [435]: 0.3925 µm
Fluorescence Intensity (a.u)
Distance (µm)

Fig. S11. Line scanning analysis of the colocalization of HIV DNA and CPSF6. (A) Representative micrographs and line scanning analysis were used to measure proviral association with Alexa Fluor 647-secondary antibody labeled CPSF6 at Day 1 post-infection. HIV DNA detection using Alexa Fluor 555-secondary antibody-labeled dCas9 (top and bottom panels; yellow) and colocalization with CPSF6 (bottom panels; red). Nucleolar staining with Fluorescein-labeled 45S pre-rRNA intron-specific FISH probes is shown in green. The white arrows indicate the location of the HIV DNA. The white line in the micrograph corresponds to the cross-section scanned on the right. (B) Representative micrographs and line scanning analysis were used to measure proviral association with CPSF6 at Day 3 post-infection. Cells were stained as described in **Fig. S4**.

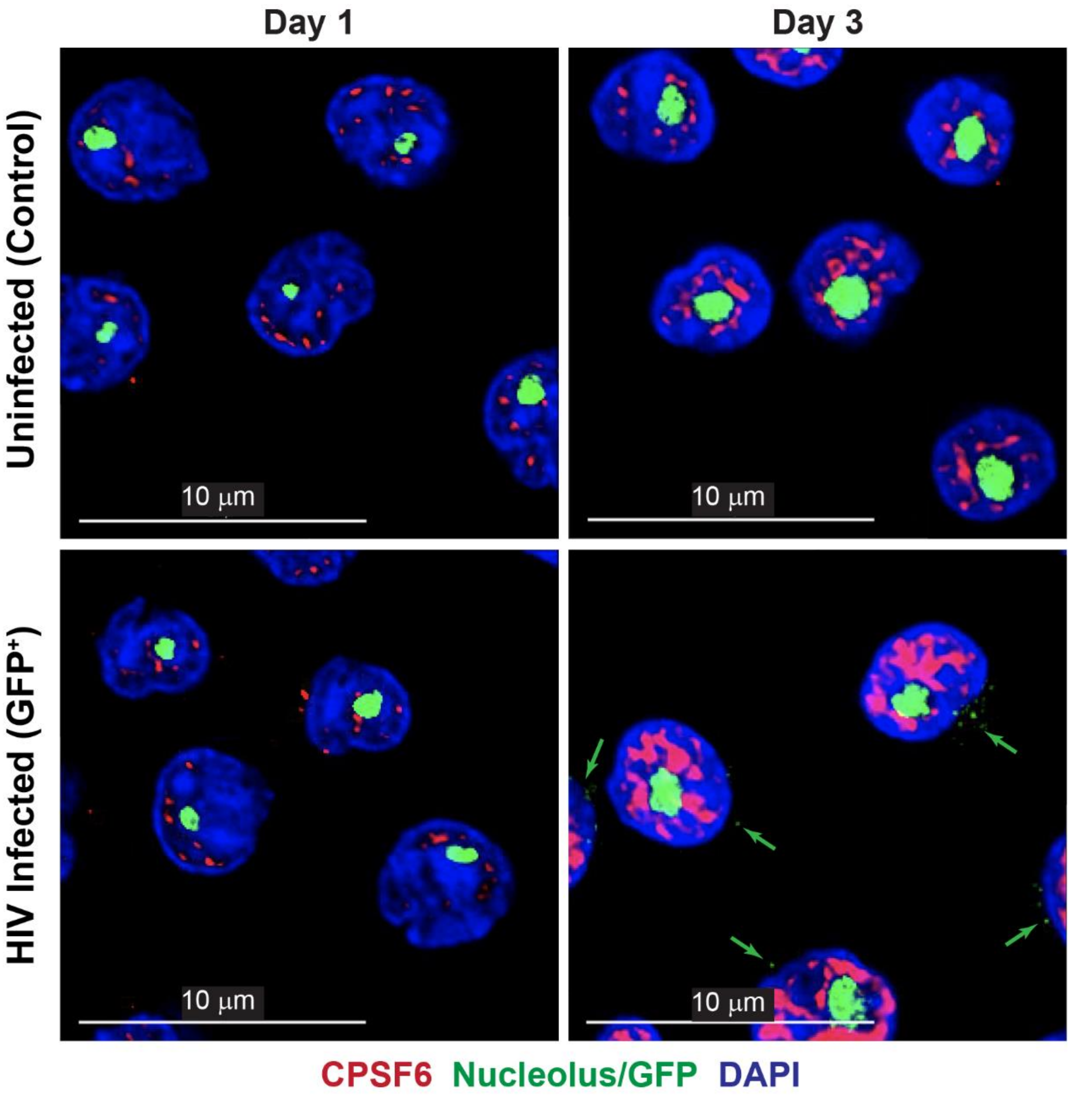

Day 1
Day 3
Uninfected (Control)
HIV Infected (GFP+)
10 µm
10 µm
10 µm
10 µm
CPSF6 Nucleolus/GFP DAPI

Fig. S12. Upregulation of CPSF6 in HIV-infected cells. (A) Representative uninfected control cells on Day 1 (Top left) and Day 3 (Top right) and HIV-infected cells on Day 1 (Bottom left) and Day 3 (Bottom right). Red: Alexa Fluor 647-labeled secondary antibody to CPSF6. Green: Nucleoli stained with Fluorescein-labeled 45S pre-rRNA intron-specific Stellaris probes. Green arrows low-level GFP expression in the cytoplasm of the cells at Day 3, demonstrating that these cells carry integrated and transcribed proviruses. Blue: DAPI (DNA).

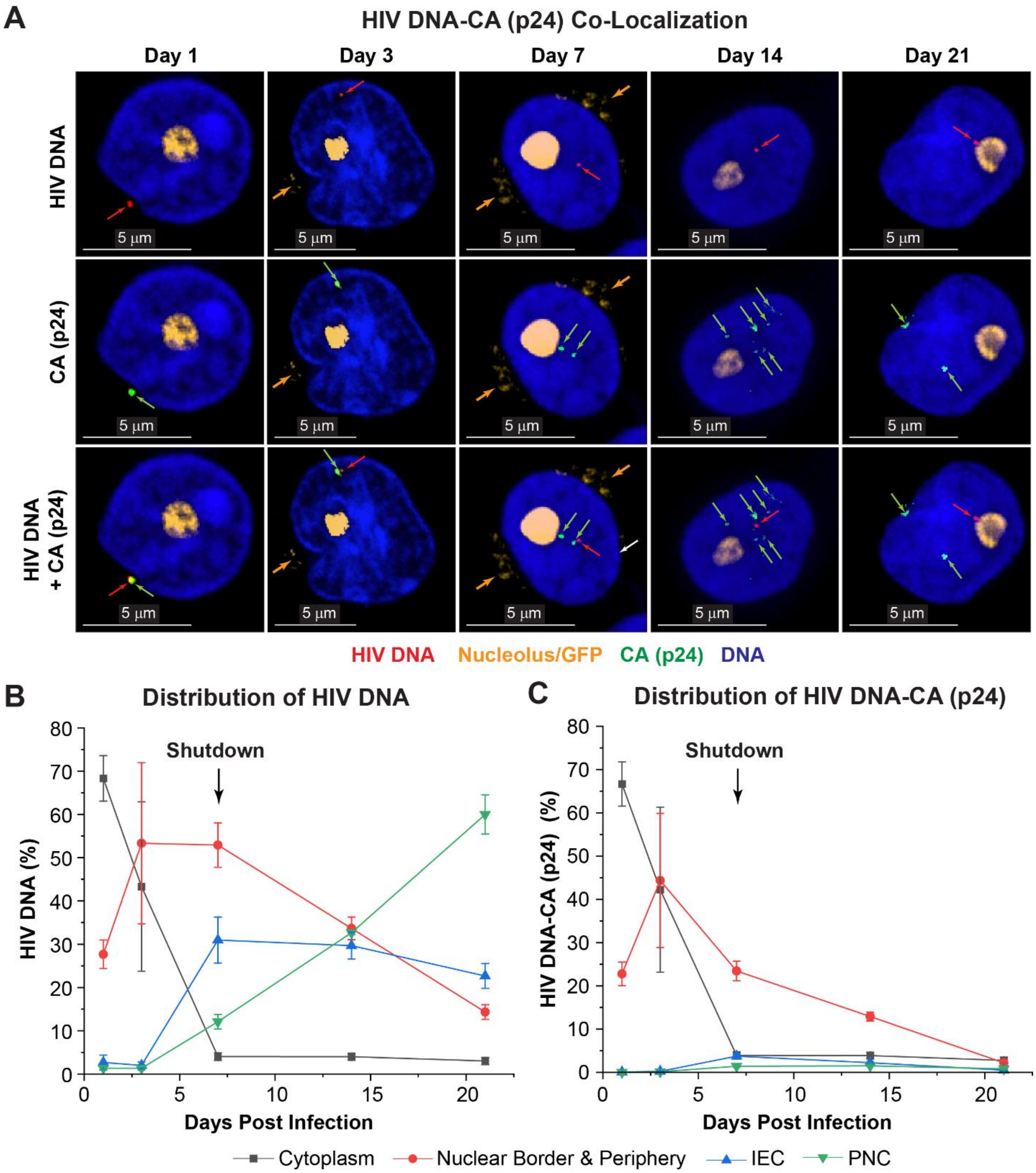
A
HIV DNA-CA (p24) Co-Localization
Day 1
Day 3
Day 7
Day 14
Day 21
HIV DNA
CA (p24)
HIV DNA + CA (p24)
5 µm
HIV DNA
Nucleolus/GFP
CA (p24)
DNA
B
Distribution of HIV DNA
Shutdown
HIV DNA (%)
Days Post Infection
C
Distribution of HIV DNA-CA (p24)
Shutdown
HIV DNA-CA (p24) (%)
Days Post Infection
Cytoplasm
Nuclear Border & Periphery
IEC
PNC

Fig. S13. Colocalization of HIV DNA and CA. (A) HIV DNA CasFISH combined with immunofluorescence staining of HIV CA (p24). Cells were infected with the pHR'-d2EGFP reporter virus. Staining was the same as in Fig. 3, but interchanged color labeling was introduced to improve visualization of the colocalized HIV DNA (red) and CA (p24) (green). Hence, nucleolar staining with Fluorescein-labeled 45S pre-rRNA intron-specific Stellaris probes is shown in orange, and the thick orange arrows indicate GFP produced from active HIV expression. Red arrows indicate the location of the HIV DNA. Light green arrows indicate HIV CA (p24). Images were taken at 100X. Scale: 5 μm. (B) Distribution of HIV DNA in the cytoplasm and indicated subnuclear compartments. (C) Distribution of the fraction of HIV DNA that colocalizes with CA in the cytoplasm and indicated subnuclear compartments. Error bars denote standard deviation.

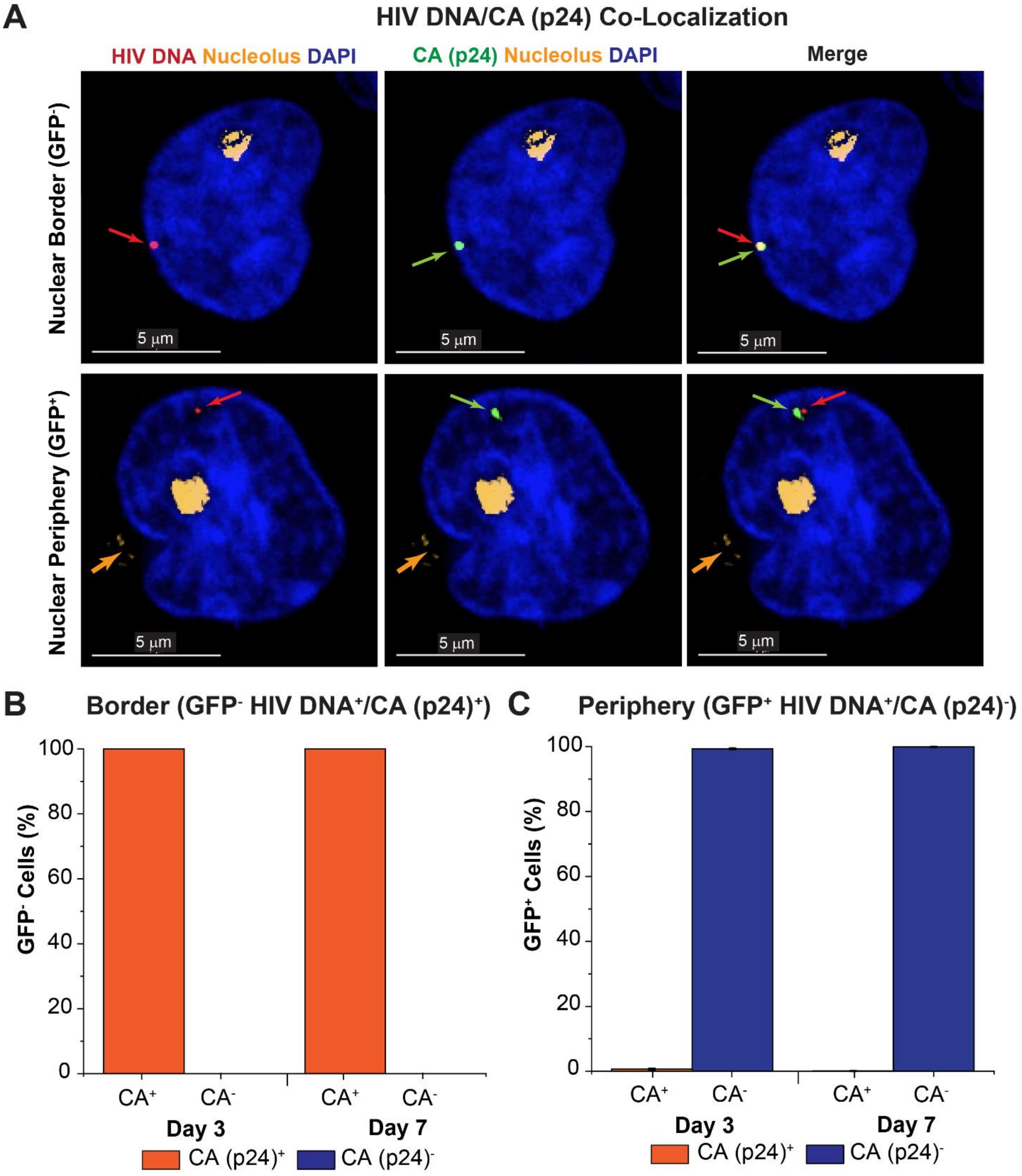

A
HIV DNA/CA (p24) Co-Localization
HIV DNA Nucleolus DAPI
CA (p24) Nucleolus DAPI
Merge
Nuclear Border (GFP-)
Nuclear Periphery (GFP+)
5 µm
B
Border (GFP- HIV DNA+/CA (p24)+)
GFP- Cells (%)
100
80
60
40
20
0
CA+
CA-
Day 3
Day 7
CA (p24)+
CA (p24)-
C
Periphery (GFP+ HIV DNA+/CA (p24)-)
GFP+ Cells (%)

Fig. S14. HIV DNA that colocalizes with CA is found primarily at the nuclear border in GFP- cells. (A) HIV DNA CasFISH combined with immunofluorescence staining of HIV CA (p24). Cells were infected with the pHR'-d2EGFP reporter virus. Staining was the same as in Fig. 3 and Fig S12, but interchanged color labeling was introduced to improve visualization of the colocalized HIV DNA (red) and CA (p24) (green). Hence the Red arrows indicate the location of the HIV DNA, Light green arrows indicate CA (p24), thick orange arrows indicate GFP produced because of active HIV expression and the nuclei stained with Fluorescein-labeled 45S pre-rRNA intron-specific Stellaris probes are shown in orange. Images were taken at 100X. Scale: 5 µm. (B) The fraction of d2EGFP-negative cells carrying HIV CA (p24) that colocalized with HIV DNA in the nuclear border region. Out of 200 cells with HIV DNA in the border region at Day 3 or Day 7, none of them expressed d2EGFP. (C) The fraction of d2EGFP-positive cells with HIV DNA in the nuclear periphery that colocalized with CA (p24). Greater than 99% of cells expressing d2EGFP had HIV DNA that was no longer colocalized with CA (p24). Data is shown for Days 3 and 7. Error bars denote standard deviation.

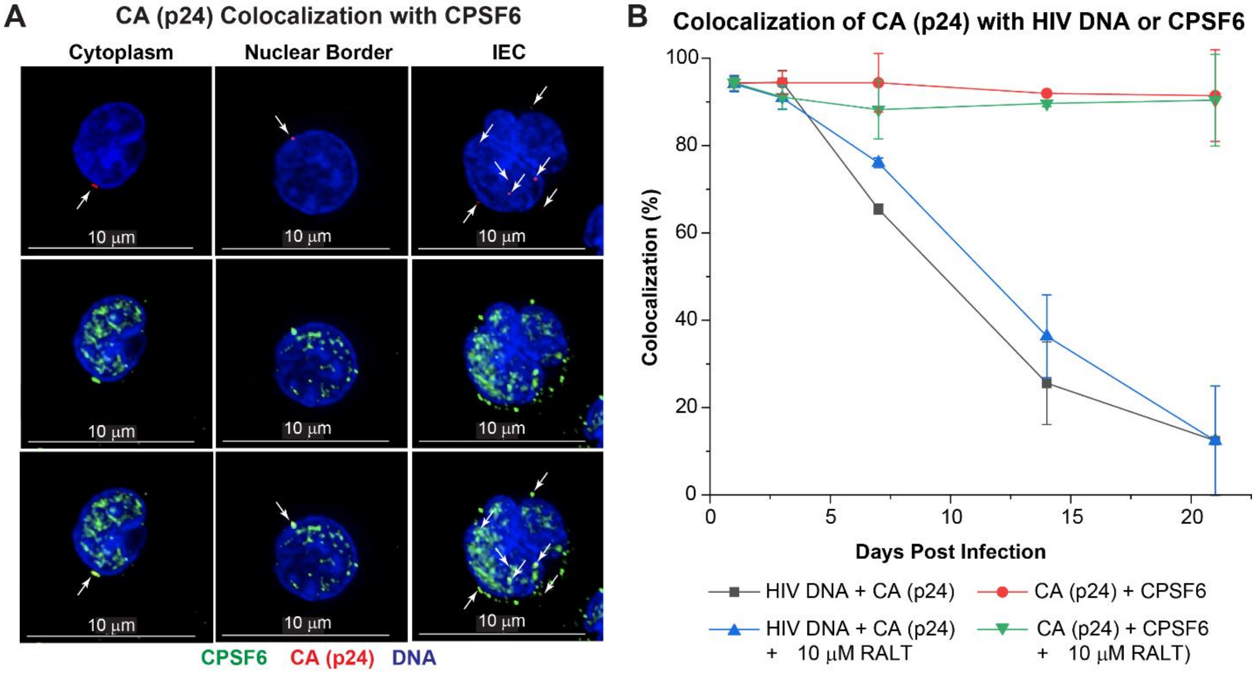

A
CA (p24) Colocalization with CPSF6
Cytoplasm
Nuclear Border
IEC
10 μm
CPSF6
CA (p24)
DNA
B
Colocalization of CA (p24) with HIV DNA or CPSF6
Colocalization (%)
Days Post Infection
0
20
40
60
80
100
0
5
10
15
20
HIV DNA + CA (p24)
CA (p24) + CPSF6
HIV DNA + CA (p24) + 10 μM RALT
CA (p24) + CPSF6 + 10 μM RALT)

Fig. S15. Subcellular distribution and colocalization of CA and CPSF6 in acutely infected primary Th17 cells. (A) Representative images showing the association of Alexa Fluor 647-secondary antibody labeled CA with Alexa Fluor 488-secondary antibody labeled CPSF6 in the cytoplasm (from Day 1), at the nuclear border (Day 1), and dispersed within the nucleus and cytoplasm (Day 3). Cells were infected with the pHR'-CD8a-GFP virus. The images were captured at 60X, and the scale bars represent a length of 10 μm. (B) The degree of colocalization between HIV DNA and CA (p24) (% Total HIV DNA) or between CA (p24) and CPSF6 (% Total CA (p24)) in pHR'-CD8a-GFP infected cells over the course of the QUECEL procedure. The experiment was performed in the presence or absence of 10 μM RALT. CA (p24) remained highly associated with CPSF6 at each time point and in the presence or absence of RALT. By contrast, CPSF6 does not remain associated with HIV DNA and is virtually absent from the HIV DNA in the quiescent cells. Error bars denote standard deviation.

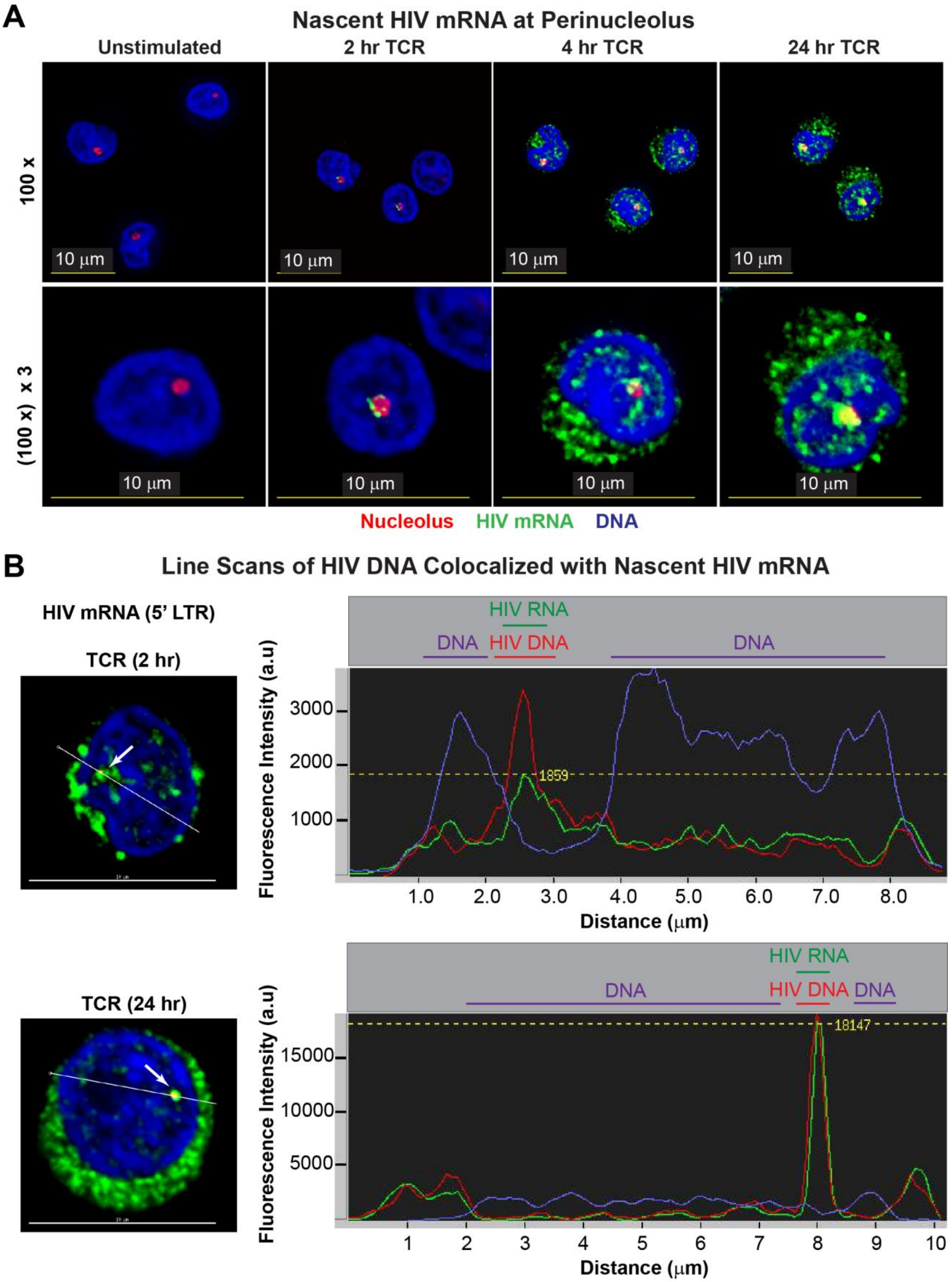
A
Nascent HIV mRNA at Perinucleolus
Unstimulated
2 hr TCR
4 hr TCR
24 hr TCR
100 x
(100 x) x 3
10 μm
Nucleolus HIV mRNA DNA
B
Line Scans of HIV DNA Colocalized with Nascent HIV mRNA
HIV mRNA (5' LTR)
TCR (2 hr)
HIV RNA
DNA
HIV DNA
DNA
1859
Fluorescence Intensity (a.u)
Distance (μm)
TCR (24 hr)
HIV RNA
DNA
HIV DNA
DNA
18147
Fluorescence Intensity (a.u)
Distance (μm)

Fig. S16. The PNC supports early proviral transcription. (A) Detection of nascent HIV mRNA in the PNC. Green: TAMRA-labeled HIV LTR-specific Stellaris RNA FISH probes. Red: Nucleoli stained with Quasar 670-labeled 45S pre-rRNA intron-specific Stellaris RNA FISH probes. Blue: DNA stained using DAPI. All scale bars represent a length of 5 µm. Images were taken at 100X and enlarged 3-fold. (B) Line scans showing colocalization of HIV RNA with HIV DNA following T-cell receptor stimulation for 2 hours or 24 hours. The white line in the micrograph corresponds to the cross-section scanned on the right. The white arrows indicate the location of the HIV DNA. The dotted lines on the scans show the maximum fluorescent intensity for HIV RNA that colocalized with the HIV DNA.

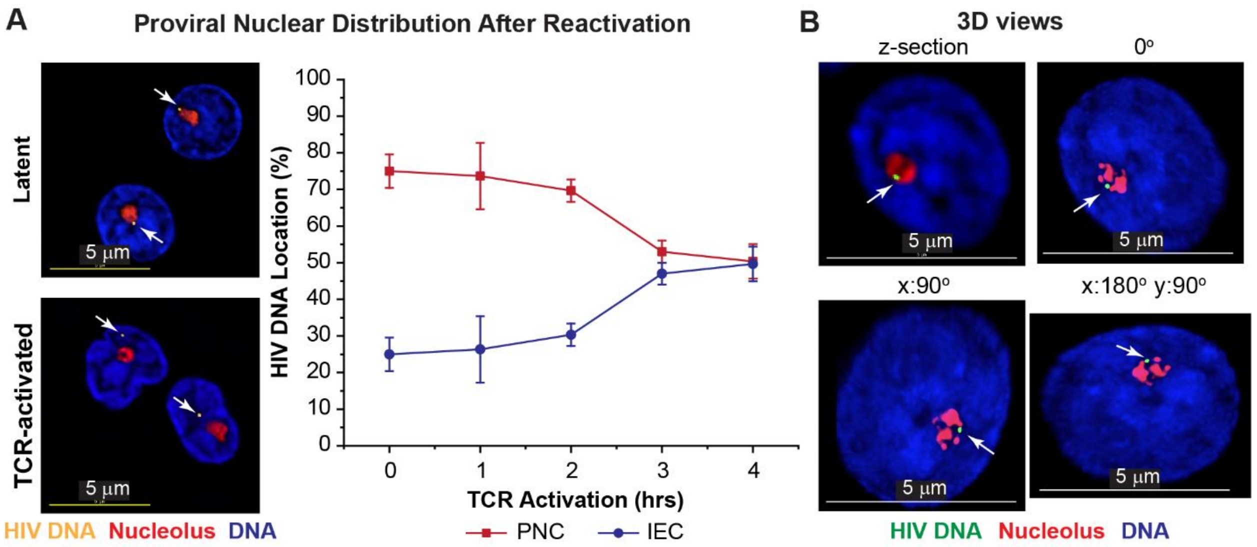

A
Proviral Nuclear Distribution After Reactivation
Latent
TCR-activated
5 μm
5 μm
HIV DNA Nucleolus DNA
HIV DNA Location (%)
TCR Activation (hrs)
PNC
IEC
B
3D views
z-section
0°
x:90°
x:180° y:90°
5 μm
HIV DNA Nucleolus DNA

Fig. S17. HIV Proviral DNA distributions after reactivation of quiescent T-cells. (A) HIV DNA detected in the PNC in unstimulated latently infected T cells (Top) and in the IEC after TCR activation for 24 hours (Bottom). The white arrows indicate the location of the HIV DNA detected by using Alexa Fluor 555-secondary antibody-labeled dCas9. Images were taken at 100X then enlarged 2-fold. Scale bars represent a length of 5 μm. The graph at right shows the shift in proviral localization following TCR activation. Three independent sets of 100 randomly selected cells were counted. Error bars denote standard deviation. (B) Rotated 2D views captured from 3D image reconstruction analysis showing localization of a provirus (white arrow; green staining) within 0.1 μm of the nucleolus in the PNC. The 45S pre-rRNA intron-specific FISH staining of the nucleolus is shown in red. The image shown was taken at 100X then enlarged 3-fold. Scale bars represent a length of 5 μm.

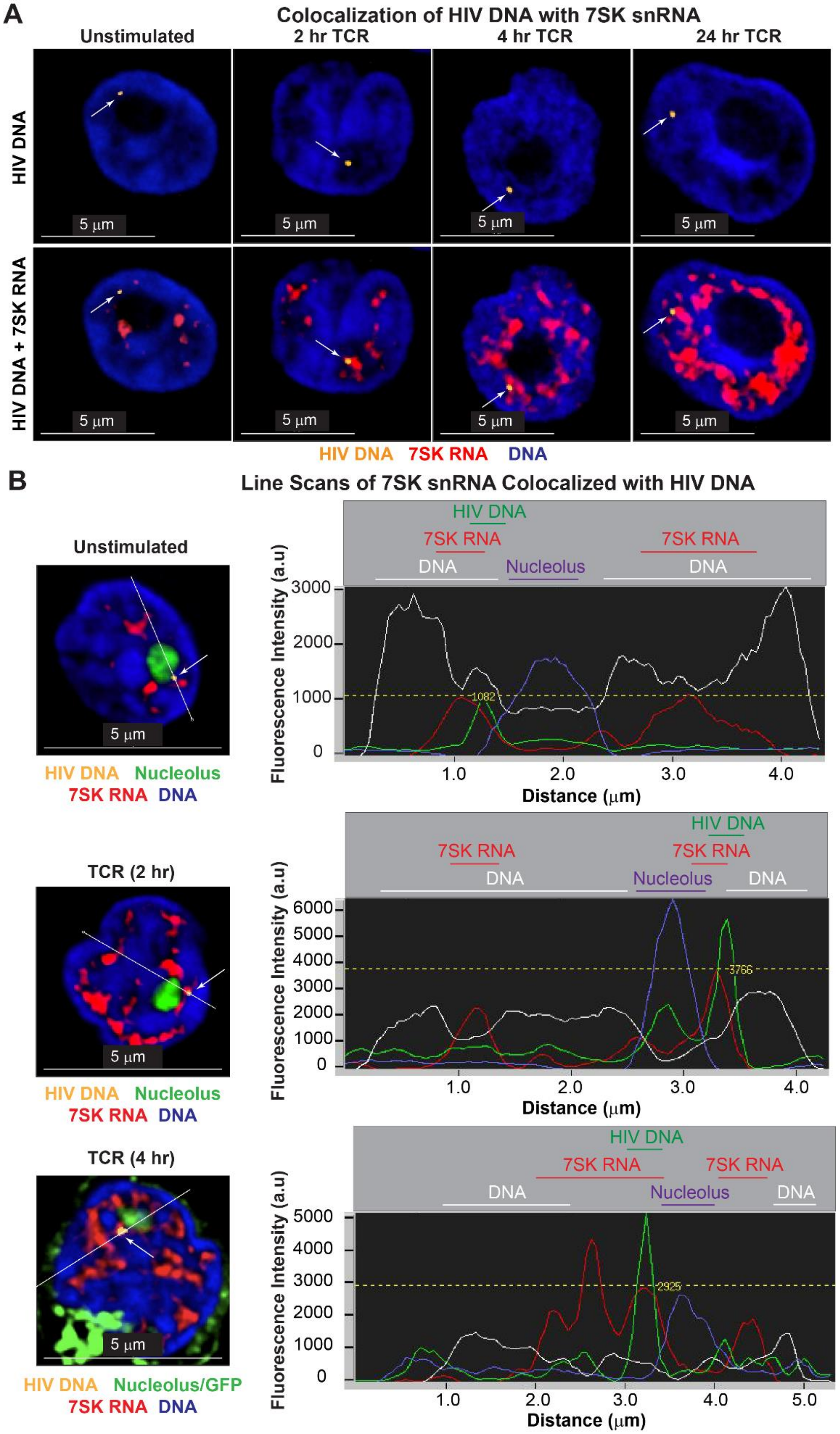

A
Colocalization of HIV DNA with 7SK snRNA
Unstimulated
2 hr TCR
4 hr TCR
24 hr TCR
HIV DNA
HIV DNA + 7SK RNA
5 μm
HIV DNA 7SK RNA DNA
B
Line Scans of 7SK snRNA Colocalized with HIV DNA
Unstimulated
HIV DNA Nucleolus
7SK RNA DNA
TCR (2 hr)
HIV DNA Nucleolus
7SK RNA DNA
TCR (4 hr)
HIV DNA Nucleolus/GFP
7SK RNA DNA
HIV DNA
7SK RNA
DNA
Nucleolus
Fluorescence Intensity (a.u)
Distance (μm)
1082
3766
2925

Fig. S18. Synthesis of 7SK snRNA and HIV mRNA during the reactivation of latent proviruses by TCR stimulation of quiescent T cells. (A) Time course showing the colocalization of 7SK snRNA with HIV proviruses. Yellow: HIV proviral DNA (Alexa Fluor 555). Red: Quasar 670-labeled Stellaris RNA FISH probes to 7SK snRNA. Blue: DNA stained using DAPI. White arrows indicate the location of the HIV DNA detected by using Alexa Fluor 555-secondary antibody-labeled dCas9. (B) Line scans showing colocalization of 7SK snRNA with HIV DNA in unstimulated cells and following T-cell receptor stimulation for 2 hours or 4 hours. The white line in the micrograph corresponds to the cross-section scanned on the right. The white arrows indicate the location of the HIV DNA. The dotted lines on the scans show the maximum fluorescent intensity for 7SK snRNA that colocalized with the HIV DNA.

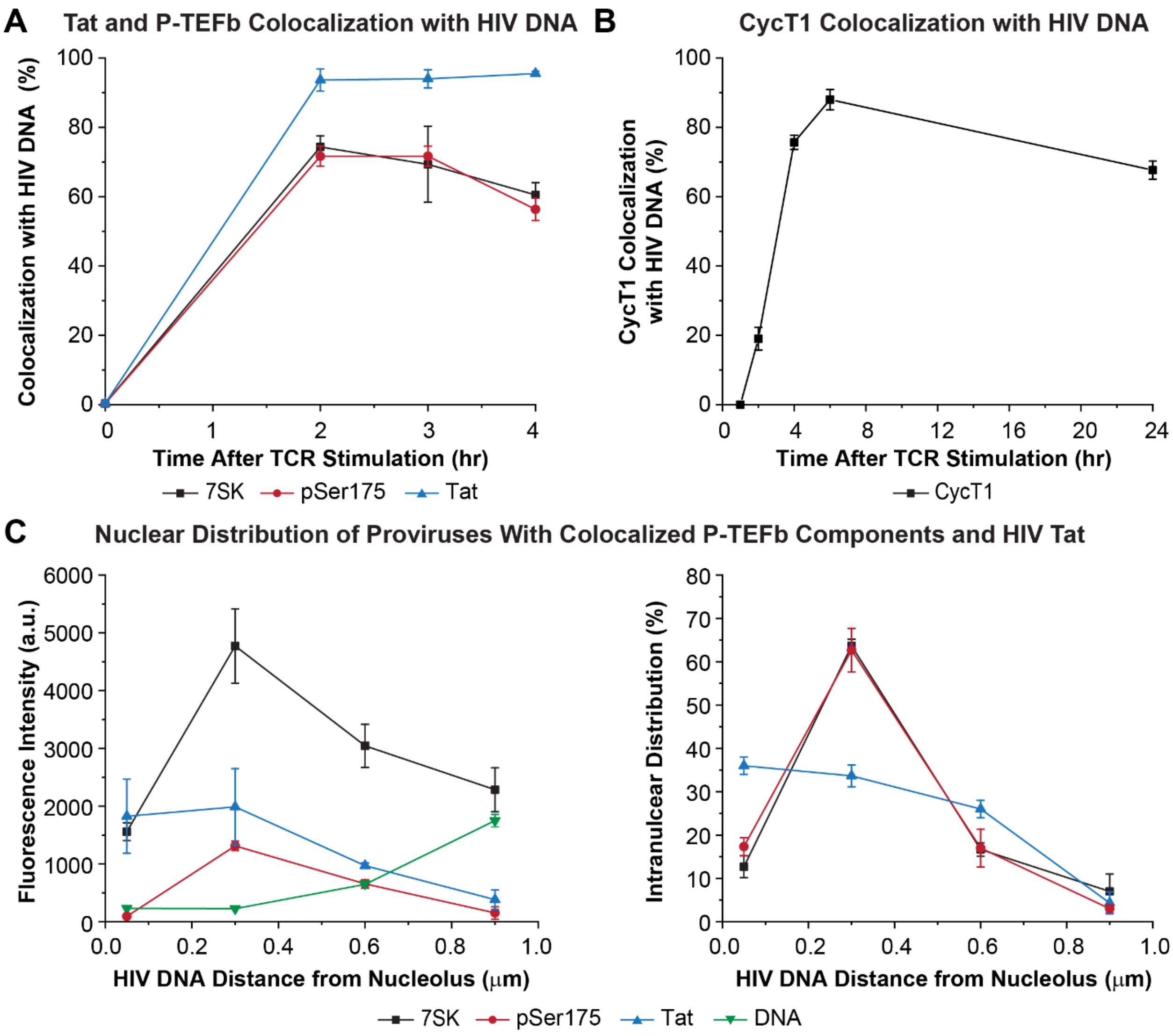
A
Tat and P-TEFb Colocalization with HIV DNA
Colocalization with HIV DNA (%)
Time After TCR Stimulation (hr)
7SK
pSer175
Tat
B
CycT1 Colocalization with HIV DNA
CycT1 Colocalization with HIV DNA (%)
Time After TCR Stimulation (hr)
CycT1
C
Nuclear Distribution of Proviruses With Colocalized P-TEFb Components and HIV Tat
Fluorescence Intensity (a.u.)
HIV DNA Distance from Nucleolus (μm)
Intranulcear Distribution (%)
HIV DNA Distance from Nucleolus (μm)
7SK
pSer175
Tat
DNA

Fig. S19. Recruitment of the P-TEFb machinery to HIV DNA following reactivation of quiescent cells by T-cell receptor stimulation. (A) Time course showing accumulation of colocalized Tat and P-TEFb subunits with HIV DNA following TCR activation of latently infected cells. The fraction of HIV DNA colocalizing with 7SK RNA (black symbols), the pSer175 modified CDK9 subunit of P-TEFb (red symbols) and HIV Tat (blue symbols) between 1 and 4 hours after TCR stimulation of quiescent cells are shown. (B) Time course showing accumulation of colocalized CycT1 and HIV DNA. (C) Preferential assembly of HIV proviruses located in the PNC with P-TEFb. The distribution of HIV DNA between nuclear sub-compartments and their association with P-TEFb and Tat after 2 or 4 hours reactivation by TCR stimulation of quiescent T-cells. HIV DNA located between 0 – 0.09 μm (PNC), 0.1-0 – 0.49 μm (PNC), 0.5 - 0.79 μm (IEC) and 0.8 - 1.0 μm (nuclear periphery) from the nucleolus were binned. Error bars denote standard deviation. Most of the HIV DNA (75%) was found in the PNC (0 to 0.49 μm from the nucleolus) with only 17% found in the IEC (0.5 – 0.79 μm from the nucleolus). 100% of the proviruses in the PNC and IEC colocalized with 7SK RNA and P-TEFb (pSer175 CDK9). Tat accumulated disproportionately in the regions nearest the nucleolus. Left: Fluorescence intensity at the provirus. Right: Distribution of proviruses associated with individual P-TEFb components throughout the nuclear sub-compartments.

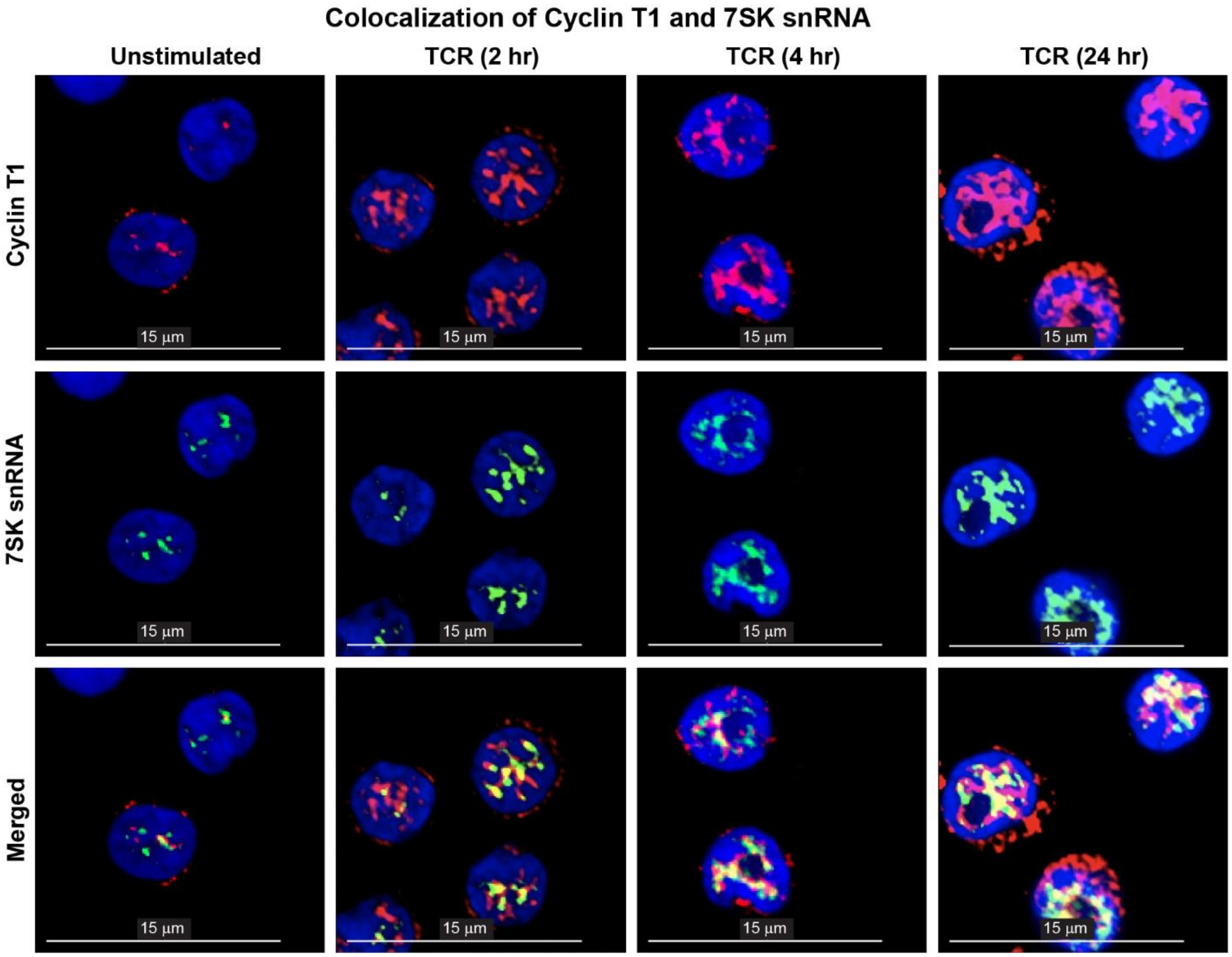
Colocalization of Cyclin T1 and 7SK snRNA
Unstimulated
TCR (2 hr)
TCR (4 hr)
TCR (24 hr)
Cyclin T1
7SK snRNA
Merged
15 µm

Fig. S20. Colocalization of Cyclin T1 and 7SK snRNA in quiescent and reactivated cells. From left to right: unstimulated cells, and cells at 2, 4 and 24 hours after TCR activation. Top row: Cyclin T1 (Red) detected with an Alexa Fluor 647-labeled secondary antibody to a Cyclin T1 primary antibody. Middle row: 7SK snRNA (Green) detected by hybridization to TAMRA-labeled Stellaris RNA FISH probes. Bottom row: Merged images. Blue: DNA stained using DAPI. Scale bars represent a length of 15 µm. Images were taken at 60X and enlarged 2-fold.

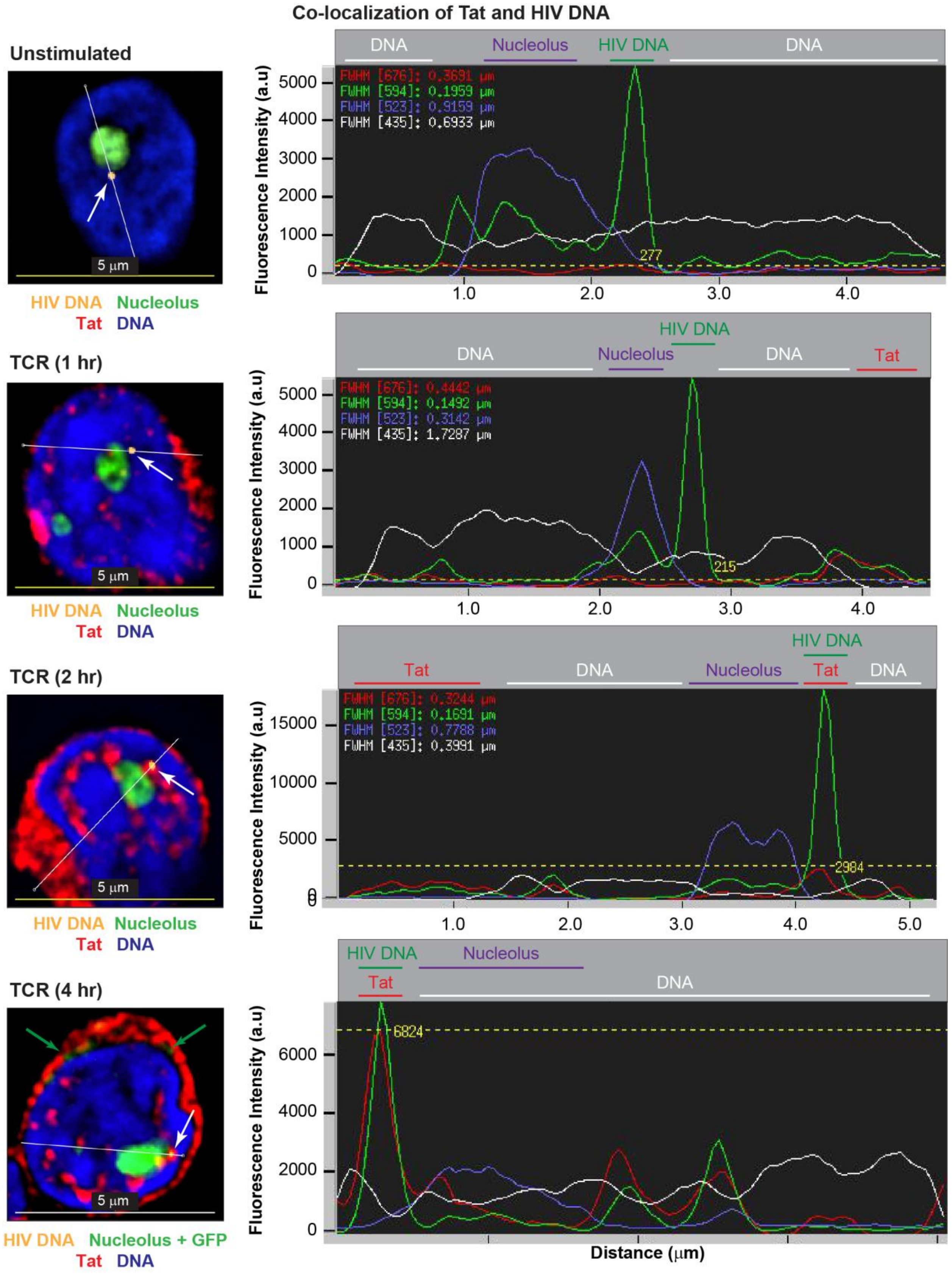

Co-localization of Tat and HIV DNA
Unstimulated
HIV DNA Nucleolus
Tat DNA
TCR (1 hr)
HIV DNA Nucleolus
Tat DNA
TCR (2 hr)
HIV DNA Nucleolus
Tat DNA
TCR (4 hr)
HIV DNA Nucleolus + GFP
Tat DNA
5 μm
DNA
Nucleolus
HIV DNA
Tat
FWHM [676]: 0.3691 μm
FWHM [594]: 0.1959 μm
FWHM [523]: 0.9159 μm
FWHM [435]: 0.6933 μm
FWHM [676]: 0.4442 μm
FWHM [594]: 0.1492 μm
FWHM [523]: 0.3142 μm
FWHM [435]: 1.7287 μm
FWHM [676]: 0.3244 μm
FWHM [594]: 0.1691 μm
FWHM [523]: 0.7788 μm
FWHM [435]: 0.3991 μm
277
215
2984
6824
Fluorescence Intensity (a.u)
Distance (μm)

Fig. S21. Colocalization of HIV DNA and Tat after proviral reactivation. Representative micrographs and line scanning analysis to measure proviral association with Tat in unstimulated cells and 1, 2, or 4 hours after TCR activation of the $CD4^+$ T cells. The white line in the micrograph corresponds to the cross-section scanned on the right. The white arrows indicate the location of the HIV DNA. The dotted lines on the scans show the maximum fluorescent intensity for Tat that colocalized with the HIV DNA. Note the production of GFP in the cytoplasm of the activated $CD4^+$ T cells at 4 hours (green arrows). Scale bars represent a length of 5 μm. Images were taken at 100X and enlarged 3-fold.

A Line Scans of Preferential Tat Recruitment in the Perinucleolus

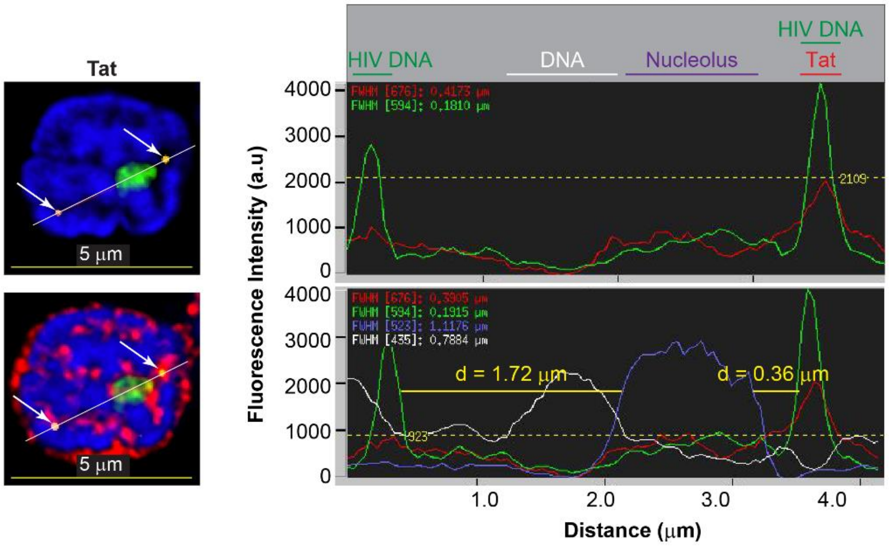

B Line Scans of Preferential Cyclin T1 Recruitment in the Perinucleolus

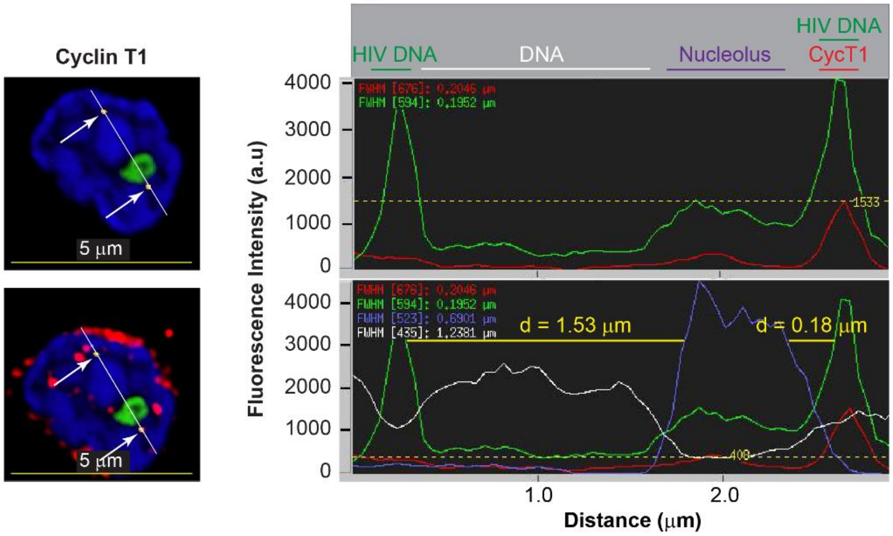

C Line Scans of Preferential 7SK snRNA Recruitment in the Perinucleolus

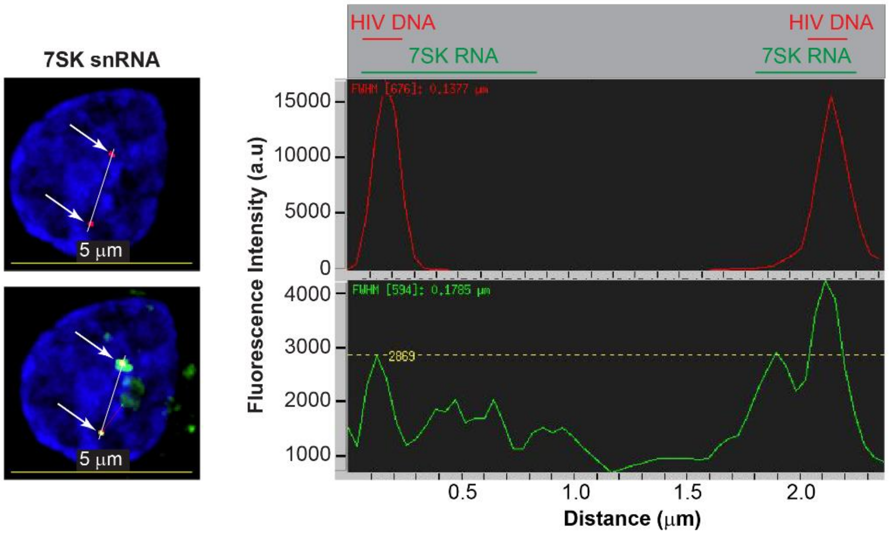

Fig. S22. Preferential reactivation of HIV proviruses in the PNC of cells carrying two HIV DNAs. For this analysis the 15% of latently infected cells that carried two HIV DNA puncta were analyzed. (A) Representative micrographs and line scanning analysis to measure HIV DNA association with Tat 4, hours after TCR activation of the $CD4^+$ T cells. The white line in the micrograph corresponds to the cross-section scanned on the right. The white arrows indicate the location of the HIV DNAs. The dotted lines on the scans show the maximum fluorescent intensity for Tat that colocalized with the HIV DNA in the PNC. The yellow bars show the distance of the HIV DNA from the nucleolar border. Note that the second HIV DNA locus found in the nuclear periphery was not associated with Tat. Scale bars represent a length of 5 μm. Images were taken at 100X and enlarged 3-fold. (B) Representative micrographs and line scanning analysis to measure proviral association with CycT1 4 hours after TCR activation of the $CD4^+$ T cells. The white line in the micrograph corresponds to the cross-section scanned on the right. The white arrows indicate the location of the HIV DNAs. The dotted line on the top scan showed the maximum fluorescent intensity for CycT1 that colocalized with the HIV DNA in the PNC. The dotted line on the bottom scan showed the maximum fluorescent intensity for CycT1 that colocalized with the HIV DNA in the IEC. (C) Representative micrographs and line scanning analysis to measure HIV DNA association with 7SK snRNA 4 hours after TCR activation of the $CD4^+$ T cells. The white line in the micrograph corresponds to the cross-section scanned on the right. Because the scan did not traverse the nucleolus there is no nucleolar signal shown. The white arrows indicate the location of the HIV DNAs. The dotted line on the bottom scan showed the maximum fluorescent intensity for 7SK snRNA that colocalized with the HIV DNA in the PNC. The HIV DNA in the PNC was 0.304 μm from the nucleolar border, while the second HIV DNA locus in the IEC was 0.921 μm from the nucleolar border.

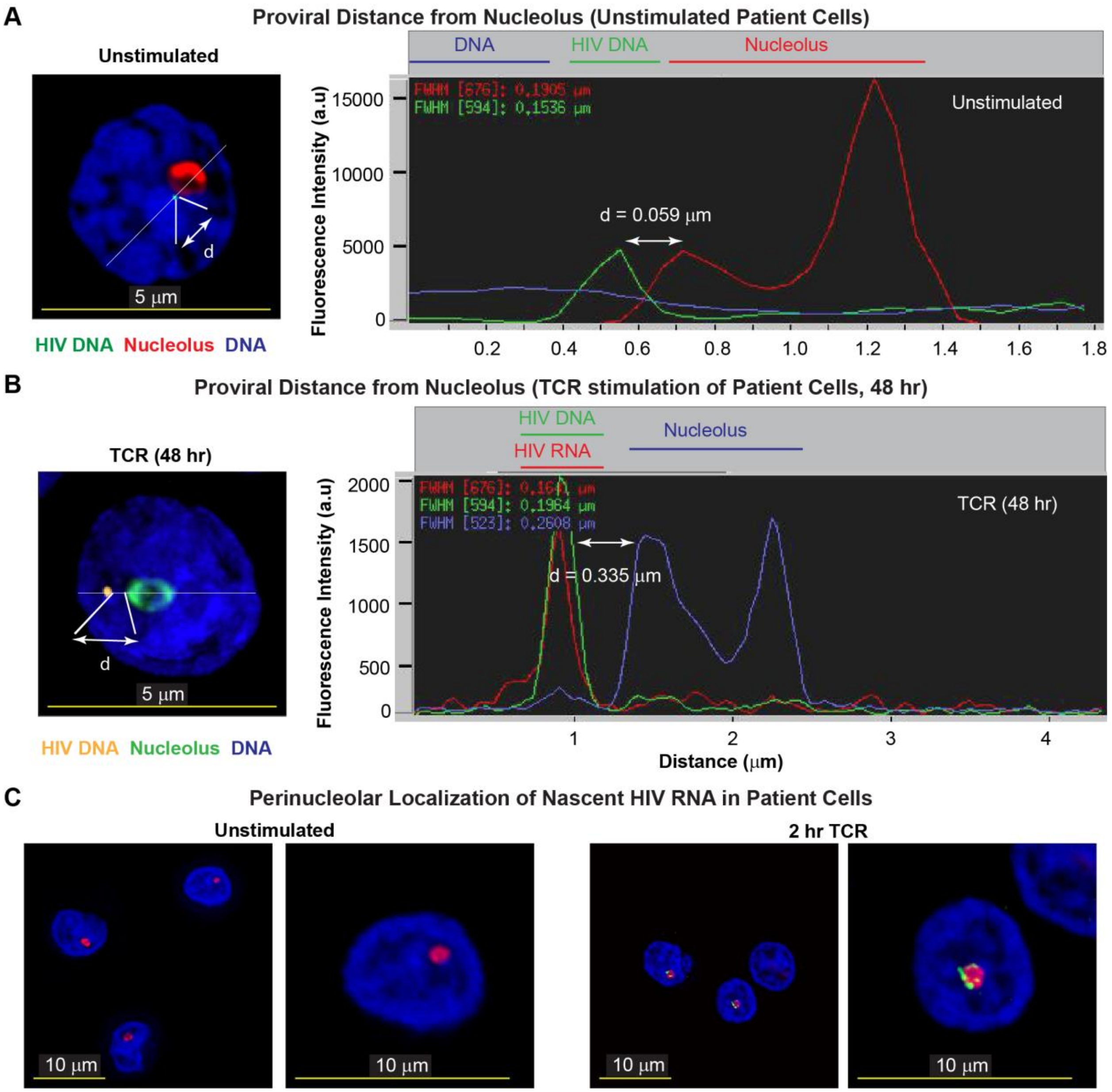

A
Proviral Distance from Nucleolus (Unstimulated Patient Cells)
Unstimulated
5 μm
HIV DNA Nucleolus DNA
DNA HIV DNA Nucleolus
FWHM [676]: 0.1905 μm
FWHM [594]: 0.1536 μm
Unstimulated
d = 0.059 μm
Fluorescence Intensity (a.u)
B
Proviral Distance from Nucleolus (TCR stimulation of Patient Cells, 48 hr)
TCR (48 hr)
5 μm
HIV DNA Nucleolus DNA
HIV DNA
HIV RNA
Nucleolus
FWHM [594]: 0.1964 μm
FWHM [523]: 0.2608 μm
TCR (48 hr)
d = 0.335 μm
Fluorescence Intensity (a.u)
Distance (μm)
C
Perinucleolar Localization of Nascent HIV RNA in Patient Cells
Unstimulated
2 hr TCR
10 μm

Fig. S23. Proviral relocalization after reactivation of latently infected CD4+ memory T-cells from ART-suppressed donors. (A) Representative micrograph and line scanning analysis to measure the distance between the provirus and nucleolus in unstimulated quiescent cells. Green: HIV proviral DNA (Alexa 488-labeled secondary antibody to dCas9). Red: Nucleolus stained with Quasar670-labeled 45S pre-rRNA intron-specific Stellaris probes. Blue: DNA stained using DAPI. (B) Representative micrograph and line scanning analysis to measure the distance between the provirus and nucleolus 48 hour after TCR activation. Note the relocation of the provirus away from the nucleolus. Yellow: HIV proviral DNA (Alexa 555-labeled secondary antibody to dCas9). Green: Nucleolus stained with Fluorescein-labeled 45S pre-rRNA intron-specific Stellaris probes. Blue: DNA stained using DAPI. Scale bars represent a length of 5 µm. Images were taken at 100X and enlarged 3-fold. (C) Perinucleolar localization of nascent HIV RNA transcripts. Green: TAMRA-labeled HIV LTR-specific Stellaris RNA FISH probes. Red: Nucleoli stained with Quasar 670-labeled 45S pre-rRNA intron-specific Stellaris RNA FISH probes. Blue: DNA stained using DAPI. All scale bars in represent a length of 10 µm. Images were taken at 100X and enlarged 3-fold in the right-hand panels.

Table S1. Antibodies

Antigen	Antibody Type	Catalogue Number	Supplier
Capsid (p24)	Mouse Monoclonal	ARP-6457<br>ARP-6521<br>ARP-6458	NIH HIV Reagent Program Division of AIDS Contributed by Michael Malim Ref (6, 7)
CPSF6	Rabbit polyclonal	PA5-103749	Thermo Fisher Scientific
CycT1	Rabbit polyclonal	PA5-78944	Thermo Fisher Scientific
CPSF6	Rabbit monoclonal	EPR12898	Abcam
Lamin B1	Rabbit polyclonal	ab16048	Abcam
Cas9	Mouse mAb	#48989	Cell Signaling
Nuclear pore complex proteins	Alexa Fluor® 594 antibody		BioLegend
PTBP1	Rabbit monoclonal	EPR9048(B)	Abcam
phospho-Ser175	Rabbit polyclonal	In house	Ref (8).
Tat	Mouse monoclonal NT3/2D1.1 monoclonal antibody	In house	Ref (9)

Table S2. CasFISH gRNA Target Sequences and Methods

Probe target	Probe sequences (5’ to 3’)
**HIV LTR DNA**	TAGCTTGTAGCACCATCCAA,<br>GATATCCACTGACCTTTGGA,<br>CAAGGATATCTTGTCTTCGT,<br>TAGCACCATCCAAAGGTCAG,<br>CACTGACTAAAAGGGTCTGA,<br>GACAAGATATCCTTGATCTG,

Corresponding guide RNA sequences (Alt-R® CRISPR-Cas9 crRNA) were prepared (Integrated DNA Technologies) to interact with purified dCas9 through a short tracrRNA sequence (Alt-R® CRISPR-Cas9 tracrRNA). To assemble the complex, each Alt-R® CRISPR-Cas9 crRNA was individually incubated with the Alt-R® CRISPR-Cas9 tracrRNA in a nuclease-free duplex buffer at 95 °C for 5 min. After cooling, the mixture was added to the dCas9 protein and incubated at room temperature for 10 min before maintaining it at 4 °C.

Table S3. Buffer Composition

Buffer Name	Purpose	Composition
**Buffer A**	dCas9/gRNA complex	20 mM Hepes (pH 7.5), 100 mM KCl, 5 mM $MgCl_2$, 10% BSA, 5% Glycerol, 0.1% Tween 20 and freshly added 1 mM DTT
**Buffer B**	dCas9 storage buffer	20 mM Tris-HCl (pH 7.4), 0.2 M NaCl, 1 mM EDTA, 1 mM TCEP and 20% glycerol
**Buffer C**	dCas9/gRNA complex	10% Donkey IgG or 10% Normal Donkey Serum in Buffer A
**Buffer D**	Wash buffers	1X Perm/Wash, 0.1% Tween-20 in 1X PBS, 1X PBS alone,
**Buffer E**	Permeabilization buffer	0.2% Triton X-100 in 1X PBS
**Buffer F**	Blocking buffers	10% Normal Donkey Serum in 1X Perm/Wash,
**Buffer G**	Hybridization buffer	10% dextran sulfate, 2X SSC, 10% deionized formamide
**Buffer H**	RNA FISH wash buffer A	5 mls of 20X SSC (Invitrogen), 5 mls of deionized formamide, 40 mls nuclease-free water. Final: 2X SSC, 10% deionized formamide
**Buffer I**	RNA FISH wash buffer B	5 mls of 20X SSC (Invitrogen), 40 mls nuclease-free water
**Buffer J**	1X Perm/Wash buffer	5 mls of 10X Perm/Wash in 45 mls of nuclease-free water

RNA Cas-FISH: Cells on poly-L-lysine coated coverslips were fixed using 4% formaldehyde for 10 min followed by a second fixation-permeabilization step using a 1:1 ratio of pre-chilled (-20 °C) Methanol : Acetic acid mixture for 20 min. After washing with 0.1% Tween-20 (Fisher Scientific) in 1X PBS, the cells were further permeabilized using 0.5% Triton X-100 (Sigma-Aldrich) for 10 min at 4 °C followed by three freeze-thaw cycles (42 °C for 5 min and dry ice sprinkled with 2-Propanol (Sigma-Aldrich) for a minute per cycle). Coverslips were washed with a Buffer A (20 mM Hepes (pH 7.5), 100 mM KCl, 5 mM MgCl2, 10% BSA, 5% Glycerol, 0.1% Tween 20 and freshly added 1 mM DTT) for 20 min and blocked for 30 min at 37 °C using a Buffer C or in nuclease-free duplex buffer (idt). The CRISPR dCas9 complex was then added to the pre-blocked cells and incubated overnight at 37 °C. Cover slips were thoroughly washed and thereafter blocked for 15 minutes using a blocking buffer containing 10% Normal Donkey Serum in 1X Perm/Wash buffer (Buffer F) and immunostained overnight using anti-Cas9 primary antibodies at 4 ^{o}C and then 1 hour at room temperature. After washing with 1X Perm/Wash buffer, a secondary antibody was added for 45 min at room temperature. Coverslips were washed once with 1X Perm/Wash buffer, then twice with 1X PBS and counterstained with 1 µg/mL of DAPI nuclear stain in 1X PBS and incubated for 30 min. Finally, coverslips were washed three times with 1X Perm/Wash, then three times with 1X PBS and mounted onto glass slides using Prolong Diamond Antifade Mountant (Life Technologies).

Immunofluorescence: Fixed cells on poly-L-lysine-coated coverslips were permeabilized using 0.2% Triton X in 1X PBS for 15 min at room temperature. Cells were blocked for 15 min using a blocking Buffer F containing 10% Normal Donkey Serum in 1X perm wash buffer and immunostained overnight using primary antibodies at 4 °C and then 1 hour at room temperature. Cells were washed three times with 1X Perm wash buffer before secondary antibody was added for 45 min at room temperature. Coverslips were washed once with 1X Perm wash buffer, then twice with 1X PBS and counterstained with 1 µg/mL of DAPI nuclear stain in 1X PBS and incubated for 30 min. Finally, coverslips were washed three times with 1X Perm wash, then three times with 1X PBS and mounted onto glass slides using Prolong Diamond Antifade mountant (Life Technologies).

Table S4. Stellaris DNA oligonucleotide RNA FISH Probes

Probe target	Probe sequences (5' to 3')
**45 S pre-rRNA**	AAGTCGACAACCACTGGA, AAACCACGCTCCCCGGACC, CGCTCACCGAGAGCAGGCGGA, ACAGACCCGCGACGCTTCTTC, GCAAGCGAGGAGGACGACG, GGAACGACACACCACCGT, GTCTCGTCTCGTCTCACTC, AGCGACCGCAGCCACGAA,
**7SK snRNA**	GGGGTGACAGATGTCGCA, CAGATGAGCCGAATCAACCCTGGC, TTCGGGAGGGACGCACAT, CAGATCGCCCTCACATCC, GGGATGGTCGTCCTCTTC, CTTGACCGAAGACCGGTC, GCGCAGCTACTCGTATAC, TGGAGGTTCTAGCAGGGG, TGGACCTTGAGAGCTTGT, TGTCTGGAGTCTTGGAAG, AAAGGCAGACTGCCACAT,
**Total HIV mRNA**	TCCACAGATCAAGGATATCT, AAGTAGCCTTGTGTGTGGTA, GGTGTGTAGTTCTGCTAATC, AGGTCAGTGGATATCTGACC, TACTAGCTTGTAGCACCATC, TTCTACCTTATCTGGCTCAA, TCTTTGGGAGTGAATTAGCC, TGGTGTTCTCTCCTTTATTG, AGGCTCACAGGGTGTAACAA, CAAACCTCCACTCTAACACT, GCCACGTGATGAAATGCTAG, TTGAAGTACTCCGGATGCAG, TAGCAAGCTCGATATCAGCA, AAAGTCCCCAGCGGAAAGTC, TATATGCAGGATCTGAGGGC, GTACAGGCAAAAAGCAGCTG, TGAGGCTTAAGCAGTGGGTT, GCTTATATGCAGCATCTGAG, TGTAGCAAGCTCGATGTCAG, ACTTCTCTCTCAGGGTCATC, TCGGGCCATGTGATGAAATG, AAAGTCCCCAGCGGAAAGTC, TTGAAGTACTCCGGATGCAG, TACTAGCTTGTAGCACCATC, AGGTCAGTGGATATCTGATC, GGTGTGTAGTTCTGCTAATC, AAGTAGCCTTGTGTGTGGTA, TCCACAGATCAAGGATATCT, TTCGTTGGGAGTGAATTAGC, CTAACTTCTCTGGCTCAACT, AGGCTCACAGGGTGTAACAA, CAAACCTCCACTCTAACACT, CTGCTTATATGCAGGATCTG, ACCCAGTACAGGCAAAAAGC, CAGATCTGGTCTAACCAGAG, TTCCCTAGTTAGCCAGAGAG, GCTTTATTGAGGCTTAAGCA, AGAGTCACACAACAGACGGG, GTCTGAGGGATCTCTAGTTA, TGTGTAGTTCTGCCAATCAG, TTCTACCTTATCTGGCTCAA

RNA FISH probes were labeled at the 3′ end with either a 6-carboxytetramethylrhodamine (TAMRA), Quasar 670® or Fluorescein fluorophore (Biosearch Technologies, Novato, CA). Cells attached to poly-L-lysine-coated coverslips were fixed for 15 min at room temperature using 4% formaldehyde. Fixed cells were permeabilized for 1 hour at 4 °C using 70% ethanol. After permeabilization, the coverslips were rehydrated in wash buffer (Stellaris® RNA FISH Wash Buffer A (Biosearch Technologies Novato, CA) or (2× SSC, 10% de-ionized formamide) for 5 min placed on parafilm and hybridized overnight in a humidified chamber at 37 °C in hybridization Buffer G (10% dextran sulfate, 2X SSC, 10% deionized formamide) containing 35 μl – 50 μl of 2.5 μM RNA probes. Following hybridization, the cells were immersed once in a wash buffer (Stellaris® RNA FISH Wash Buffer A (Biosearch Technologies) or (2X SSC, 10% de-ionized formamide) for 30 min at room temperature and counterstained in a wash Buffer H (Stellaris® RNA FISH Wash Buffer A (Biosearch Technologies) or 2X SSC, 10% de-ionized formamide) containing 1 µg/ml of 4, 6-diamidino-2-phenylindole (DAPI) nuclear stain for 30 min at room temperature. Finally, coverslips were washed three times in Buffer I (Stellaris® RNA FISH Wash Buffer B (Biosearch Technologies or 2X SSC) for 5 min each at room temperature and mounted onto glass slides using Prolong Diamond Antifade mountant (Life Technologies).

SI Appendix References

1. C. Dobrowolski *et al.*, Entry of Polarized Effector Cells into Quiescence Forces HIV Latency. *MBio* **10** (2019).
2. J. K. Jadlowsky *et al.*, Negative elongation factor is required for the maintenance of proviral latency but does not induce promoter-proximal pausing of RNA polymerase II on the HIV long terminal repeat. *Mol Cell Biol* **34**, 1911-1928 (2014).
3. R. Pearson *et al.*, Epigenetic silencing of human immunodeficiency virus (HIV) transcription by formation of restrictive chromatin structures at the viral long terminal repeat drives the progressive entry of HIV into latency. *J. Virol.* **82**, 12291-12303 (2008).
4. U. O'Doherty, W. J. Swiggard, D. Jeyakumar, D. McGain, M. H. Malim, A sensitive, quantitative assay for human immunodeficiency virus type 1 integration. *J Virol* **76**, 10942-10950 (2002).
5. S. L. Butler, M. S. Hansen, F. D. Bushman, A quantitative assay for HIV DNA integration in vivo. *Nat Med* **7**, 631-634 (2001).
6. J. H. Simon *et al.*, The Vif and Gag proteins of human immunodeficiency virus type 1 colocalize in infected human T cells. *J Virol* **71**, 5259-5267 (1997).
7. R. A. Fouchier, B. E. Meyer, J. H. Simon, U. Fischer, M. H. Malim, HIV-1 infection of non-dividing cells: evidence that the amino-terminal basic region of the viral matrix protein is important for Gag processing but not for post-entry nuclear import. *Embo J* **16**, 4531-4539 (1997).
8. U. R. Mbonye *et al.*, Phosphorylation of CDK9 at Ser175 enhances HIV transcription and is a marker of activated P-TEFb in CD4+ T lymphocytes. *PLoS pathogens* **9** (2013).
9. C. Dingwall *et al.*, Human immunodeficiency virus 1 tat protein binds trans-activation-responsive region (TAR) RNA in vitro. *Proceedings of the National Academy of Sciences* **86**, 6925-6929 (1989).
